# Large Language Model Performance and Clinical Reasoning Tasks

**DOI:** 10.1001/jamanetworkopen.2026.4003

**Published:** 2026-04-13

**Authors:** Arya S. Rao, Kaiz P. Esmail, Richard S. Lee, Sharon Jiang, Bianca Arraiza Carlo, Jasleen Gill, Praneet Khanna, Ezra Kalmowitz, Basile Montagnese, Kimia Heydari, Qiao Jiao, Ethan Bott, Dan Nguyen, Grace Wang, Michael Hood, Adam B. Landman, Marc D. Succi

**Affiliations:** 1 Harvard Medical School, Boston, Massachusetts; 2 Medically Engineered Solutions in Healthcare Incubator, Innovation in Operations Research Center, Mass General Brigham, Boston, Massachusetts; 3 Harvard College, Cambridge, Massachusetts; 4 University of Missouri–Kansas City School of Medicine, Kansas City; 5 University of Massachusetts Chan Medical School, Worcester; 6 Massachusetts General Hospital, Boston; 7 Mass General Brigham, Boston, Massachusetts; 8 Brigham and Women’s Hospital, Boston, Massachusetts

## Abstract

**Importance:**

Large language models (LLMs) are increasingly marketed for clinical use, yet their ability to replicate full-spectrum clinical reasoning remains uncertain. Existing evaluations often rely on multiple-choice examinations that do not reflect the complexity of patient care.

**Objectives:**

To evaluate the longitudinal clinical reasoning ability of state-of-the-art LLMs and to introduce a multidimensional, clinically meaningful benchmark for clinical-grade artificial intelligence (AI).

**Design, Setting, and Participants:**

In this cross-sectional study, performance was evaluated using standardized clinical vignettes from the January 2025 update of MSD Manual vignettes. A total of 21 off-the-shelf LLMs, including recently released GPT-5, Claude 4.5 Opus, Gemini 3.0 Flash and Pro, and Grok 4, were evaluated. Models were assessed by medical student scorers in triplicate across sequential stages of the standard clinical workflow. Analyses were performed from January to December 2025.

**Main Outcomes and Measures:**

The primary outcome was the Proportional Index of Medical Evaluation for LLMs (PrIME-LLM) score, defined as the normalized polygonal area representing balanced accuracy across 5 domains of clinical reasoning as follows: differential diagnosis, diagnostic testing, final diagnosis, management, and miscellaneous clinical reasoning questions. Analyses including analyses of variance, *t* tests, and regression models were used to compare AI model performance and demographic associations.

**Results:**

LLMs were tested across 29 clinical vignettes (representing 16 254 responses in total). PrIME-LLM scores ranged from 0.64 (range, 0.63-0.65) (Gemini 1.5 Flash) to 0.78 (range, 0.77-0.79) (Grok 4), with reasoning-optimized models outperforming nonreasoning models and GPT models scoring highest overall. Differential diagnosis was less accurate than diagnostic testing, while final diagnosis, management, and miscellaneous reasoning were more accurate. Failure rates exceeded 0.80 (range, 0.90-1.00) for differential diagnosis in all models but were less than 0.40 (range, 0.09-0.39) for final diagnosis. Multimodal performance was robust; most LLM models showed improved accuracy with image inputs.

**Conclusions and Relevance:**

In this cross-sectional study of 21 LLMs, frontier LLMs achieved high accuracy on final diagnoses but performed poorly in generating differential diagnoses and navigating uncertainty relative to other reasoning stages. The PrIME-LLM framework provided greater separation than raw accuracy, revealing critical reasoning gaps obscured by traditional benchmarks. Thus, despite version-based improvements and advantages in reasoning-optimized models, off-the-shelf LLMs have not yet achieved the intelligence required for safe deployment and remain limited in demonstrating advanced clinical reasoning.

## Key Points

**Question:**

Can off-the-shelf large language models (LLMs) demonstrate reliable performance across the clinical workflow?

**Findings:**

In this cross-sectional study of 21 frontier LLMs tested on 29 standardized clinical vignettes, Grok 4 and other reasoning-optimized models achieved the highest scores, while Gemini 1.5 Flash performed lowest. Differential diagnosis consistently showed the weakest performance, while final diagnosis and management had stronger performances.

**Meaning:**

These findings suggest that despite progress, current LLMs remain limited in early diagnostic reasoning and cannot yet be relied on for unsupervised patient-facing clinical decision-making.

## Teaser

This cross-sectional study evaluates the end-to-end clinical reasoning ability of off-the-shelf large language models using standardized clinical vignettes and introduces a multidimensional comprehensive benchmark for clinical-grade artificial intelligence.

## Introduction

Large language models (LLMs) are rapidly gaining attention in medicine, powering tools that distill records, retrieve evidence, support diagnostic reasoning, generate documentation, and propose management.^1,2,3,4,5,6,7,8,9,10,11,12,13,14,15,16,17,18,19,20^ Vendors now actively market these systems for patient-facing clinical use,^21,22,23,24^ emphasizing high accuracy on benchmark tasks. Yet concerns about safety, integrity, and hallucinations remain,^25,26^ and the ability of LLMs to support end-to-end clinical reasoning remains unclear.

A challenge in evaluating LLMs for clinical use is determining whether their performance extends beyond static knowledge retrieval. To date, many of the most-cited evaluations of LLMs in medicine have centered around performance on multiple-choice licensing examinations, such as the US Medical Licensing Examination or specialty-specific board certification examinations.^27,28,29^ These formats are tempting to use as benchmarks because they are readily available, standardized, and easy to score. However, they fundamentally misrepresent the demands of clinical reasoning, including synthesizing evolving patient data, navigating diagnostic uncertainty, and making decisions across time.^30^ Consequently, they are inadequate for predicting how LLMs will function in clinical environments. Attempts to create more comprehensive benchmarks, including OpenAI’s HealthBench and similar synthetic evaluations,^31,32^ now use LLMs themselves to generate test cases and scoring rubrics; this raises substantial concerns about realism and measurement validity. Thus, there remains no single, clinically meaningful metric that captures an LLM’s end-to-end reasoning across the evolving stages of a patient encounter. As a result, models can excel in isolated domains yet harbor critical blind spots in others, undermining trust in clinical settings.

To address this gap, we sought a unified, interpretable score that would simultaneously penalize uneven performance and highlight balanced competence across diagnostic and decision-making tasks. In 2023, some authors of the current study pioneered the first stepwise evaluation framework for LLMs in clinical settings.^8^ Building on that work, we now introduce the Proportional Index of Medical Evaluation for LLMs (PrIME-LLM) score, a normalized, multidimensional metric to measure performance that rewards balanced performance across the clinical workflow. Here, we apply the PrIME-LLM framework to 21 frontier LLMs with the aim to provide the most comprehensive evaluation of longitudinal clinical reasoning to date and to establish a framework for evaluation of future off-the-shelf and custom models.

## Methods

This cross-sectional study was reviewed by the Mass General Brigham Institutional Review Board and granted a non–human participants research exemption. The study followed the Strengthening the Reporting of Observational Studies in Epidemiology (STROBE) reporting guideline.

### Artificial Intelligence LLMs

A total of 21 off-the-shelf LLMs were compared: GPT-4o,^33^ GPT-o1,^34^ GPT-o1-Pro,^35^ GPT-o3-Mini,^36^ GPT-4.5,^37^ and GPT-5^21^ (OpenAI); Claude 3.5 Haiku, Claude 3.5 Sonnet, Claude 3.7 Sonnet, Claude 3 Opus, and Claude 4.5 Opus^38^ (Anthropic); DeepSeek V3 and DeepSeek R1^39^ (DeepSeek); Gemini 1.5 Flash, Gemini 1.5 Pro, Gemini 2.0 Flash, Gemini 2.5 Pro, Gemini 3.0 Flash, and Gemini 3.0 Pro^40^ (Google DeepMind); and Grok 3^41^ and Grok 4^42^ (xAI). Detailed information about these models, including their release dates and capabilities, is presented in eTable 1 in Supplement 1.

### Clinical Vignettes

We assessed the accuracy of the 21 LLMs in working through 29 stepwise clinical vignettes from the January 2025 update of the MSD Manual (professional version, Merck Sharp & Dohme) web-based educational modules (eAppendix in Supplement 1).^43^ Each clinical vignette presents a structured case with history of present illness, review of systems, physical examination findings, and laboratory results. These peer-reviewed cases, developed by independent clinical experts, employ sequential select-all-that-apply questions to simulate the diagnostic process from differential diagnosis through testing and management planning.^43^ Each step of the clinical workflow—differential diagnosis, diagnostic testing, final diagnosis, management, and associated miscellaneous clinical reasoning questions—is presented sequentially, as shown in eFigure 1 in Supplement 1. These 5 domains (ie, question types) match the structure of the MSD Manual,^43^ in which each stage is treated as a required and integral component of a single, unified clinical workflow. Each vignette included basic patient demographics (eg, age and sex), which allowed us to examine model performance across these characteristics.

### LLM Prompting

Models received case transcripts sequentially with context preserved via application programming interfaces (APIs) or web interfaces (GPT-o1, GPT-o1-Pro, and GPT-o3-Mini). Questions requiring image interpretation were excluded from scoring for models without multimodal capabilities. We evaluated each LLM by presenting vignettes in a stepwise manner that preserved clinical context and maintained continuity throughout the arc of clinical reasoning. Vignette prompts were presented in a direct question-and-answer format, exactly as presented in the MSD Manual.^43^

For API-based models, we programmatically managed context by constructing each API request to include the complete conversation history, sending an array containing the following: (1) a system message defining the clinical task, (2) all previous user prompts (patient presentation and questions), and (3) all previous model responses. For web-based interfaces that inherently maintain a conversation state, we entered each case component sequentially within a single session, allowing the platform’s native context management to preserve the diagnostic narrative. Models were prompted using their default settings; where a reasoning setting was available, it was not enabled in order to assess the base models only. For all models, optional real-time web search, browsing, and retrieval features were explicitly disabled when available. Each vignette was evaluated in triplicate. All replicates and vignettes were parsed independently. To ensure comparability, optional features such as real-time search were disabled across all models.

### Scoring

Model outputs were scored by medical student evaluators (K.P.E., R.S.L., P.K., E.K., B.M., Q.J., E.B., D.N., and G.W.) against the MSD Manual^43^ answer keys. Each model response was scored by a single evaluator using a deterministic rubric that mapped the model’s free-text output to the predefined multiple-choice answer options. Full credit was awarded only when all correct answers were explicitly included and incorrect options were excluded (eFigure 2 in Supplement 1). Each vignette was evaluated in triplicate using independent model runs to capture model stochasticity; these replicates were scored independently and typically by different evaluators. Raw accuracy was defined as the mean proportion correct across 3 independent replicates per question. Question type–level accuracies per vignette were used to calculate PrIME-LLM scores.

### PrIME-LLM Score

To capture longitudinal clinical reasoning in a single interpretable metric, we developed the PrIME-LLM score. Performance across the 5 domains of clinical reasoning evaluated in this study (differential diagnosis, diagnostic testing, final diagnosis, management, and miscellaneous clinical reasoning questions) was first visualized as a radar plot, with each vertex representing accuracy in 1 domain.

The PrIME-LLM score is calculated as the area of the model’s polygon divided by the area of the full-scale reference polygon. The full-scale reference polygon corresponds to a model scoring 100% in all 5 categories. Unlike simple arithmetic averaging (as is), this area-based approach rewards balanced competence. Scores are represented as proportions ranging from 0 to 1, with higher values indicating stronger and more consistent clinical reasoning. Robust diagnostic reasoning requires proficiency across the full diagnostic and management process rather than isolated strengths; to achieve a high PrIME-LLM score, a model must perform well across all domains. Thus, domains were weighted equally. A PrIME-LLM score can be calculated for any clinical benchmark on which there is a multitask component; the polygonal area corresponds to the area of the polygon with vertices equal to the number of tasks.

### Statistical Analysis

All analyses were 2-sided, with α = .05. Model-level accuracy was compared using a 1-way repeated-measures analysis of variance (ANOVA) with model as a within-subjects factor and question as the repeated factor (each question was answered by all models). When the omnibus test was significant, we conducted pairwise post hoc comparisons between models using paired *t* tests with Holm correction to control the familywise error rate. PrIME-LLM scores (radar area based) were compared across models using a 1-way between-subjects ANOVA, because each PrIME-LLM value is defined at the level of a full replicate rather than an individual question. For these analyses, we used the Tukey honestly significant difference (HSD) test for all pairwise post hoc contrasts. Welch *t* tests were used for planned comparisons between image vs nonimage performance in multimodal models and between reasoning vs nonreasoning models. At the vendor-family level, we computed mean performance scores for each family and compared them using 1-way ANOVA with the Tukey HSD test for all pairwise post hoc contrasts, providing familywise error control in this between-groups setting. Associations with demographics were examined using ordinary least-squares regression including age, sex, question type, and reasoning capability. Regression models for question-level accuracy included a random intercept for question identifier (ID) (case) to account for the nested, repeated-measures structure (multiple models per question). Analyses were run in Python, version 3.11 (Pallets Projects; Python Software Foundation), with the pandas/numpy (data handling), scipy (*t* tests), statsmodels (repeated-measures ANOVA, regression, and multiple-comparisons correction), and seaborn/matplotlib (visualization) libraries. Analyses were performed from January to December 2025.

## Results

### PrIME-LLM Scores Across 21 Frontier LLMs

**Figure 1.**
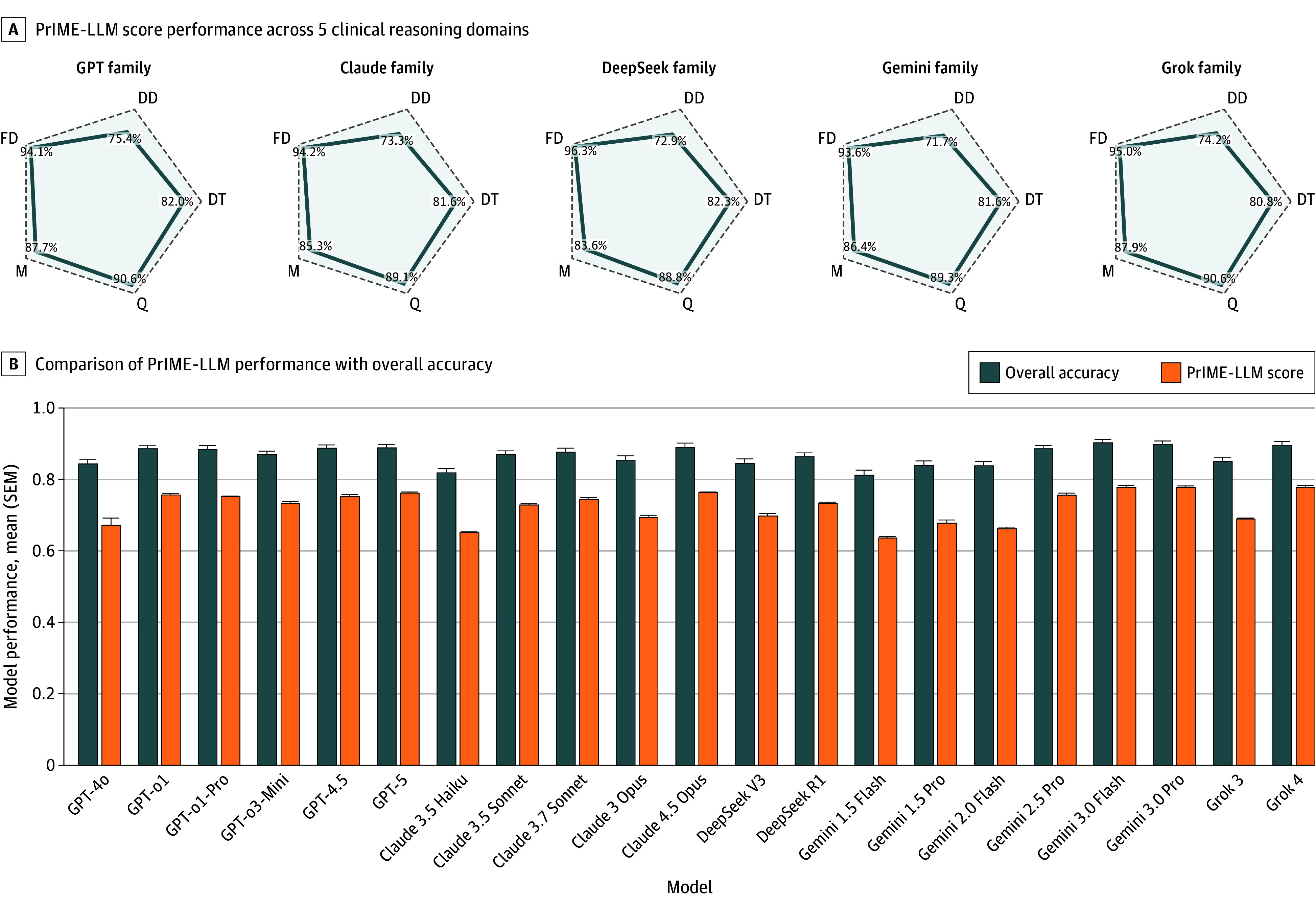
Proportional Index of Medical Evaluation for Large Language Model (PrIME-LLM) Scores and Comparison of PrIME-LLM Scores and Overall Accuracy for Tested Clinical Vignettes A, Radar plots show accuracy across 5 domains of clinical reasoning: differential diagnosis (DD), diagnostic testing (DT), final diagnosis (FD), management (M), and miscellaneous clinical reasoning questions (Q). PrIME-LLM scores were calculated as the normalized polygonal area, summarizing balanced performance across tasks. B, Bar graphs show a comparison of overall accuracy vs PrIME-LLM scores for the tested clinical vignettes. Error bars indicate SEMs.

We evaluated 21 LLMs on 29 standardized clinical vignettes (representing 16 254 responses in total),^8^ assessing performance across 5 domains: differential diagnosis, diagnostic testing, final diagnosis, management, and miscellaneous clinical reasoning questions. Radar plots (Figure 1A and eFigures 3 and 4 in Supplement 1) represent domain accuracies; the PrIME-LLM score calculates the polygonal area to summarize overall and balanced performance. Across models, the most consistent deficits were observed in differential diagnosis and diagnostic testing, whereas final diagnosis and management generally scored higher.

**Figure 2.**
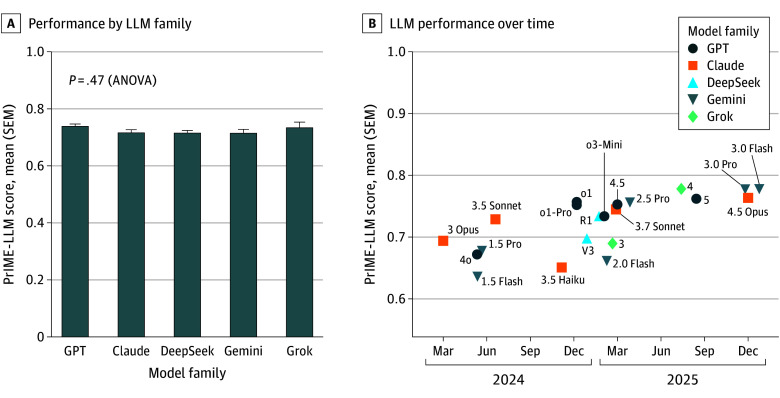
Proportional Index of Medical Evaluation for Large Language Model (PrIME-LLM) Scores by Model Family and PrIME-LLM Scores by Release Date A, Bar graph shows mean PrIME-LLM scores grouped by developer family (GPT, Claude, DeepSeek, Gemini, and Grok). Error bars indicate SEMs. B, Scatter plot shows version-based trajectories within each model family, showing successive releases in chronological order. ANOVA indicates analysis of variance.

PrIME-LLM scores differed significantly across models (*F*_20,42_ = 50.96; *P* < .001) (Figure 2). The Tukey HSD results showed a top-performing cluster that included Grok 4, GPT-5, GPT-4.5, Claude 4.5 Opus, Gemini 3.0 Flash, and Gemini 3.0 Pro, with many pairwise differences among these leaders that were not significant. Within families, newer releases generally performed better: GPT-4.5, GPT-5, and the GPT-o1 series outperformed GPT-4o; Gemini 3.0 Flash and Gemini 3.0 Pro outperformed the Gemini 1.5 and Gemini 2.0 series; and Grok 4 outperformed Grok 3. At the lower end, Claude 3.5 Haiku had the lowest PrIME-LLM scores, substantially below most models; it was only comparable to GPT-4o and the early Gemini baselines (Gemini 1.5 Flash, Gemini 1.5 Pro, and Gemini 2.0 Flash). eFigure 5 in Supplement 1 provides a heat map of Tukey HSD pairwise comparisons, and eTable 2 in Supplement 1 presents all PrIME-LLM scores.

Figure 1B compares PrIME-LLM scores with overall accuracy, the traditional summary metric. Whereas mean overall accuracy values clustered narrowly between 0.81 and 0.90 with SEMs consistently up to 0.02, mean PrIME-LLM scores revealed wider separation, distinguishing high-performing models (eg, Grok 4: 0.78 [range, 0.77-0.79]) from weaker ones (eg, Gemini 1.5 Flash: 0.64 [range, 0.63-0.65]). These findings illustrate how raw accuracy obscures important differences in multidimensional reasoning that are captured by the PrIME-LLM framework (Spearman *r* = 0.98; *P* = 3.6 × 10^−15^). All models demonstrated relatively low SEM values, indicating that model performance was consistent across replicates (eFigure 6 in Supplement 1).

We also compared reasoning-optimized models (GPT-o1, GPT-o1-Pro, GPT-o3-Mini, GPT-5, DeepSeek R1, Claude 3.7 Sonnet, Claude 4.5 Opus, Gemini 2.5 Pro, Gemini 3.0 Pro, Gemini 3.0 Flash, and Grok 4) with models not advertised as having reasoning capabilities. Reasoning models were defined as those models whose architecture, training objectives, or inference-time procedures (or a combination thereof) were explicitly optimized by the developer for multistep latent computation toward a correct final output, beyond what emerges from scale alone. A 2-sample *t* test revealed a significant difference in performance between the reasoning group (mean [SEM], 0.76 [0.003]; n = 33) and the nonreasoning group (mean [SEM], 0.67 [0.01]; n = 30) (*t*_61_ = 9.98; *P* = 1.95 × 10^−12^) (eFigures 7 and 8 in Supplement 1). The effect size was large, with Cohen *d* of 2.60 and Hedges’ *g* of 2.57 (95% CI, 1.90-3.23). The probability that a randomly selected score from the reasoning group would exceed one from the nonreasoning group (common language effect size) was 0.99 (95% CI, not available). The 95% CI for the mean was 0.75 to 0.76 for the reasoning group and 0.67 to 0.70 for the nonreasoning group. Collectively, these results indicate a robust and highly reliable difference in outcomes between the 2 groups.

### Performance by Question Type and Modality

A 1-way ANOVA revealed an association between item type and proportion correct across all evaluated LLMs. Post hoc Tukey HSD tests demonstrated consistent and robust patterns in pairwise comparisons. For nearly every model (including GPT-4o, GPT-4.5, GPT-5, GPT-o1, GPT-o1-Pro, GPT-o3-Mini; both Claude 3.5 and 4.5 variants; DeepSeek V3 and R1; Gemini 1.5 Flash, Gemini 1.5 Pro, Gemini 2.0 Flash, Gemini 2.5 Pro, and Gemini 3.0 Flash; and Grok 3), final diagnosis items were solved with significantly higher accuracy than diagnostic testing items, as indicated by consistently positive and significant mean differences in final diagnosis vs diagnostic testing items (mean difference, 0.12 [95% CI, 0.04-0.19] for GPT-4o; 0.16 [95% CI, 0.13-0.18] for Claude 3.5 Haiku; and 0.15 [95% CI, 0.11-0.19] for DeepSeek V3). Moreover, final diagnosis items generally outperformed differential diagnosis, management, and miscellaneous clinical reasoning question types as well, with the reverse rarely observed in any significant pairwise comparison. For diagnostic testing items, accuracy was also consistently higher than for differential diagnosis items (eg, Claude 4.5 Opus: diagnostic testing performed better than differential diagnosis, with a mean difference of 0.05 [95% CI, 0.03- 0.07]), while miscellaneous and management types typically fell intermediate. In summary, item type was a major and consistent factor in LLM performance, with the lowest accuracy observed for differential diagnosis items.

We further evaluated whether multimodal, image-capable models performed with higher accuracy on questions in which images were provided compared with questions where images were not (ie, text only). Eighteen multimodal models were assessed across vignettes containing images such as chest radiographs, computed tomography scans, and electrocardiograms. Accuracy on nonimage questions was generally more consistent, while performance on image-based questions varied by model. GPT-o3-Mini (difference, 0.08 [95% CI, 0.01-0.14]; *P* = .02), Claude 3 Opus (difference, 0.07 [95% CI, 0.01-0.15]; *P* = .02), and GPT-4.5 (difference, 0.10 [95% CI, 0.05-0.14]; *P* < .001) all demonstrated significantly higher accuracy on image-based items compared with text-only items, with GPT-4.5 achieving nearly a 5-point increase (eFigure 9 in Supplement 1). In newer models, significantly greater accuracy was also seen in image-based items: Gemini 2.5 Pro (difference, 0.06 [95% CI, 0.01-0.12]; *P* = .03), Gemini 3.0 Pro (difference, 0.09 [95% CI, 0.06-0.12]; *P* < .001), Gemini 3.0 Flash (difference, 0.06 [95% CI, 0.01-0.11]; *P* = .01), and Grok 4 (difference, 0.07 [95% CI, 0.02-0.11]; *P* = .002). In contrast, all other models showed no significant differences between modalities.

### Analysis of Factors Associated With LLM Performance

A mixed-effects linear regression was conducted to assess predictors of question-level accuracy, incorporating question ID as a random effect. Accuracy was significantly higher for final diagnosis items compared with the reference group (β coefficient, 0.13 [95% CI, 0.12-0.15]; *P* < .001), while accuracy was lower for differential diagnosis items (β coefficient, −0.08 [95% CI, −0.09 to −0.06]; *P* < .001). Management and miscellaneous item types were also associated with higher accuracy (β coefficient, 0.05 [95% CI, 0.04-0.07] for management and 0.08 [95% CI, 0.07-0.09] for miscellaneous items; both *P* < .001) (eTables 3 and 4 in Supplement 1). Reasoning models demonstrated superior accuracy (β coefficient, 0.04 [95% CI, 0.03-0.05]; *P* < .001), whereas sex and age were not significant predictors. Results were consistent in a model-level analysis using ordinary least-squares regression, where reasoning models exhibited higher PrIME-LLM radar area scores (β coefficient, 0.07 [95% CI, 0.06-0.09]; *P* < .001), accounting for approximately 63% of variance. Together, these findings underscore the influence of question type on accuracy and highlight the overall performance advantage of reasoning models.

### Model Failure Rates

**Figure 3.**
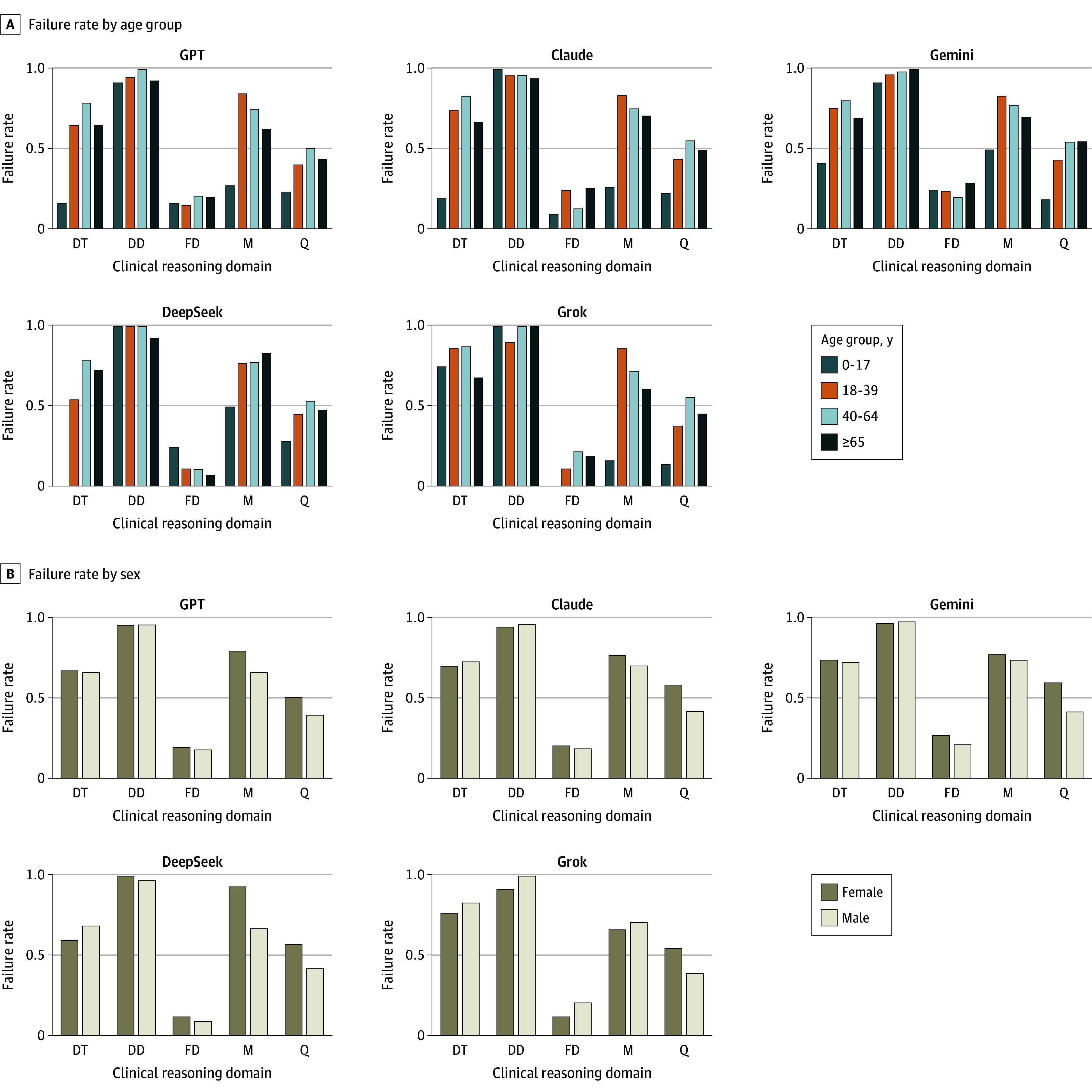
Bar Graphs of Failure Rates of Tested Large Language Models A, Failure rate by age group, defined as the proportion of questions not answered fully correctly. B, Failure rate by patient sex. DD indicates differential diagnosis; DT, diagnostic testing; FD, final diagnosis; M, management; Q, miscellaneous clinical reasoning questions.

The model failure rate, defined as the proportion of questions not answered fully correctly, provides a measure of reliability that complements the PrIME-LLM score (eFigure 10 in Supplement 1). Across models, failure rates were highest for differential diagnosis, often exceeding 0.80, and were lowest for final diagnosis, which usually was successfully answered based on the provided information of each vignette and question item (Figure 3 and eTable 5 in Supplement 1). Diagnostic testing, management, and miscellaneous clinical reasoning questions showed intermediate values with variability between models. Case-level analysis revealed that some vignettes were consistently difficult across all families, while others were less challenging, suggesting broad limitations in reasoning rather than domain-specific gaps. When stratified by demographics, failure rates were highest in young adult and middle-aged cases, particularly for differential diagnosis and management, while pediatric cases showed lower rates except in differential diagnosis (eFigure 11 and eTable 6 in Supplement 1). No consistent sex-based differences were observed.

## Discussion

The promise of LLMs in clinical medicine lies in their potential to augment—not replace—physician reasoning. This study establishes the first benchmark for longitudinal clinical reasoning (to our knowledge) and introduces the PrIME-LLM framework, a multidimensional metric designed to capture performance across the full arc of diagnostic and management tasks.

Our evaluation suggests that despite rapid advances in pattern recognition and knowledge retrieval, current LLMs still lack the reasoning processes needed for safe clinical use. The consistent gap between differential diagnosis and final diagnosis highlights how differently these systems process information compared with physicians. Clinicians preserve uncertainty and iteratively refine differential diagnoses, whereas LLMs collapse prematurely onto single answers, a limitation that persists across model generations. Their weak performance on differential diagnosis, consistent with a prior study from authors of the current work,^8^ suggests these limitations persist across early and state-of-the-art models. The risk is not just that LLMs are sometimes wrong but that their reasoning is brittle precisely where uncertainty and nuance matter most. Benchmarks that reward only correct final answers risk reinforcing this shortcutting, widening the gap between marketing claims and the skills actually required at the bedside.

Using the PrIME-LLM framework, we found that models optimized for reasoning showed modest but significant gains over nonreasoning models; improvements were incremental and did not resolve the persistent gap in differential diagnosis generation. Family-level analyses showed nonsignificant differences in performance between developers; thus, we caution against interpreting family-level analysis as guidance for model selection, as this can obscure meaningful differences between individual models. Multimodal gains were limited and inconsistent, underscoring how far current models remain from reliably handling the multimodal clinical data, including various types of images, central to practice.

### Limitations

Importantly, this study evaluates off-the-shelf LLMs without external augmentation to enable controlled, comparable benchmarking across model families. In addition, several limitations should be noted. First, models were accessed through a mixture of API-based and web-based interfaces, and optional web search and reasoning was disabled. Second, because the standardized clinical vignettes used are publicly available, prior exposure during model pretraining cannot be fully excluded. Third, the evaluation does not incorporate model augmentations such as retrieval-augmented generation, guideline access, calculators, or agentic tool use, which may improve performance in clinical settings, particularly for downstream tasks. Accordingly, the results reflect baseline longitudinal clinical reasoning rather than maximal achievable performance. The PrIME-LLM framework is intended to support future evaluations of newer models and augmented systems to assess how added capabilities affect reasoning across the clinical workflow. Finally, PrIME-LLM is not intended to establish equivalence or inferiority relative to clinicians, and the present study was not designed to answer human comparison questions.

Most importantly, the findings of this study caution against vendor claims that general purpose, off-the-shelf LLMs are ready for patient-facing clinical use.^21,22,23^ While strong performance on final diagnosis tasks may create that impression, persistent failures in generating differential diagnoses and navigating uncertainty show that LLMs cannot yet be trusted in frontline decision-making. Marketing LLMs as diagnostic agents risks fostering false confidence precisely where they are least reliable. Their most responsible role today is targeted, clinician-supervised use in low-uncertainty tasks. Without such constraints, premature deployment could lead to individual errors in patient care that, when compounded, erode the systems of clinical reasoning that protect patients from harm.

## Conclusions

In this cross-sectional study of 21 LLMs, frontier LLMs achieved high accuracy on final diagnoses but they performed poorly in generating differential diagnoses and navigating uncertainty relative to other reasoning stages. The PrIME-LLM framework provided greater separation than raw accuracy, revealing critical reasoning gaps obscured by traditional benchmarks. As commercial systems increasingly market reasoning capabilities and move toward clinical deployment, PrIME-LLM scores provide an independent, reproducible, and extensible benchmark to track progress, expose persistent limitations, and guide safe integration into health care practice.

## Notes

Supplement 1. **eTable 1.** Comparison of Models Used in Systematic Analyses **eAppendix.** MSD Manual Clinical Vignettes **eFigure 1.** Stepwise Evaluation Framework for MSD Manual Clinical Vignettes **eFigure 2.** Model Scoring Schema **eFigure 3.** Radar Plots for All Tested Models **eFigure 4.** Radar Plots for LLM Vendors Using Only API-Based Models **eFigure 5.** Heat Map of Pairwise Tukey Comparisons of PrIME-LLM Scores Between Models **eTable 2.** PrIME-LLM Scores and SEM of Tested Models **eFigure 6.** Average Accuracy and PrIME-LLM Calculations by Model and Family With API-Only Models **eFigure 7.** PrIME-LLM Score Comparison Between Reasoning-Designated Models and Nonreasoning-Designated Models **eFigure 8.** PrIME-LLM Score Comparison Between Reasoning-Designated Models and Nonreasoning-Designated Models Using Only API-Based Models. **eFigure 9.** PrIME-LLM Score Comparison Between Questions Involving Use of an Image and Text-Only Questions **eTable 3.** Multivariable Linear Regression Modeling the Probability of Question-Level Accuracy **eTable 4.** Multivariable Linear Regression of PrIME-LLM Scores by Model Features **eFigure 10.** Failure Rates of Tested Models by Question Type and Case Number **eTable 5.** Failure Rates by Age Group and Question Type **eFigure 11.** Failure Rates of Tested Models by Question Type and Case Number Including Only API-Based Models **eTable 6.** Failure Rates by Patient Sex and Question Type
eReferences

Supplement 2.
Data Sharing Statement
