# Supplementary material for: Large Language Model Performance and Clinical Reasoning Tasks
Source: JAMA Netw Open. 2026 Apr 13;9(4):e264003. doi: 10.1001/jamanetworkopen.2026.4003 (PMC13077515; doi:10.1001/jamanetworkopen.2026.4003)
Supplement: Supplement 1. — eTable 1. Comparison of Models Used in Systematic Analyses eAppendix. MSD Manual Clinical Vignettes eFigure 1. Stepwise Evaluation Framework for MSD Manual Clinical Vignettes eFigure 2. Model Scoring Schema eFigure 3. Radar Plots for All Tested Models eFigure 4. Radar Plots for LLM Vendors Using Only API-Based Models eFigure 5. Heat Map of Pairwise Tukey Comparisons of PrIME-LLM Scores Between Models eTable 2. PrIME-LLM Scores and SEM of Tested Models eFigure 6. Average Accuracy and PrIME-LLM Calculations by Model and Family With API-Only Models eFigure 7. PrIME-LLM Score Comparison Between Reasoning-Designated Models and Nonreasoning-Designated Models eFigure 8. PrIME-LLM Score Comparison Between Reasoning-Designated Models and Nonreasoning-Designated Models Using Only API-Based Models. eFigure 9. PrIME-LLM Score Comparison Between Questions Involving Use of an Image and Text-Only Questions eTable 3. Multivariable Linear Regression Modeling the Probability of Question-Level Accuracy eTable 4. Multivariable Linear Regression of PrIME-LLM Scores by Model Features eFigure 10. Failure Rates of Tested Models by Question Type and Case Number eTable 5. Failure Rates by Age Group and Question Type eFigure 11. Failure Rates of Tested Models by Question Type and Case Number Including Only API-Based Models eTable 6. Failure Rates by Patient Sex and Question Type eReferences [file jamanetwopen-e264003-s001.pdf]

Supplementary Online Content

Rao AS, Esmail KP, Lee RS, et al. Large language model performance and clinical reasoning tasks. *JAMA Netw Open*. 2026;9(3):e264003. doi:10.1001/jamanetworkopen.2026.4003

eTable 1. Comparison of Models Used in Systematic Analyses

eAppendix. MSD Manual Clinical Vignettes

eFigure 1. Stepwise Evaluation Framework for MSD Manual Clinical Vignettes

eFigure 2. Model Scoring Schema

eFigure 3. Radar Plots for All Tested Models

eFigure 4. Radar Plots for LLM Vendors Using Only API-Based Models

eFigure 5. Heat Map of Pairwise Tukey Comparisons of PrIME-LLM Scores Between Models

eTable 2. PrIME-LLM Scores and SEM of Tested Models

eFigure 6. Average Accuracy and PrIME-LLM Calculations by Model and Family With API-Only Models

eFigure 7. PrIME-LLM Score Comparison Between Reasoning-Designated Models and Nonreasoning-Designated Models

eFigure 8. PrIME-LLM Score Comparison Between Reasoning-Designated Models and Nonreasoning-Designated Models Using Only API-Based Models.

eFigure 9. PrIME-LLM Score Comparison Between Questions Involving Use of an Image and Text-Only Questions

eTable 3. Multivariable Linear Regression Modeling the Probability of Question-Level Accuracy

eTable 4. Multivariable Linear Regression of PrIME-LLM Scores by Model Features

eFigure 10. Failure Rates of Tested Models by Question Type and Case Number

eTable 5. Failure Rates by Age Group and Question Type

eFigure 11. Failure Rates of Tested Models by Question Type and Case Number Including Only API-Based Models

eTable 6. Failure Rates by Patient Sex and Question Type

eReferences

This supplementary material has been provided by the authors to give readers additional information about their work.

eTable 1. Comparison of Models Used in Systematic Analyses. Each model family is coded with a different color. Within each family, the models are sorted from oldest to newest release date. Descriptions are reproduced from the developer's model information page or press release.

Model	Release Date	Description	Window / Output Tokens	Knowledge Cutoff	Multimodal
**GPT-4o**[1]	May 13, 2024	Fast, intelligent, flexible model with versatility and high intelligence	128,000 / 16,384	Sep. 2023	Yes
**GPT-o1**[2]	Dec. 5, 2024	Previous full reasoning model, excels at complex, multi-step tasks	200,000 / 100,000	Sep. 2023	Yes
**GPT-o1-pro**[3]	Dec. 5, 2024	Previous full reasoning model with more compute for better responses	200,000 / 100,000	Sep. 2023	Yes
**GPT-o3-mini**[4]	Jan. 31, 2025	A small model alternative to the most powerful reasoning model	200,000 / 100,000	Sep. 2023	Yes
**GPT-4.5**[5]	Feb. 27, 2025	A research preview of the strongest model for chat	128,000 / 16,000	Oct. 2023	Yes
**GPT-5**[6]	Aug. 7,	Best for coding and	400,000 /	Sep. 2024	Yes

© 2026 Rao AS et al. *JAMA Network Open*.

	2025	agentic tasks across domains, with optimization for health-related questions	128,000		
**Claude 3.0 Opus**[7]	Mar. 4, 2024	Powerful for complex tasks; top-level intelligence, fluency, and understanding	200,000 / 4,096	Aug. 2023	Yes
**Claude 3.5 Sonnet**[7]	Jun. 20, 2024	Previous intelligent model, high intelligence and capability	200,000 / 8,192	Apr. 2024	Yes
**Claude 3.5 Haiku**[7]	Nov. 4, 2024	Fastest model with intelligence at blazing speeds	200,000 / 8,192	Jul. 2024	No, text only
**Claude 3.7 Sonnet**[7]	Feb. 24, 2025	High-performance model with toggleable extended thinking	200,000 / 64,000	Nov. 2024	Yes
**Claude 4.5 Opus**[7]	Nov. 24, 2025	Intelligent, efficient, and better at coding and everyday tasks	300,000 / 64,000	May 2025	Yes
**DeepSeek V3**[8]	Dec. 26, 2024	An open-source model designed for efficient	64,000 / 8,000	Jul. 2024	No, text only

© 2026 Rao AS et al. *JAMA Network Open*.

		inference and training			
**DeepSeek R1**[8]	Jan. 20, 2025	A first-generation reasoning model to solve complex reasoning tasks across multiple domains	64,000 / 8,000	Jul. 2024	No, text only
**Gemini 1.5 Flash**[9]	May 14, 2024	Fast and versatile performance across a diverse variety of tasks	32,000 / 8,192	Nov. 2023	Yes
**Gemini 1.5 Pro**[9]	May 23, 2024	Good at complex reasoning tasks requiring more intelligence	128,000 / 8,192	May 2024	Yes
**Gemini 2.0 Flash**[9]	Feb. 5, 2025	Most powerful thinking model with maximum response accuracy and state-of-the-art performance	2,000,000 / 8,192	Jun. 2024	Yes
**Gemini 2.5 Pro**[9]	Jun. 17, 2025	State-of-the-art thinking model	1,048,576 / 65,536	Jan. 2025	Yes
**Gemini 3.0 Pro**[9]	Nov. 18, 2025	Best model for multimodal understanding with foundational state-of-the-art reasoning	1,048,576 / 65,536	Jan. 2025	Yes

© 2026 Rao AS et al. *JAMA Network Open*.

**Gemini 3.0 Flash**[9]	Dec. 17, 2025	Balanced for speed, scale, and frontier intelligence	1,048,576 / 65,536	Jan. 2025	Yes
**Grok 3**[10]	Feb. 17, 2025	Most advanced model, blending superior reasoning with extensive pre-training knowledge	1,000,000 / 16,000	Nov. 2024	Yes
**Grok 4**[11]	Jul. 9, 2025	Most intelligent model in the world	256,000 combined	Dec. 2024	Yes

© 2026 Rao AS et al. *JAMA Network Open*.

eAppendix 1. MSD Manual Vignettes

Case 1: Chest Pain in 49-Yr-Old Woman

Background

History of Present Illness:

A 49-yr-old woman comes to the emergency department because of chest pain. She says she was in her usual state of health until the day before, when she awoke at 6 am with the sudden onset of sharp left-sided chest pain. Pain has been constant and radiates to the center of her chest. It is not worsened by exertion, movement, food, activity, or respiration. She also had shortness of breath and chills but no cough, nausea, or diaphoresis. She took 400 mg of naproxen without relief. The pain has persisted, prompting her to come to the emergency department now. She has never had chest pain before this episode and has no known history of cardiac or pulmonary disease.

Review of systems:

General: Chills as noted in History of Present Illness. She thought she had a fever but did not take her temperature.

Skin: Noncontributory

HEENT: No recent URI symptoms.

Pulmonary: As per History of Present Illness.

Cardiovascular: Chest pain as per History of Present Illness. No palpitations or syncope. She has noted dyspnea with climbing stairs since chest pain began; dyspnea resolves quickly with rest and is not worsened by lying flat.

Gastrointestinal: No abdominal pain, nausea, or vomiting. Appetite and bowel movements are normal.

Genitourinary: Minimal spotting after recent menses.

Musculoskeletal: She has no current musculoskeletal symptoms, although she has noted intermittent left leg swelling and discomfort for the past year. There is no history of injury or

© 2026 Rao AS et al. *JAMA Network Open*.

recent immobilization. The last time she had swelling and discomfort in her left leg was about 10 days ago.

Neurologic: Noncontributory

Psychiatric: Noncontributory

Past Medical History:

Medical history: Menorrhagia due to uterine fibroids, with secondary iron-deficiency anemia; endometriosis; right breast fibroadenoma. No history of blood clots or clotting disorder.

Surgical history: Laparoscopy and lysis of adhesions for endometriosis; tubal ligation; excision of breast fibroadenoma.

Medications: None

Allergies: None

Family history: Mother was recently diagnosed with stage IV colon carcinoma; brother has schizophrenia.

Social history: No tobacco or alcohol use; former cocaine and heroin use (quit both more than 20 yr ago); works for the hospital in the food services department. No recent travel.

Physical Examination:

General appearance: Appears anxious and in some pain. No cyanosis or respiratory distress.

Vital signs:

Temperature 36.8¬∞ C

Pulse 107 beats/min

BP right arm 130/72 mm Hg

Respirations: 16/min

O2 saturation 96% on room air

© 2026 Rao AS et al. *JAMA Network Open*.

Skin: No lesions or rashes.

HEENT: Mucus membranes moist, no oral lesions, patent nares.

Pulmonary: Lungs clear to auscultation bilaterally. No crackles or wheezes.

Cardiovascular: Elevated rate, regular rhythm. No murmurs, rubs, or gallops. No tenderness to palpation. No jugular venous distention. Normal carotid pulse.

Gastrointestinal: bdomen nontender and nondistended with normal bowel sounds.

Genitourinary: Uterus palpated just below the umbilicus. No blood in vaginal vault.

Musculoskeletal: No joint deformity, full range of motion without pain. Lower extremities: no swelling, erythema, or tenderness.

Neurologic: Cranial nerves intact; muscle strength and sensation intact throughout.

Mental status: Alert and oriented to person, place, and time.

Questions

Question 1 | Type: DIFF

At this time, which of the following are common and/or dangerous differential diagnoses that cannot be excluded based on history and physical examination of this patient? (You may select more than one option.)

Aortic dissection
Community-acquired pneumonia
COPD exacerbation
Costochondritis
Gastroesophageal reflux
Pulmonary embolism
Anxiety
Myocardial infarction (MI)
Myocarditis

© 2026 Rao AS et al. *JAMA Network Open*.

Severe aortic stenosis

Viral pericarditis

Question 2 | Type: **DIAG**

Which of the following steps are essential to do before the patient leaves the ED? (You may select more than one option.)

Arterial blood gas values

Chest x-ray, portable

Chest x-ray, posteroanterior and lateral

CT scan of the chest

Electrocardiogram (ECG)

Serum D-dimer levels

Complete blood count (CBC)

Pro-brain natriuretic peptide

Serum troponin concentration

Complete metabolic panel (CMP)

Question 3 | Type: **DX**

Image: *1_DX_ECG*

Results of appropriate laboratory testing return and show:

Serum D-dimer level: 395 ng/mL [2.16 nmol/L]

Hemoglobin: 9.7 g/dL

Platelets: 330,000/mcL

WBC: 10,600/mcL

Serum troponin I level: 0.05 ng/mL [0.05 mcg/L]

ECG is obtained and is shown.

Which of the following is a correct interpretation of this patient's ECG?

Sinus tachycardia with diffuse ST elevations and PR depressions

Sinus tachycardia with ST segment elevations in leads II, III, and aVF

© 2026 Rao AS et al. *JAMA Network Open*.

Sinus tachycardia with ST segment depressions in leads II, III, and aVF

Sinus tachycardia with S1Q3T3 sign and nonspecific ST-T wave changes

Question 4 | Type: DX

Given the previous findings, which of the following are the most appropriate steps to guide diagnosis and treatment?

Cardiac catheterization

CT scan of the chest with IV contrast

Pulmonary function testing

Doppler ultrasonography of the legs

Question 5 | Type: DX

Image: *1_DX_CT*

CT scan of the chest with IV contrast is obtained and shows a filling defect in bilateral upper and lower segmental pulmonary arteries on pulmonary arterial phase.

At this time what is the most likely diagnosis? (You may select only one option.)

Community-acquired pneumonia

Costochondritis

Myocardial infarction

Myocarditis

Pulmonary embolism (PE)

Viral pericarditis

Question 6 | Type: MANG

At this time, which of the following is the most appropriate treatment? (You may select more than one option.)

Admit the patient to the hospital for further treatment

Discharge the patient with close outpatient follow-up

Heparin 80 U/kg IV bolus followed by 18 U/kg/h IV infusion

Warfarin 5 mg po once/day at bedtime

© 2026 Rao AS et al. *JAMA Network Open*.

Rivaroxaban 15 mg po bid

Acetaminophen 650 mg po q 6 h as needed for chest pain.

Ibuprofen 600 mg po q 6 h as needed for pain

Alteplase 100 mg IV infusion over 2 h

Question 7 | Type: **Q**

What are the two most common vital sign abnormalities in patients with acute pulmonary embolism? (You may select more than one option.)

Fever

Hypotension

Hypoxia

Tachycardia

Tachypnea

Question 8 | Type: **Q**

Which of the following drugs is approved for outpatient treatment of pulmonary embolism? (You may select more than one option.)

Aspirin

Dabigatran

Enoxaparin

Fondaparinux

Question 9 | Type: **Q**

Which of the following is a component of the Wells Criteria for pulmonary embolism? (You may select more than one option.)

First-degree relative with history of pulmonary embolism

Heart rate > 100 beats/min

Hemoptysis

Immobilization for at least 3 days or history of surgery in the previous 4 weeks

© 2026 Rao AS et al. *JAMA Network Open*.

Case 2: Chest Pain in a 62-yr-old Man

Background

History of Present Illness:

A 62-yr-old man comes to his primary care clinic with the chief complaint of chest pain. He has no pain currently. He first noticed the pain 2 mo ago but attributed it to indigestion. He describes the pain as a pressure sensation in the middle of his chest that does not radiate anywhere else. It seems to come only with exertion and not at rest. He noticed it more last week when he had to shovel snow; he has had no pain since then. He says the pain usually lasts 10 to 15 min and goes away if he takes a break. When present, the pain is not worsened by movement or breathing. The last time he got the pain, he took an aspirin, which he thinks helped. The pain is not associated with shortness of breath, nausea, or diaphoresis. He denies any recent fever, cough, or infectious symptoms.

Review of Systems:

General: Patient has generally been feeling fine. Good energy level. Sleeping well.

Skin: No new rashes or skin lesions.

HEENT: No vision changes, ear pain, nasal symptoms, or sore throat.

Pulmonary: No dyspnea with exertion; no cough.

Cardiovascular: No palpitations, orthopnea, paroxysmal nocturnal dyspnea, or peripheral edema.

Gastrointestinal: No abdominal pain, nausea, or vomiting. Appetite is normal.

Genitourinary: Noncontributory

© 2026 Rao AS et al. *JAMA Network Open*.

Musculoskeletal: No recent injury or immobilization.

Neurologic: Noncontributory

Psychiatric: Noncontributory

Past Medical History:

Medical history: Hypertension, gastroesophageal reflux disease (GERD).

Surgical history: Left inguinal hernia repair at age 50.

Medications: Chlorthalidone 25 mg once/day, lisinopril 10 mg once/day, omeprazole 20 mg prn, sildenafil prn.

Allergies: No known drug allergies.

Family history: Father had myocardial infarction (MI) at age 43. Mother has hypertension and type 2 diabetes. No family history of sudden cardiac death.

Social history: Patient is married and works as an accountant. He does not participate in any formal exercise routine. Patient has smoked cigarettes for 25 yr; currently, he smokes 1 pack/day. He drinks 4 to 5 beers/wk. No illicit drug use.

Physical Examination:

General: appearance Middle-aged male in no apparent distress.

Vital signs:

Temperature: 37.2¬∞ C

Pulse: 76 beats/min

© 2026 Rao AS et al. *JAMA Network Open*.

BP: 162/86 mm Hg
Respirations: 14/min

Skin: No rashes or lesions.

HEENT: Oropharynx clear

Pulmonary: Lungs clear to auscultation bilaterally. No wheezing, rhonchi, or rales.

Cardiovascular: Regular rate and rhythm, normal S1 and S2. No murmurs, rubs, or gallops. Brisk carotid upstrokes ; all pulses 2+ and symmetrical bilaterally. No neck vein distention visible when patient is sitting at 45¬∞.

Gastrointestinal: Soft, nontender, nondistended abdomen; audible bowel sounds.

Genitourinary: Unremarkable

Musculoskeletal: Chest pain not reproducible with palpation. No lower-extremity edema, swelling, or tenderness.

Neurologic: Unremarkable

Mental: status Unremarkable

Questions

Question 1 | Type: DIFF

Based on the history and physical examination findings, which of the following differential diagnoses cannot be excluded at this time? (You may select more than one option.)

Acute coronary syndrome (ACS), unstable angina, or acute MI
Aortic dissection
Gastroesophageal reflux disease (GERD)
Musculoskeletal pain syndrome, including costochondritis
Myocarditis
Pericarditis

© 2026 Rao AS et al. *JAMA Network Open*.

Pneumonia

Pulmonary embolism (PE)

Severe aortic stenosis

Stable angina

Question 2 | Type: **DIAG**

Which of the following steps are essential to do before the patient leaves the clinic? (You may select more than one option.)

Chest x-ray

ECG

Pulse oximetry

Serum D-dimer level

Question 3 | Type: **DIAG**

🖼 **Image:** *2_DIAG_ECG*

Results of appropriate testing return and show that:

Oxygen saturation is 96% on room air.

ECG is obtained and is shown.

Which of the following is the correct interpretation of the ECG?

Acute anterolateral ischemia

Left bundle branch block

Left ventricular hypertrophy with secondary ST/T changes

Old inferior wall infarction

Question 4 | Type: **DIAG**

Given the previous findings, which of the following is the most appropriate step to guide diagnosis and treatment?

Cardiac catheterization

CT angiography of the chest

Echocardiography, transthoracic

Electron beam CT for calcium score

Serum troponin level

Treadmill stress test with ECG

Treadmill stress test with nuclear imaging

Question 5 | Type: **DX**

Results of treadmill stress test with nuclear imaging come back and show the following:

Large reversible perfusion defect in the territory of the left anterior descending (LAD) artery

Ejection fraction 60%

At this time, which of the following is the most likely diagnosis for this patient? (Please select only one option.)

Acute coronary syndrome

GERD

Musculoskeletal

Stable angina

Question 6 | Type: **MANG**

At this time, which of the following are the most appropriate next steps? (You may select more than one option.)

Admit to the hospital telemetry unit

Advise the patient to rest

Refer to a cardiothoracic surgeon for coronary artery bypass surgery (CABG)

Refer to a cardiologist for cardiac catheterization

Reinforce need to adhere to antihypertensive treatment

Serum lipid profile

Aspirin 81 mg once/day

Aspirin 325 mg once/day

Metoprolol 25 mg twice daily

Nitroglycerin 0.4 mg sublingual as needed for chest pain

Question 7 | Type: **Q**

© 2026 Rao AS et al. *JAMA Network Open*.

Which of the following chest pain characteristics, if present, suggest cardiac rather than noncardiac chest pain? (You may select more than one option.)

Lasting a few seconds

Pressure-like quality

Relieved by nitroglycerin

Relieved by rest

Sharp quality

Substernal location

Triggered by eating

Triggered by exertion

Triggered by palpation

Question 8 | Type: **Q**

Which of the following patient characteristics are relative contraindications to dobutamine stress echocardiography?

Able to exercise on a treadmill

Aortic stenosis, severe

Atrial fibrillation

Chronic obstructive pulmonary disease (COPD)

Hypertension, controlled

Hypertension, uncontrolled

Question 9 | Type: **Q**

Which of the following patient characteristics, if present in this patient, would have suggested unstable angina and therefore would have required a more urgent workup? (You may select more than one option.)

Pain increasing in duration

Pain increasing in frequency

Pain increasing in severity

Pain occurring at rest

Pain occurring more than 5 times/day

Right bundle branch block on ECG

Question 10 | Type: **Q**

© 2026 Rao AS et al. *JAMA Network Open*.

A 50-yr-old obese man with no other medical problems comes to the office because of concerns about his cardiovascular risk. The Pooled Cohort Risk Calculator (highly sensitive C-reactive protein [hsCRP]) indicates he is at intermediate risk of heart disease. He currently has no cardiac symptoms. Which of the following would be appropriate for further risk stratification of this patient?

Calcium score

Cardiac catheterization

Echocardiography

Stress ECG

© 2026 Rao AS et al. *JAMA Network Open*.

Case 3: Chronic Lethargy in a 68-Yr-Old Woman

Background

History of Present Illness:

A 68-yr-old woman comes to the office with her daughter because of a 4-mo history of gradually increasing lethargy and worsening memory. Her daughter is concerned because the mother has stopped remembering whether she has taken her regular medications. The daughter is worried that her mother might not be safe to live on her own. Prior to 4 mo ago, the woman had been very busy with community activities, which she has since stopped, and her daughter had not noticed any memory difficulties. The daughter is not aware of any recent illnesses or injuries.

Review of Systems:

General: The patient says she feels tired all the time and is usually cold even now during the summer. She has a poor appetite but has gained 5 kg in weight in the preceding 4 mo despite decreased appetite. No fever, chills, headache, or body aches.

Skin: Her skin has become dry, and her hair is now coarse and sparse. No rash or itching.

HEENT: She reports fullness in her throat, and her daughter has noticed that her mother‚Äôs voice has become hoarse and her face appears puffy, particularly around the eyes. No difficulty swallowing.

Pulmonary: The patient reports new exertional shortness of breath and needing to pause after climbing ¬Ω flight of stairs. No cough or wheezing.

Cardiovascular: She used to have occasional palpitations before but has had none for the last 2 mo. She denies chest pain, orthopnea, or paroxysmal nocturnal dyspnea.

Gastrointestinal: The patient‚Äôs bowel movements have decreased from daily to once every 3 to 4 days when using laxatives. She does not have any abdominal pain or vomiting. Her appetite is decreased.

Genitourinary: Noncontributory

Musculoskeletal: Her ankles and lower legs are more swollen than they used to be. She reports fatigue and leg weakness when climbing stairs or getting up from a chair.

© 2026 Rao AS et al. *JAMA Network Open*.

Neurologic: She has become slower at performing daily activities, and extra effort is required. Her daughter has noticed that her mother has trouble remembering recent events, such as whether she has taken her medications. She denies having focal weakness or sensory abnormalities, tremor, or visual symptoms.

Psychiatric: Her mood is depressed and she often stays in bed, not going out of the house for many days.

Past Medical History:

Medical history: Hypertension diagnosed 10 yr ago and controlled with three antihypertensives. Atrial fibrillation (AF) rate-controlled with bisoprolol; previous admission to hospital with a fast AF complicated with acute left ventricular failure; cardioversion failed to restore sinus rhythm.

Surgical history: Appendectomy at the age of 19 yr, left carpal tunnel syndrome surgery 2 yr ago.

Medications: Bisoprolol 5 mg once/day, ramipril 10 mg once/day, furosemide 40 mg once/day, warfarin 3 mg once/day.

Allergies: No known drug allergies.

Family history: Mother had hypothyroidism and a stroke in her 80s. Father died of metastatic colon cancer.

Social history: She eats a healthy balanced diet, and her only exercise is walking to nearby shops twice a week. She used to work as a receptionist but retired 3 yr ago. She is a nonsmoker and drinks alcohol only occasionally. She has not travelled abroad in the last 3 yr.

Physical Examination:

General appearance: The patient is wearing several layers of clothing including a wool sweater despite it being 89¬∞ F outside. She has a dull facial expression, and she looks pale and tired.

Vital signs:

© 2026 Rao AS et al. *JAMA Network Open*.

Temperature: 35.8¬∞ C

Pulse: 56 beats/min, irregular

BP: 120/90 mm Hg

Respirations: 16/min

Skin: Skin is cool and dry, no cyanosis or rash. Hair is coarse and dry with noticeable loss of hair from the eyebrows.

HEENT: Periorbital edema. Thyroid gland is slightly enlarged bilaterally, firm, and nontender. There is no cervical lymphadenopathy.

Pulmonary: Good air entry and exit with a few bibasilar fine end-inspiratory crackles and no wheeze.

Cardiovascular: Irregularly irregular heart rate of 56 beats/min, no murmurs, rubs or gallops. Jugular venous pulse visible but not elevated. There is 2+ edema of both lower extremities. Dorsalis pedis pulses are difficult to palpate due to leg edema.

Gastrointestinal: Previous appendectomy scar is visible. Abdomen is soft; there is a mild discomfort in the left iliac fossa with normal bowel sounds. Hard, brown, stool is present in the rectum on the rectal examination. Stool is negative for occult blood.

Genitourinary: Unremarkable

Musculoskeletal: A left wrist scar is present from surgical decompression of carpal tunnel syndrome. Tinel‚Äôs sign is positive on the right side.

Neurologic: Patient ‚Äôs movements and speech are slow but clear, no tremor. Cranial nerves II-XII intact. There is proximal weakness (4+/5) of both lower extremities, otherwise strength and muscle tone are normal and symmetrical; deep tendon reflexes are decreased generally with a prolonged relaxation phase. Plantar reflexes are down-going bilaterally.

Mental status: Patient reports a decreased mood but no suicidal thoughts.

Questions

Question 1 | Type: DIFF

© 2026 Rao AS et al. *JAMA Network Open*.

At this time, which of the following differential diagnoses should not be excluded? (You may select more than one option.)

Alzheimer disease

Anemia

Chronic bilateral subdural hematoma

Chronic kidney disease

Heart failure

Hyponatremia

Hypothyroidism

Parkinson disease

Stroke

Question 2 | Type: DIAG

At this time, it is most appropriate to order which of the following tests? (You may select more than one option.)

Serum electrolyte, BUN, and creatinine concentrations

Thyroid function tests

Urinalysis

Electrocardiogram (ECG)

Pulmonary function tests

Chest x-ray

CT scan of head

Complete blood count (CBC)

CT scan of the lungs with IV contrast

MRI of the head and neck with IV contrast

Question 3 | Type: DX

🖼 **Image:** *3_DX_ECG*

Results of appropriate testing return and show the following:

Thyroid-stimulating hormone (TSH): 88 microIU/mL

Thyroxine, Free (T4): 0.4 ng/dL

Triiodothyronine, free (T3): 0.8 pg/mL

Sodium: 135 mEq/L

Potassium: 4.3 mEq/L

© 2026 Rao AS et al. *JAMA Network Open*.

Urea nitrogen (BUN): 8 mg/dL

Creatinine: 0.9 mg/dL

Hemoglobin:9.8 g/dL

Leukocytes: 6,700/mcL

Platelets 159,000/mcL

Mean corpuscular volume (MCV) 88 fL

Mean corpuscular hemoglobin concentration (MCHC) 34 g/dL

Chest x-ray: Small volume fluid in the right interlobar fissure, mild bibasilar atelectasis, enlarged heart size.

Head CT: Normal

Results of ECG are shown.

A correct interpretation of this ECG is:

1st degree heart block

Atrial fibrillation

Mobitz II heart block

Sinus bradycardia

Question 4 | Type: **DIAG**

At this time what is the most likely diagnosis? (You may select only one option.)

Chronic fatigue syndrome

Chronic kidney disease

Chronic subdural hematoma

© 2026 Rao AS et al. *JAMA Network Open*.

Hyponatremia

Iron deficiency anemia

Hypothyroidism

Question 5 | Type: **DIAG**

At this time, the most appropriate next steps should include which of the following? (You may select more than one option.)

Pituitary hormone profile (serum LH, FSH, cortisol, prolactin levels)

Serum C-reactive protein (CRP) concentration

Serum thyroid peroxidase (TPO) antibodies

Thyroid ultrasound

Question 6 | Type: **DX**

Results of appropriate testing return and show the following:

Test (units)	Result
Serum thyroid peroxidase (TPO) antibodies	258 IU/mL

Given the results of recent testing, at this time what is the most likely diagnosis? (You may select only one option.)

Hashimoto thyroiditis

Hypothyroidism due to iodine deficiency

Euthyroid sick syndrome

Simple nontoxic goiter

Question 7 | Type: **MANG**

© 2026 Rao AS et al. *JAMA Network Open*.

A diagnosis of Hashimoto-thyroiditis is confirmed. At this time, management of this patient should include which of the following? (You may select more than one option.)

Liothyronine 25 mcg bid

L-thyroxine 25 mcg po once/day

L-thyroxine 75 mcg po once/day

L-thyroxine 150 mcg po once/day

Thyroid extract

Question 8 | Type: Q

Which of the following can cause primary hypothyroidism? (You may select more than one option.)

Craniopharyngioma

Drug-induced thyroiditis

Euthyroid sick syndrome

Hashimoto thyroiditis

Iodine deficiency

Pituitary macroadenoma

Postpartum thyroiditis

Radioiodine administration

Radiotherapy to the neck

Question 9 | Type: Q

© 2026 Rao AS et al. *JAMA Network Open*.

Which of the following conditions are associated with autoimmune hypothyroidism? (You may select more than one option.)

Addison's disease

Celiac disease

Gastrinoma

Pernicious anemia

Pheochromocytoma

Polyglandular autoimmune syndrome

Primary hyperparathyroidism

Vitiligo

Question 10 | Type: Q

Which medications require reassessment of thyroid function and may require an adjustment of the L-thyroxine dose? (You may select more than one option.)

Amiodarone

Bisoprolol

Carbamazepine

Ipilimumab

Interferon alpha

Lithium

Penicillin

© 2026 Rao AS et al. *JAMA Network Open*.

Phenytoin

Ramipril

Rifampin

Stavudine

Sunitinib

Question 11 | Type: **Q**

What are the possible complications of hypothyroidism? (You may select more than one option.)

Acute heart failure

Diabetes

Encephalopathy

Hypercalcemia

Hyperkalemia

Hyponatremia

Normocytic anemia

Megacolon

Myxedema coma

© 2026 Rao AS et al. *JAMA Network Open*.

Case 4: Confusion in a 74-Yr-Old Woman

Background

History of Present Illness:

A 74-yr-old woman is brought to the emergency department by her husband because of an episode of confusion. The day prior to her visit she attended a wedding at which she had "2 or 3" glasses of wine and afterwards felt extremely tired whereby she left early. When she got home, she went straight to bed without even undressing. She awoke about 3 h later and didn't remember going to bed or that she had just been at a wedding. Both arms were painful when she awoke, and she noticed blood in her mouth. Her husband says she had been "tossing around in bed." She returned to bed and awoke in the morning several hours past her usual time. She and her husband thought these events were very unusual, and although she felt back to normal except for some slight soreness in her arms, she decided to go the emergency department.

Review of Systems:

General: Has otherwise been feeling in her usual state of health. No fever, sweats, or weight change.

Skin: No recent skin rashes.

HEENT: Mild discomfort and swelling on left lateral aspect of tongue.

Pulmonary: No shortness of breath or cough.

Cardiovascular: No chest pain, palpitations or edema, no difficulty doing her usual activities.

Gastrointestinal: Chronic constipation which has been stable for years, no abdominal pain, nausea, or vomiting.

Genitourinary: No urinary frequency, hematuria, or dysuria. Episode of urinary incontinence during sleep the prior night. No episodes of urinary incontinence prior to the current episode.

Musculoskeletal: Mild, diffuse bilateral upper extremity discomfort.

Neurologic: No headache, no vision changes, no neck stiffness, dizziness, or vertigo. No weakness or loss of sensation.

© 2026 Rao AS et al. *JAMA Network Open*.

Psychiatric: Noncontributory

Past Medical History:

* Medical history: Chronic constipation; osteoporosis with multiple compression fractures in the lumbar spine.

*

Surgical history: Negative

*

Medications: Vitamin supplements

*

Allergies: None

*

Family history: Both parents lived to their 90s and had high blood pressure and dementia. Both died of pneumonia. She has no siblings.

*

Social history: She is a retired educator and lives at home with her husband. She does not smoke cigarettes or use illicit drugs. She occasionally drinks a glass of wine at dinner.

Physical Examination:

* General appearance: Thin elderly female who appears her stated age and in no apparent distress.

*

Vital signs:

* Temperature: 37¬∞ C
* Pulse: 78/min
* BP: 130/84 mm Hg
* Respirations: 14/min

*

Skin: No skin rashes or bruises.

*

HEENT: Dried blood in mouth with superficial tongue laceration of left lateral aspect.

© 2026 Rao AS et al. *JAMA Network Open*.

*

Pulmonary: Clear to auscultation bilaterally.

*

Cardiovascular: Regular rate and rhythm with no murmurs, rubs, or gallops.

*

Gastrointestinal: Soft, nontender and nondistended with normal bowel sounds.

*

Genitourinary: No costovertebral angle tenderness.

*

Musculoskeletal: Mild tenderness to palpation of bilateral upper extremities; no swelling, redness, or deformity. Intact range of motion.

*

Neurologic:

* Cranial nerves: 2 to 12 intact
* Motor exam: Muscle tone and strength in upper and lower extremities normal. No atrophy, no abnormal movements.
* Sensory exam: Intact to light touch, pinprick, temperature, vibration, and proprioception.
* Reflexes: Normal and symmetric throughout.
* Coordination: Normal
* Gait: Normal

*

Psychiatric: Alert and oriented x 3; affect: normal; mood: euthymic; memory: normal; judgment/insight: normal.

Questions

Question 1 | Type: DIFF

At this time, which of the following are common and/or dangerous differential diagnoses that cannot be excluded based on history and physical examination in this patient? (You may select more than one option.)

Acute stroke

Alcohol intoxication

Alcohol withdrawal

© 2026 Rao AS et al. *JAMA Network Open*.

Drug encephalopathy

Meningitis

Metabolic encephalopathy

Dementia

Subarachnoid hemorrhage (SAH)

Syncope

Subdural hematoma

Seizures

Question 2 | Type: **DIAG**

Given the previous findings, which of the following are the most appropriate steps to guide diagnosis and treatment? (You may select more than one option.)

CT scan of the brain without contrast

CT scan of the brain with contrast

Complete metabolic profile

Electrocardiogram (ECG)

Electroencephalography (EEG)

Erythrocyte sedimentation (ESR) level

Complete blood count with platelets and differential

Serum vitamin B12 level

© 2026 Rao AS et al. *JAMA Network Open*.

Serum alcohol level

Serum calcium level

Serum magnesium level

Urine toxicology screen

Question 3 | Type: **DIAG**

Results of appropriate testing return and show the following:

Serum:

Test (units)	Result
Sodium	133 mEq/L
Potassium	4.0 mEq/L
Chloride	102 mEq/L
Bicarbonate	22 mEq/L
BUN	12 mg/dL
Creatinine	0.8 mg/dL
Calcium level	9.0 mg/dL
Magnesium level	2.0 mEq/L
Glucose	70 mg/dL
ALT	10 U/L
AST	10 U/L
Albumin	4.0 g/dL
Alcohol level	10 mg/dL

Urine toxicology screen: Negative

ECG: Normal sinus rhythm, no acute changes

CT scan without contrast: No intracranial hemorrhage or mass effect; ill-defined rounded mass adjacent to the dura of the posterior falx.

Given the CT scan findings, which of the following is the most appropriate to guide diagnosis and treatment? (You may select more than one option.)

© 2026 Rao AS et al. *JAMA Network Open*.

Carotid ultrasound

EEG

EMG

Lumbar puncture

MRI with and without contrast of the head

Nerve conduction studies

Question 4 | Type: DX

Image: *4_DX_MRI*

Appropriate studies are obtained. EEG is reported as "Normal awake study. No epileptiform discharges seen." MRI of the head without contrast is shown:

MRI of the head without contrast is shown:

Which of the following is a correct interpretation of this patient's MRI? (You may select only one option.)

Normal

Meningioma

Acute stroke

Demyelinating disease

Brain abscess

© 2026 Rao AS et al. *JAMA Network Open*.

Subdural hematoma

Question 5 | Type: **DX**

The most likely diagnosis in this patient is? (You may select only one option.)

Cryptogenic seizures

Delirium tremens

Psychogenic seizures

Seizure due to hypoglycemia

Seizure due to hyponatremia

Seizure due to meningioma

Question 6 | Type: **MANG**

A diagnosis of seizure due to meningioma is confirmed.

At this time, which of the following are the most appropriate next steps? (You may select more than one option.)

Admit the patient to the hospital for surgical resection of the meningioma

Discharge the patient with a prescription for valproate 10mg/kg po once/day

Discharge the patient with follow-up with a neurologist if symptoms recur

Carbamazepine 200 mg po bid

Lorazepam 2 mg IV

Phenobarbital 2 mg/kg po once/day at bedtime

Phenytoin 4 mg/kg po once/day at bedtime

Question 7 | Type: **Q**

© 2026 Rao AS et al. *JAMA Network Open*.

Which of the following drugs have been reported to induce seizures? (You may select more than one option.)

Acetaminophen

Aminophylline

Ampicillin

Finasteride

Furosemide

Haloperidol

Mycophenalate mofetil

Penicillin

Prednisone

Propranolol

Pyridostigmine

Question 8 | Type: **Q**

Which of the following anticonvulsant drugs are most commonly associated with Stevens-Johnson syndrome in adults? (You may select more than one option.)

Gabapentin

Lamotrigine

Levetiracetam

Topiramate

© 2026 Rao AS et al. *JAMA Network Open*.

Valproate

Question 9 | Type: **Q**

What are some common side effects of anticonvulsant drugs? (You may select more than one option.)

Blindness

Diarrhea

Fatigue

Impotence

Insomnia

Nausea

Rash

Tinnitus

Vomiting

© 2026 Rao AS et al. *JAMA Network Open*.

Case 5: Cough in 47-Yr-Old Woman

Background

History of Present Illness:

A 47-yr-old woman comes to the office because of an 8-wk history of a cough. She says the cough is never productive, occurs at all times of the day (including at night), and is independent of activity. She is not sure whether she had an upper respiratory tract infection preceding the cough. She does recall that a new neighbor, who is a smoker, moved into a downstairs apartment at the time the cough began. The neighbor frequently smokes outside the patient's apartment window, triggering the cough.

She has not noticed any heartburn or runny nose. One month after the cough started, she noticed pain at the right side of her breastbone occurring with stretching and deep breathing. At that same time, she also noted that she has been wheezing during exertion. She has never had wheezing before. She has had no shortness of breath.

Review of systems:

General: Positive for 5- to 7-lb intentional weight loss via use of a FitBit¬Æ activity tracker. No fever, chills, or malaise. Appetite normal.

Skin: No rashes

HEENT: Feels congested at night. No vision or hearing changes.

Pulmonary: Cough and wheezing as per history of present illness.

Cardiovascular: Sharp, right-sided chest pain occurring with stretching, coughing, and deep breathing and lasting a second or two. No chest pain during exertion.

Gastrointestinal: Intermittent heartburn in the past but no recent episodes.

Genitourinary: Noncontributory

Musculoskeletal: Sternal pain as per history of present illness. No edema or pain in extremities.

Neurologic: Noncontributory

© 2026 Rao AS et al. *JAMA Network Open*.

Psychiatric: Noncontributory

Past Medical History

Medical history: Hypertension, hypothyroidism due to thyroidectomy 12 yr ago for a benign nodule, uterine cancer with hysterectomy 4 yr ago, and bipolar disorder. No known pulmonary or cardiac disease.

Surgical history: Thyroidectomy (12 yr ago), hysterectomy (4 yr ago).

Medications: Losartan, verapamil, levothyroxine, aripiprazole, bupropion, escitalopram, and topiramate.

Allergies: Intolerance to lurasidone (hand tremors) and oxycodone (nausea and vomiting).

Family history: No family history of lung cancer.

Social history: Life-long nonsmoker. Uses alcohol socially. Has never used illicit drugs. Works in a hospital business office. Divorced; no sexual contact for 8 yr. Lives in Maryland. Has not traveled outside the East Coast for 20 yr; has never traveled outside the US. No sick close contacts.

Physical Examination:

General appearance: Well-appearing woman; not in respiratory distress.

Vital signs:

Temperature: 36.8¬∞ C

Pulse: 79 beats/min

BP: 133/88 mm Hg

Respirations: 15/min

Pulse oximetry: 95% on room air

© 2026 Rao AS et al. *JAMA Network Open*.

Skin: No cyanosis, rashes, or other lesions.

HEENT: Anicteric sclerae, pupils equally round and reactive to light. No nasal polyps. Oropharynx clear, no postnasal drip. No lymphadenopathy.

Pulmonary: Normal respiratory effort. Good air entry and exit but bilateral wheezing noted during forced expiration. No crackles or rhonchi.

Cardiovascular: Normal rate, regular rhythm. No murmurs, rubs, or gallops. Jugular venous pressure not elevated.

Gastrointestinal: Soft, nontender, nondistended abdomen with normal bowel sounds.

Genitourinary: Not assessed

Musculoskeletal: Tender to palpation at the junction of the sternum and right 3rd and 4th ribs. No swelling, redness, or tenderness in the legs.

Neurologic: Normal gait with no focal deficits in strength or sensation.

Mental status: Appropriate. Alert and oriented.

Questions

Question 1 | Type: DIFF

At this time, which of the following differential diagnoses cannot be excluded based on history and physical examination in this patient? (You may select more than one option.)

Acute bronchitis

Asthma

Coronary ischemia

Fungal infection of lungs

© 2026 Rao AS et al. *JAMA Network Open*.

Gastroesophageal reflux disease (GERD)

Lung cancer

Medication-induced cough

Pneumonia

Postnasal drip

Postviral cough

Sarcoidosis

Tuberculosis (TB)

Question 2 | Type: **DIAG**

At this time, which of the following are the most appropriate next steps? (Please select only one choice.)

Ambulatory esophageal pH monitoring

Chest x-ray

CT of sinuses

ECG

Pulmonary function testing

Question 3 | Type: **MANG**

Results of pulmonary function testing return and show the following:

On room air:

- Normal forced vital capacity (FVC)
- Forced expiratory volume in 1 sec (FEV1): 60% of predicted

© 2026 Rao AS et al. *JAMA Network Open*.

- Peak expiratory flow (PEF): 65% of predicted

After administration of inhaled albuterol:

- FEV1: 80%
- PEF: 75%

Findings are consistent with reactive airway disease, possibly cough-variant asthma.

At this time, which of the following are appropriate management steps? (You may select more than one option.)

Albuterol inhaler 2 puffs q 6 h and prn for wheezing

Budesonide inhaler 1 puff bid

Combined ipratropium/albuterol inhaler 2 puffs q 6 h

Stop losartan

Ibuprofen 400 mg po q 6 h prn for chest pain

Omeprazole 20 mg po once/day

Prednisone 40 mg po once/day for 5 days

Oxycodone 5 mg/acetaminophen 325 mg tablet 1 to 2 po q 6 h prn for chest pain

Theophylline extended-release tablet 300 mg po q 12 h

Question 4 | Type: **Q**

Which of the following vaccines are particularly important for this patient to receive on a yearly basis? (You may select more than one option.)

Haemophilus influenza type b vaccine

Influenza vaccine

Pneumococcal polysaccharide vaccine (PPSV23)

© 2026 Rao AS et al. *JAMA Network Open*.

Pneumococcal conjugate vaccine (PCV13)

Question 5 | Type: **Q**

Which of the following are risk factors for tuberculosis? (You may select more than one option.)

Cigarette smoking

HIV infection

Liver cancer

Use of tumor necrosis factor-alpha inhibitors

Working in a day-care center

Question 6 | Type: **Q**

Nonpharmacologic management of this patient should also include which of the following? (Please select only one choice.)

Teaching the patient how to use a peak flow meter at home

Sleeping with a humidifier

Taking daily allergy medicine

Moving to a new apartment

© 2026 Rao AS et al. *JAMA Network Open*.

Case 6: Cough in a 2 yr-old-boy

Background

History of Present Illness:

A 2-yr-old boy is brought to the emergency department by his parents because of a 1-day history of cough and difficulty breathing. The parents state that the boy appeared normal until the previous night when he began coughing shortly after dinner. The cough stopped after about an hour; the child felt better and went to bed. In the morning, the cough returned and he began to have difficulty breathing, which the parents think is worsening. No one else at home is ill.

Review of Systems:

General: The child has no fever or malaise, and he was well until the cough began.

Skin: No rash is noted.

HEENT: He does not have sore throat, runny nose, ear pain, difficulty swallowing, or eye discharge or redness.

Pulmonary: The child has been coughing and is short of breath.

Cardiovascular: The child has not had chest pain.

Gastrointestinal: The child has not had abdominal pain, vomiting, or diarrhea. The parents say he did not want to eat breakfast because of the cough and dyspnea.

Genitourinary: Noncontributory

Musculoskeletal: Noncontributory

Neurologic: Noncontributory

Psychiatric: Noncontributory

© 2026 Rao AS et al. *JAMA Network Open*.

Past Medical History:

Medical history: Full-term, normal delivery. The boy has been healthy since birth except for two episodes of wheezing; each required a short course of bronchodilators but no daily treatment.

Surgical history: None

Medications: None

Allergies: None

Family history: Mother has mild, well-controlled asthma; father is healthy.

Social history: The child lives with his mother, father, and pet dog. The child‚Äôs vaccinations are up to date.

Physical Examination:

General appearance: Well-developed male child, awake and alert, who has obvious dry cough and mild respiratory distress. No cyanosis. He is able to speak with a normal-sounding voice.

Vital signs:

Temperature: 37¬∞ C

Pulse: 115 beats/min

BP: 100/75 mm Hg

Respirations: 30/min

Skin: Pink, warm, dry; no cyanosis or rash. Capillary refill normal.

HEENT: Tympanic membranes normal bilaterally. Nasal passages clear, no discharge. No drooling. No erythema or exudate in pharynx. Nontender, supple neck; no adenopathy.

© 2026 Rao AS et al. *JAMA Network Open*.

Pulmonary: Significant wheezing in the left lung that appears to be transmitted to the right lung. No rhonchi, rales, or stridor; no retractions or use of accessory muscles.

Cardiovascular: Mild tachycardia. S1 and S2 normal; no murmurs rubs or gallops.

Gastrointestinal: Abdomen soft and not tender or distended. Bowel sounds normal.

Genitourinary: Unremarkable

Musculoskeletal: Normal appearance and movement of extremities.

Neurologic: Awake and alert. Cranial nerves grossly intact. Normal motor and sensory exam.

Mental status: Responds appropriately for his age.

Questions

Question 1 | Type: DIFF

At this time, which of the following are common and/or dangerous differential diagnoses that cannot be excluded based on history and physical examination of this patient? (You may select more than one option.)

Allergic reaction

Asthma exacerbation

Bronchiolitis

Foreign body aspiration

Epiglottitis

Pertussis

Pneumonia

© 2026 Rao AS et al. *JAMA Network Open*.

Question 2 | Type: **DIAG**

At this time, which of the following are the most appropriate next steps? (You may select more than one option.)

Albuterol nebulizer

Basic metabolic panel

Chest x-ray: Anteroposterior(AP), lateral, and expiratory views

CT scan of chest

Complete blood count (CBC) with differential

Discharge home on cool-mist vaporizer

Heimlich maneuver

Pulse oximetry

Question 3 | Type: **MANG**

Results of appropriate testing return and show the following:

- Pulse oximetry: 96% on room air.
- Chest x-ray: Report states "AP and lateral views normal. The child is unable to cooperate with expiratory view."

At this time, which of the following is the most appropriate next step? (You may select more than one option.)

Admit the child for observation

Take a chest x-ray in the left and right lateral decubitus positions

Discharge on amoxicillin 45 mg/kg po q 8 h

Discharge on inhaled albuterol and prednisone 1 mg/kg po once/day

© 2026 Rao AS et al. *JAMA Network Open*.

Repeat attempt at chest x-ray expiratory view

Question 4 | Type: **DX**

🖼 **Image:** *6_DX_leftXRAY, 6_DX_rightXRAY*

Test results: Chest x-ray in the left and right lateral decubitus positions are taken and shown.

Left lateral decubitus view

Right lateral decubitus view

At this time what is the most likely diagnosis?

Asthma with air trapping

Foreign body, left lung

Foreign body, right lung

Pneumonia, right lung

Tension pneumothorax, left lung

Question 5 | Type: **MANG**

At this time, which of the following are the most appropriate treatment orders?

Consult ENT or pulmonary specialist for bronchoscopy

Sputum Gram stain and culture

Amoxicillin/clavulanate 45 mg/kg IV q 12 h

Back blows with child in an inverted position

Suction airway with a flexible catheter

Question 6 | Type: **Q**

© 2026 Rao AS et al. *JAMA Network Open*.

Which of the following are considered typical manifestations of acute bronchial foreign body in children? (You may select more than one option.)

Cough

Drooling

Dysphagia

Unilateral diminished breath sounds

Vomiting

Wheezing

Question 7 | Type: **Q**

Which of the following are typical complications of an unrecognized bronchial foreign body? (You may select more than one option.)

Atelectasis

Asthma

Bronchiectasis

Emphysema

Lung abscess

Lung cancer

Pneumonia

Question 8 | Type: **Q**

Which of the following is the most common location of foreign bodies aspirated by young children?

© 2026 Rao AS et al. *JAMA Network Open*.

Alveoli

Bronchi

Larynx

Trachea

Question 9 | Type: **Q**

In which bronchus is a foreign body most likely to be located in a child aged 1 yr to 3 yr old?

Right

Left

Either

© 2026 Rao AS et al. *JAMA Network Open*.

Case 7: Cough in a 54-yr-old man

Background

History of Present Illness:

A 54-yr-old man comes to the office because of a 3-day history of worsening cough that produces a moderate amount of yellowish, nonbloody sputum. He says the cough causes sharp pain in the right side of his chest, and the cough and pain have been causing difficulty sleeping. He has not had anything like this before.

Review of Systems:

General: Patient felt fine until about 4 days ago. He thinks he has had a fever but did not take his temperature. He had a shaking chill last night that shook the bed and alarmed his wife. He feels quite ‚Äúrun down‚Äù and was too sick to go to work today so he came in. Before onset of this illness, he felt fine and was doing all his normal activities. He has been eating normally with no recent weight loss.

Skin: Has not noticed rash or skin lesions.

HEENT: Runny nose and mild sore throat began about 4 days ago. Rhinorrhea is clear. He is able to swallow with only mild discomfort. He has not noticed swollen nodes in his neck.

Pulmonary: He has had dyspnea when climbing stairs since illness began but no dyspnea at rest. He feels vague, continuous ‚Äúdiscomfort‚Äù in the left side of his chest, and he has a sharp pain with inspiration. He denies wheezing.

Cardiovascular: There is no exertional chest pain, palpitations, orthopnea, or paroxysmal nocturnal dyspnea.

Gastrointestinal: He has had a poor appetite for several days but is able to eat and drink. He denies reflux symptoms.

Genitourinary: Noncontributory

Musculoskeletal: He has right knee pain with exertion. He has not noticed any pedal edema.

© 2026 Rao AS et al. *JAMA Network Open*.

Neurologic: Noncontributory

Psychiatric: Noncontributory

Past Medical History:

Medical history: Hypertension since age 42, well-controlled with medications. Hypercholesterolemia since age 50, poorly controlled with diet. No history of chronic obstructive pulmonary disease (COPD), asthma or other lung disease, coronary artery disease, renal disease, cancer, diabetes, or immunosuppressive disorders.

Surgical history: Tonsillectomy at age 10 yr; ligament repair of the right knee at age 26.

Medications:Chlorthalidone 25 mg once/day and enalapril 40 mg once/day for the past 6 yr.

Allergies: No known drug allergies. No seasonal allergies.

Family history: Mother is age 80 and has hypertension, type 2 diabetes, and high cholesterol. Father died at age 64 of coronary artery disease (CAD).

Social history: Patient is employed as a lawyer. He plays squash vigorously twice a week, is poorly compliant with a low-fat diet, and consumes about 6 to 8 alcoholic drinks/wk. He smoked 1 pack of cigarettes/day for 20 yr but quit smoking at age 44; he denies other drug use. His most recent travel was a Mediterranean cruise 8 mo ago; it included land excursions to Turkey, Greece, and Italy. No other recent travel. He has not had any history of recent immobilization. He has no known exposure to anyone with tuberculosis (TB) or other respiratory illness. He has not had unprotected sex with anyone other than his spouse for the past 3 yr. He has had annual flu vaccinations for the past 4 yr.

© 2026 Rao AS et al. *JAMA Network Open*.

Physical Examination:

General appearance: Tired-appearing, middle-aged male in no apparent respiratory distress.

Vital signs

Temperature: 38.5¬∞ C (101.3¬∞ F)

Pulse: 98 beats/min

BP: 128/74 mm Hg

Respirations: 20/min

Skin: Pink, warm, and slightly diaphoretic. No rash or cyanosis.

HEENT: Clear rhinorrhea visible from nares and in posterior pharynx; pharynx slightly injected. No cervical adenopathy.

Pulmonary: Breath sounds are equal bilaterally with good air entry and exit. Faint inspiratory crackles are present on the left side, heard more at the base. No wheezing is noted.

Cardiovascular: No jugular venous distention visible at 45¬∞. Heart regular; no murmurs, rubs or gallop. No peripheral edema.

Gastrointestinal: Unremarkable

Genitourinary: Unremarkable

© 2026 Rao AS et al. *JAMA Network Open*.

Musculoskeletal: No leg swelling, redness, or tenderness. No finger clubbing. There is a healed surgical scar on the right knee.

Neurologic: Unremarkable

Psychiatric: Alert; oriented to person, place, and time. Responds clearly and coherently to questions and commands.

Questions

Question 1 | Type: DIFF

At this time, which of the following differential diagnoses cannot be excluded based on history and physical examination in this patient? (You may select more than one option.)

ACE inhibitor-induced cough

Acute asthma

Acute bronchitis

COPD exacerbation

Lung cancer

Middle East respiratory syndrome

New-onset heart failure

Pneumonia

Postnasal drip

Pulmonary embolism (PE)

© 2026 Rao AS et al. *JAMA Network Open*.

TB

Question 2 | Type: **DIAG**

Given the patient's history and physical exam findings, which of the following steps are most appropriate for guiding diagnosis and treatment? (You may select more than one option.)

Arterial blood gasses (ABGs)

Blood and sputum cultures

Complete blood count (CBC)

Chest CT scan

Chest x-ray

ECG

Pulse oximetry

Serum chemistry profile

Serum D-dimer level

Serum troponin level

Question 3 | Type: **DX**

🖼 **Image:** *7_DX_XRAY*

Results of appropriate testing return and show the following:

- Pulse oximetry on room air: 94%

Chest x-ray is obtained and is shown.

Which of the following is the correct interpretation of the chest x-ray? (Please select only one option.)

© 2026 Rao AS et al. *JAMA Network Open*.

Diffuse bilateral interstitial infiltrates

Hilar adenopathy

Left lower lobe infiltrate

Normal film

Question 4 | Type: **DX**

At this time what is the most likely diagnosis? (Please select only one option.)

Acute bronchitis

Community-acquired pneumonia

Health care-associated pneumonia

Lung cancer

Question 5 | Type: **MANG**

A diagnosis of community-acquired pneumonia is confirmed.

At this time, which of the following are the most appropriate treatment orders? (You may select more than one option.)

Schedule follow-up visit in 6 wk for repeat chest x- ray

Oxygen 4 L/min by nasal cannula

Chest physical therapy twice a day

Albuterol inhaler, 2 puffs qid and as needed

© 2026 Rao AS et al. *JAMA Network Open*.

Azithromycin 500 mg po once/day, then 250 mg po once/day

Cefotaxime 1 g IV q 8 h

Discontinue enalapril

Guaifenesin/dextromethorphan syrup qid

Question 6 | Type: **Q**

Which organisms are the most likely cause of community-acquired pneumonia in this patient? (You may select more than one option.)

Blastomyces dermatitidis

Chlamydia pneumoniae

Haemophilus influenzae

Hantavirus

Legionella sp

Mycoplasma pneumoniae

Pneumocystis jirovecii

Pseudomonas aeruginosa

Streptococcus pneumonia

Question 7 | Type: **Q**

Which of the following factors, if present, would increase the patient's pre-test probability of pulmonary embolism (PE)? (You may select more than one option.)

Decreased breath sounds

Exertional dyspnea

© 2026 Rao AS et al. *JAMA Network Open*.

Fever

Heart rate: 110 beats/min

History of cancer

Pleuritic chest pain

Recent immobilization for 3 days

Visible leg swelling

Question 8 | Type: **Q**

Which of the following factors, if present, would suggest increased risk of a poor outcome in patients with community-acquired pneumonia? (You may select more than one option.)

Altered mental status

Bilateral wheezing

Heart rate: 125 beats/min

History of liver disease

Oxygen saturation: 92%

Previous history of pneumonia

Respirations: 24/min

Temperature: 40º C

© 2026 Rao AS et al. *JAMA Network Open*.

Case 8: Dyspnea and Back Pain in 24-yr-old man

Background

History of Present Illness:

A 24-yr-old man comes to the emergency department (ED) because of sudden onset of shortness of breath and back pain. Yesterday, the patient was completing a 2-mile run, which he does 2 times/wk, when he suddenly experienced shortness of breath and sharp pain in his spine and on the right side of his back. The pain is constant, mild, and nonradiating and does not worsen during movement. His shortness of breath continued throughout the night and caused difficulty sleeping. The shortness of breath is worse this morning, prompting him to come to the ED.

Review of Systems:

General: Has been feeling well and doing his normal activities until symptoms began yesterday. No fever, chills, or malaise.

Skin: Noncontributory

HEENT: Reports frequent runny nose.

Pulmonary: Shortness of breath that is present at rest and worsens during exertion. No cough or wheezing.

Cardiovascular: No chest pain or palpitations.

Gastrointestinal: Noncontributory

Genitourinary: Noncontributory

Musculoskeletal:Back pain as per his history of present illness.

Neurologic: No numbness or weakness of extremities.

Psychiatric: Noncontributory

© 2026 Rao AS et al. *JAMA Network Open*.

Past Medical History

Medical history: None; sees a physician regularly for routine physicals. No history of similar illness or symptoms.

Surgical history: None

Medications: None

Allergies: None

Family history: Both parents are alive; father has hypertension; mother is well. Maternal grandfather may have died of lung cancer. No known coronary artery disease in family.

Social history: Lives with his parents and works full-time in a grocery store; does occasional light lifting without difficulty. Smokes 7 to 10 cigarettes/day, which is decreased from 1 pack/day. Drinks alcohol once/wk to once/mo. Used marijuana in his teens.

Physical Examination:

General appearance: Well-appearing, normal body habitus, and in no distress.

Vital signs:

Temperature: 36.2¬∞ C

Pulse: 79 beats/min

Respirations: 18/min

BP: 122/72 mm Hg

© 2026 Rao AS et al. *JAMA Network Open*.

Pulse oximetry: 94% on room air

Skin: No cyanosis, diaphoresis, rash, or other lesions.

HEENT: Anicteric sclerae, pupils equally round and reactive to light. Oropharynx clear.

Pulmonary: Normal respiratory effort. Decreased breath sounds on the right side.

Cardiovascular: Normal rate, regular rhythm. No murmurs, rubs, or gallops. Jugular venous pressure is not elevated. Pulses normal and symmetric in upper and lower extremities.

Gastrointestinal: Soft, nontender, nondistended abdomen; normal bowel sounds.

Genitourinary: Not assessed

Musculoskeletal: Chest, back, and spine nontender; no palpable crepitance. Extremities appear normal; no leg swelling, erythema, or tenderness.

Neurologic: Upper and lower extremities have normal, symmetric strength, sensation, and deep tendon reflexes in upper and lower extremities. Gait normal.

Mental status: Appropriate. Alert and oriented.

Questions

Question 1 | Type: DIFF

At this time, which of the following differential diagnoses cannot be excluded based on history and physical examination in this patient? (You may select more than one option.)

Asthma exacerbation

Lung cancer

Musculoskeletal back pain

Myocardial infarction (MI)

© 2026 Rao AS et al. *JAMA Network Open*.

Pericarditis

Pneumonia

Pulmonary embolism

Spontaneous pneumothorax

Tension pneumothorax

Thoracic aortic dissection

Question 2 | Type: **DIAG**

Given the patient's current history and examination findings, which of the following steps are most appropriate to do at this time? (You may select more than one option.)

Chest x ray

CT angiography (CTA) of the chest

D-dimer testing

ECG

Oxygen 6 L by nasal cannula

Thoracic spine x-rays

Question 3 | Type: **DX**

🖼 **Image:** *8_DX_XRAY*

Which of the following is the correct interpretation of this chest x-ray?

Calcified right hilar mass

© 2026 Rao AS et al. *JAMA Network Open*.

Fracture of the right 7th rib

Pneumothorax on the right side

Tension pneumothorax on the right side

Normal chest x-ray

Question 4 | Type: **MANG**

Which of the following steps are essential to do immediately ? (You may select more than one option.)

Admit the patient to the hospital

Administer azithromycin 500 mg po

Continue oxygen 6 L by nasal cannula

CT scan of the chest without IV contrast

CT scan of the chest with IV contrast

Discharge the patient and schedule follow-up chest x-ray in 1 day

Do immediate needle decompression

Increase supplemental oxygen to 100% via nonrebreathing face mask

Place a 20 French chest tube on the right side

Place a 36 French chest tube on the right side

© 2026 Rao AS et al. *JAMA Network Open*.

Schedule video-assisted thoracoscopic surgery and chemical pleurodesis

Question 5 | Type: **Q**

What is the percentage of pneumothorax patients who have primary spontaneous pneumothorax?

5 %

20 %

50 %

75 %

Question 6 | Type: **Q**

What is the peak age of patients who develop primary spontaneous pneumothorax?

20s

40s

60s

80s

Question 7 | Type: **Q**

Which of the following are risk factors for primary spontaneous pneumothorax? (You may choose more than one option.)

Male sex

Smoking

Family history

Short stature

© 2026 Rao AS et al. *JAMA Network Open*.

© 2026 Rao AS et al. *JAMA Network Open*.

Case 9: Exertional dyspnea in a 76 yr-old-man

Background

History of Present Illness:

A 76-year-old man comes to the office because of shortness of breath. He is presently being managed for end-stage renal disease on hemodialysis. Today, the patient states that over the past 6 months he has noted increasing difficulty breathing when he exerts himself. He is relatively sedentary but does climb a flight of stairs to his bedroom at night; he says he used to be able to do this without any problem, but now he reports he needs to stop halfway up the stairs to rest. Occasionally he has mild substernal chest pressure along with the shortness of breath. The chest pressure does not radiate and is not accompanied by nausea or diaphoresis. He has noted increased swelling in his legs. He denies any shortness of breath at rest, orthopnea, or paroxysmal nocturnal dyspnea. He denies any episodes of palpitations, lightheadedness, or syncope. He denies any cough, fever, chills, or night sweats.

Review of Systems:

General: Patient generally feels fatigued. No recent weight changes.

Skin: No rashes or skin lesions.

HEENT: No vision changes, ear pain, nasal congestion or discharge, or sore throat.

Pulmonary: See HPI

Cardiovascular: See HPI

Gastrointestinal: Good appetite. No abdominal pain, nausea, vomiting, or change in bowel patterns.

Genitourinary: Has not produced urine for several years since beginning dialysis. Has not missed any dialysis treatments.

Musculoskeletal: No redness or pain in extremities. He has noticed increased swelling of his feet and ankles in recent months.

© 2026 Rao AS et al. *JAMA Network Open*.

Neurologic: Noncontributory

Psychiatric: Noncontributory

Past Medical History:

Medical history: Hypertension, hyperlipidemia, type 2 diabetes mellitus, end-stage renal disease on hemodialysis.

Surgical history: Left knee replacement at age 65 years.

Medications: Lisinopril 10 mg orally daily, atorvastatin 40 mg orally daily, insulin glargine 50 U subcutaneously daily, calcium carbonate 500 mg orally twice a day with meals, darbepoietin 40 mcg IV weekly.

Allergies: No known drug allergies.

Family history: Father had hypertension and stroke. Mother had type 2 diabetes mellitus.

Social history: Patient is married and is a retired teacher. He has never smoked and does not use alcohol or illicit drugs.

Physical Examination:

General appearance: Thin, elderly male in no apparent distress.

Vital signs:

© 2026 Rao AS et al. *JAMA Network Open*.

Temperature: 37.2¬∞ C
Pulse: 72 beats/min
Blood Pressure: 155/86 mm Hg
Respirations: 16/min

Skin: No rashes or cyanosis

HEENT: Nares patent, no discharge; oropharynx clear.

Neck: Jugular venous pressure 5 cm above the clavicle at 45 degrees. Delayed carotid upstrokes.

Pulmonary: Lungs are clear to auscultation bilaterally. No wheezes, rhonchi, or rales. No dullness to percussion.

Cardiovascular: Regular rate and rhythm, normal S1, diminished S2, crescendo/decrescendo systolic murmur heard best at the right upper sternal border radiating to the carotids. No rubs or gallops.

Gastrointestinal: Soft, nontender, nondistended. Normal bowel sounds are present in all quadrants. Rectal exam: Nontender, stool heme negative.

Genitourinary: Unremarkable

Extremities: 1+ pitting edema to mid-shin bilaterally, no tenderness or erythema. 1+ dorsalis pedis pulses bilaterally. Dialysis graft present on right forearm with good thrill.

Musculoskeletal: Unremarkable

Neurologic: Unremarkable

Mental status: Unremarkable

Questions

Question 1 | Type: DIFF

© 2026 Rao AS et al. *JAMA Network Open*.

At this time, which of the following are common and/or dangerous differential diagnoses that cannot be excluded based on history and physical examination in this patient? (You may select more than one option.)

Acute decompensated heart failure (ADHF)

Anemia

Aortic stenosis

Atrial fibrillation

Chronic histoplasmosis

Chronic obstructive pulmonary disease (COPD)

Coronary artery disease (CAD)

Mitral regurgitation

Pneumonia

Pulmonary embolism (PE)

Volume overload from end-stage renal disease

Tuberculosis

Question 2 | Type: DIAG

Given the previous findings, which of the following are the most appropriate next steps to guide diagnosis and treatment? (You may select more than one option.)

Chest CT scan

Chest x-ray (CXR)

© 2026 Rao AS et al. *JAMA Network Open*.

Complete blood count (CBC)

Electrocardiogram (ECG)

Serum brain natriuretic peptide (BNP) level

Serum troponin I level

Transesophageal echocardiography (TEE)

Transthoracic echocardiography (TTE)

Question 3 | Type: **DX**

🖼 **Image:** *9_DX_ECG*

Results of appropriate testing return and are shown:

Serum:

Test (units)	Result
BNP level	465 pg/mL
Troponin	0.05 ng/mL

CBC:

Test (units)	Result
Hemoglobin	9 g/dL
WBC count	5,300/mcL
Platelet count	220,000/mcL

Chest x-ray: Normal heart size, mild costophrenic angle blunting, lung parenchyma is clear.

Transthoracic echocardiography: Normal left ventricular size. Left ventricular ejection fraction 60%. Normal right ventricular size and function. No regional wall motion abnormalities. Moderate concentric left ventricular hypertrophy. Normal diastolic function. Severe calcific aortic stenosis (mean gradient 45 mm Hg, AVA 0.85 cm2).

Results of ECG are shown:

Which of the following is a correct interpretation of the ECG? (Please select only one option.)

© 2026 Rao AS et al. *JAMA Network Open*.

Normal sinus rhythm with acute anterior ischemia

Normal sinus rhythm with left ventricular hypertrophy

Normal sinus rhythm with nonspecific ST-T wave changes

Sinus arrhythmia with left bundle branch block

Question 4 | Type: **DX**

At this time, which of the following is the most likely diagnoses? (Please select only one option.)

Acute coronary artery ischemia

Acute decompensated heart failure (ADHF)

Severe aortic stenosis

Volume overload due to end-stage renal disease

Question 5 | Type: **MANG**

At this time, a diagnosis of severe aortic stenosis is confirmed. Which of the following is the most appropriate management? (You may select more than one option.)

Admit to the hospital for emergency aortic valve surgery

Follow up in 6 months for repeat echocardiography

Refer to cardiology to evaluate for surgery

Exercise stress test

Pharmacologic stress test

© 2026 Rao AS et al. *JAMA Network Open*.

Furosemide 20 mg orally twice a day

Metoprolol succinate 25 mg orally once a day

Nitroglycerin 0.4 mg sublingual as needed for chest pain

Question 6 | Type: **Q**

Which of the following physical examination findings suggest severe aortic stenosis? (You may select more than one option.)

Delayed carotid upstrokes

Diminished or absent S2

Displaced point of maximal impulse (PMI)

Early peaking systolic murmur

Increase in murmur intensity with inspiration

Late peaking systolic murmur

Loud S2

Murmur that radiates to the carotid arteries

Question 7 | Type: **Q**

Which of the following patients meet criteria for aortic valve replacement? (You may select more than one option.)

52-year-old man with history of severe COPD and moderate aortic stenosis with new dyspnea on exertion

64-year-old asymptomatic woman with severe aortic stenosis and left ventricular ejection fraction (LVEF) of 40%

© 2026 Rao AS et al. *JAMA Network Open*.

72-year-old asymptomatic woman with moderate aortic stenosis and left ventricular ejection fraction (LVEF) of 65%

75-year-old man with 3-month history of exertional chest pressure and severe aortic stenosis on TTE

© 2026 Rao AS et al. *JAMA Network Open*.

Case 10: Fatigue and palpitations in a 53-year-old woman

Background

History of Present Illness:

A 53-yr-old woman comes to the office because of palpitations. She is a current patient there and has a history of well-controlled hypertension, type 2 diabetes, and tension headaches. She states that she has noticed intermittent palpitations over the past 2 mo. She describes the episodes as a sensation of irregular and racing heartbeats that are accompanied by fatigue. She denies any chest pain, shortness of breath, light-headedness, or syncope. The episodes last from 10 min to several hours, occur once or twice/wk, and resolve on their own. The last episode was 3 days ago. She cannot identify any particular triggers. She is generally active and walks 3 miles several days/wk without any exertional symptoms including chest pain and dyspnea. She has no family history of early cardiac disease or sudden death. She denies any recent fever or infectious symptoms.

Review of Systems:

General: Patient has generally been feeling fine.

Skin: No new rashes or skin lesions.

HEENT: No vision changes, ear pain, nasal symptoms, or sore throat.

Pulmonary: Denies shortness of breath, dyspnea during exertion, and cough.

Cardiovascular: No orthopnea, paroxysmal nocturnal dyspnea, or lower-extremity edema.

Gastrointestinal: Denies abdominal pain and poor appetite.

© 2026 Rao AS et al. *JAMA Network Open*.

Genitourinary: Noncontributory

Reproductive: Noncontributory

Musculoskeletal: No recent injury.

Neurologic: Noncontributory

Psychiatric: Has been generally in good spirits; no extremes of mood.

Past Medical History:

Medical history: Hypertension, type 2 diabetes, and tension headaches.

Surgical history: Appendectomy at age 20.

Medications: Lisinopril 10 mg daily and metformin 500 mg bid.

Allergies: No known drug allergies.

Family history: Mother is alive and has hypertension and diabetes mellitus. Father died of lung cancer. No history of early coronary artery disease or sudden cardiac death.

Social history: Patient is married and works as a school teacher. Regularly exercises 3 times/wk. No history of smoking. Drinks a glass of wine with dinner most nights and one cup of coffee each morning. Denies use of decongestants, cocaine, and other stimulants. No illicit drug use.

© 2026 Rao AS et al. *JAMA Network Open*.

Physical Examination:

General appearance: Slender middle-aged woman in no apparent distress.

Vital signs:

Temperature: 37.2¬∞ C

Pulse: 58 beats/min

BP: 125/74 mm Hg

Respirations: 14/min

Oxygen saturation: 96% on room air

Skin: No rashes noted.

HEENT: Oropharynx clear.

Pulmonary: Lungs clear to auscultation bilaterally. No wheeze, rhonchi, or rales.

Cardiovascular: Regular rate and rhythm, normal S1 and S2; no murmurs, rubs, or gallops. Brisk carotid upstrokes. No jugular venous distention.

Gastrointestinal: Soft, nontender, nondistended abdomen; positive bowel sounds.

Genitourinary: Unremarkable

Musculoskeletal: Unremarkable

Neurologic: Unremarkable

Mental status: Unremarkable

Questions

Question 1 | Type: DIFF

At this time, which of the following differential diagnoses cannot be excluded based on history and physical examination in this patient? (You may select more than one option.)

© 2026 Rao AS et al. *JAMA Network Open*.

Anemia

Anxiety or panic attack

Atrial fibrillation

Atrial flutter

Atrioventricular nodal reentrant tachycardia (AVNRT)

Hypoglycemia

Medication or substance use

Nonsustained ventricular tachycardia (NSVT)

Premature atrial complexes (PACs)

Premature ventricular complexes (PVCs)

Sustained ventricular arrhythmias

Question 2 | Type: **MANG**

Which of the following steps are essential to do immediately? (You may select more than one option.)

ECG

Event monitor

24-h Holter monitor

Cardiac enzymes

Stress test

© 2026 Rao AS et al. *JAMA Network Open*.

Admit to monitored hospital bed

Question 3 | Type: **DX**

🖼 **Image:** *10_DX_ECG*

Which of the following is a correct interpretation of this ECG? (You may choose only one option.)

Sinus arrhythmia

Sinus bradycardia at 58 beats/min

Sinus bradycardia at 50 beats/min

Sinus rhythm with early repolarization

Wandering atrial pacemaker

Question 4 | Type: **DX**

🖼 **Image:** *10_DX_ECG2*

The patient is put on cardiac event monitoring. On day 18 of monitoring, the patient develops the rhythm shown in strip below.

The patient is contacted and reports worsening symptoms of palpitations as well as new symptoms of light-headedness and dyspnea at rest.

At this time what is the most likely diagnosis? (You may choose only one option.)

Atrial fibrillation

Atrial flutter

Atrioventricular nodal reentry tachycardia (AVNRT)

© 2026 Rao AS et al. *JAMA Network Open*.

Junctional tachycardia

Multifocal atrial tachycardia(MAT)

Premature atrial complexes (PACs)

Question 5 | Type: **MANG**

Which of the following steps are essential to do immediately? (You may select more than one option.)

Instruct the patient to go to the emergency department right away.

Instruct the patient to double up on her lisinopril dosage, and call if her symptoms do not lessen.

Instruct the patient to perform a Valsalva maneuver (such as bearing down hard as if she is having a bowel movement).

Instruct the patient to take aspirin 325 mg immediately, and come to the clinic the next day.

Question 6 | Type: **MANG**

The patient comes to the emergency department (ED) 3 days later, despite having been asked to seek immediate medical evaluation. Now when evaluated in the ED, she has an irregular heart rate at 138 beats/min, BP 140/80 mm Hg, and respirations 12/min. She currently has palpitations, light-headedness, and fatigue, but she denies any new symptoms or changes to her medical history. ECG in the ED now confirms persistent atrial fibrillation.

Which of the following steps are essential to do immediately? (You may select more than one option.)

Aspirin 325 mg daily

Adenosine 6 mg IV push

Diltiazem 15 mg by IV push followed by diltiazem drip at 5 mg/h

Digoxin 0.25 mg by IV push

© 2026 Rao AS et al. *JAMA Network Open*.

Heparin 60 U/kg IV followed by 12 mg/kg/h drip

CBC

Complete metabolic panel

Serum PT/PTT

Serum thyroid-stimulating hormone (TSH) level

Cardiac catheterization

Immediate synchronized cardioversion

Transesophageal echocardiography (TEE)

Transthoracic echocardiography (TTE)

Question 7 | Type: **Q**

Which of the following characteristics are used to assess stroke risk in patients with atrial fibrillation? (You may select more than one option.)

Age

Sex

History of heart failure

Hypercholesterolemia

Hypertension

Peripheral vascular disease

© 2026 Rao AS et al. *JAMA Network Open*.

Renal function

Smoking

Type 2 diabetes mellitus

Stroke/transient ischemic attack

Question 8 | Type: **Q**

Which of the following are a contraindication to the use of direct oral anticoagulant drugs in patients with atrial fibrillation?

Age >65

Chronic obstructive pulmonary disease (COPD)

Severe mitral stenosis

Renal dysfunction with creatinine clearance of 50 mL/min

Question 9 | Type: **Q**

Which of the following is a contraindication to anticoagulation therapy? (You may select more than one option.)

Bioprosthetic heart valve

CHA_2DS_2-VASc score of 0

Frequent nosebleeds

History of gastroesophageal reflux disease

Intracranial hemorrhage

© 2026 Rao AS et al. *JAMA Network Open*.

Case 11: Hypernasality and Ptosis in a 48-yr-old-woman

Background

History of Present Illness:

A 48-yr-old woman comes to the office because of a nasal-sounding voice. She has been treated in the past for chronic sinusitis and asthma. A year ago, she had balloon rhinoplasty to enlarge her sinuses. Today, she says that she first noticed the nasal-sounding voice a week after surgery, but it has become more apparent over the last 3 mo.

Today, she says it feels as though air escapes through her nose while speaking, giving it a nasal quality. Her voice is better in the morning and worse later in the day. She says she does not have dysphagia but notes occasional nasal regurgitation of liquids but not food. She denies diplopia, drooping eyelids, loss of smell or taste, and difficulty with balance or gait. She denies having arm and leg weakness and numbness.

Review of Systems:

General: She has generally felt well and has been able to do her usual activities. Since her sinus surgery, she has had occasional shooting pains in the back of her head on the left occiput.

Skin: Noncontributory

HEENT: No nasal congestion or discharge.

Pulmonary: No dyspnea or wheezing.

Cardiovascular: No chest pain or palpitations.

Gastrointestinal: Good appetite, normal bowel movements, and no recent weight loss or gain.

Genitourinary: No difficulty starting or stopping urination and no incontinence. Last normal menstrual period 3 wk ago.

Musculoskeletal: No extremity or joint pain. Normal perceived strength.

© 2026 Rao AS et al. *JAMA Network Open*.

Neurologic: See previous screen (History of Present Illness).

Psychiatric: Normal mood, although she is beginning to be concerned about her vocal symptoms.

Past Medical History:

Medical history: Asthma controlled by daily corticosteroid inhaler. Uses rescue bronchodilator 3 to 4 times/yr; last use was 2 mo ago. Sinusitis, with multiple episodes in the past, but none since her sinus surgery.

Surgical history: Balloon rhinoplasty for sinus enlargement (1 yr ago).

Medications: Beclomethasone inhaler twice/day; albuterol inhaler as needed.

Allergies: Erythromycin

Family history: Negative

Social history: She eats a regular diet and does not exercise. There is no history of recent travel. She denies drug, alcohol, and tobacco use. Immunizations are up to date, and there has been no recent exposure to illness.

Physical Examination:

General appearance: Normal-appearing woman who looks well for her age. Her voice has a distinct nasal quality.

Vital signs:

Temperature: 37¬∞ C

Pulse: 68 beats/min

BP: 110/76 mm Hg

© 2026 Rao AS et al. *JAMA Network Open*.

Respirations: 14/min

Skin: No rash or lesions

HEENT: Nasal passages patent bilaterally; no discharge, lesions, or polyps. Pharynx normal. No cervical adenopathy.

Pulmonary: Good air entry and exit. No rhonchi, rales, or wheezes.

Cardiovascular: Regular rhythm and rate; no murmurs, rubs, or gallop.

Gastrointestinal: No tenderness or masses

Genitourinary: Unremarkable; pelvic examination deferred.

Musculoskeletal: Unremarkable

Neurologic:

Cranial nerves: 2 to 12 grossly intact, although there appears to be minimal ptosis on the left with sustained upward gaze

Motor: Muscle tone in upper and lower extremities is normal and symmetric; there is no atrophy or abnormal movement.

Strength: 5/5 in all four extremities; no fatigue at deltoids with repetitive testing

Sensation: Intact to light touch, sharp pain, temperature, and vibration and symmetric bilaterally; sensation to joint position intact

Reflexes: Normal and symmetric

Coordination: Normal

Gait and balance: Normal; negative Romberg test

Mental status: Alert, oriented x 3. Normal affect. Euthymic mood. Normal memory. Normal judgment and insight.

Questions

Question 1 | Type: DIFF

At this time, which of the following differential diagnoses cannot be excluded based on history and physical examination in this patient? (You may select more than one option.)

© 2026 Rao AS et al. *JAMA Network Open*.

An adverse effect of inhaled corticosteroids

Amyotrophic lateral sclerosis (ALS)

Botulism

Chronic rhinosinusitis

Eaton-Lambert syndrome

Myasthenia gravis

Myotonic muscular dystrophy

Mitochondrial myopathy

Oculopharyngeal muscular dystrophy

Sinus cancer

Question 2 | Type: DIAG

Given the previous findings, which of the following steps are now most appropriate for guiding diagnosis and treatment? (You may select more than one option.)

Carotid Doppler ultrasonography

© 2026 Rao AS et al. *JAMA Network Open*.

Edrophonium test

EEG

EMG with repetitive nerve testing

Muscle biopsy

MRI of the brain

Serum acetylcholine receptor (AChR) antibody

Serum creatine kinase

Question 3 | Type: **DX**

Results of appropriate testing return and show the following:

EMG with repetitive nerve testing shows decremental response to 3-Hz stimulation in the left median nerve recording from the abductor pollicis brevis muscle and in the accessory nerve recording from the trapezius. The decrement is less pronounced after 10 sec of exercise (facilitation) and is more pronounced after a further 60 sec of exercise (postfacilitation exhaustion).

Serum:

Test (units)

Result

Serum acetylcholine receptor antibody titer is 0.28 nmol/L

Normal reference range < 0.02 nmol/L

Serum creatine kinase is 50 U/L [833 nkat/L]

© 2026 Rao AS et al. *JAMA Network Open*.

Normal reference range 30-170 U/L

At this time what is the most likely diagnosis?

Mitochondrial myopathy

Myasthenia gravis

Myotonic muscular dystrophy

Oculopharyngeal muscular dystrophy

Question 4 | Type: **MANG**

Given the previous findings, which of the following steps are now most appropriate for guiding diagnosis and treatment? (You may select more than one option.)

Admit the patient for plasma exchange

Admit the patient for IV immunoglobulin (IVIG) treatment

Prednisone 20 mg po once/ day

Prednisone 1 mg/ kg po once/ day

Pyridostigmine 60 mg po q 4-6 h

Azathioprine 50 mg po once/ day

© 2026 Rao AS et al. *JAMA Network Open*.

MRI of the chest

X-ray of the chest

Question 5 | Type: **Q**

🖼 **Image:** *11_Q_leftMRI, 11_Q_rightMRI*

Please review the result by clicking on each image.

What is the most likely cause of the anterior mediastinal mass shown in these images? (You may select more than one option.)

Cardiac sarcoma

Lymphoma

Teratoma

Thymoma

Thyroid carcinoma

Question 6 | Type: **Q**

Which of the following drugs should be avoided in patients with myasthenia gravis? (You may select more than one option.)

Botox injections

Clindamycin

© 2026 Rao AS et al. *JAMA Network Open*.

Levoquin

Hydrochlorothiazide

Ramipril

Vancomycin

Question 7 | Type: **Q**

Which of the following autoimmune diseases can be mistaken for ocular myasthenia? (You may select more than one option.)

Addison disease

Cushing syndrome

Diabetes mellitus

Graves disease

Hashimoto thyroiditis

Hyperparathyroidism

Hypoparathyroidism

© 2026 Rao AS et al. *JAMA Network Open*.

Question 8 | Type: **Q**

Which of the following treatments can alter the natural progression of myasthenia gravis? (You may select more than one option.)

Azathioprine

IV immunoglobulin

Mycophenolate mofetil

Neostigmine

Plasma exchange

Prednisone

Pyridostigmine

© 2026 Rao AS et al. *JAMA Network Open*.

Case 12: Left Scrotal Pain in an 18-yr-old Man

Background

History of Present Illness:

An 18-yr-old man comes to the emergency department because he has had left scrotal pain for several hours. He says the pain is accompanied by nausea and began suddenly while he was shoveling snow. He continued shoveling, and the pain and nausea suddenly disappeared after a few minutes. Toward the end of shoveling, the pain and nausea returned and his scrotum started to swell, so he decided to seek medical care.

Review of Systems:

General: Overall, has felt well the past several days. Pain "came out of nowhere." Denies subjective fevers or chills.

Skin: Denies rashes, itching, or lesions.

HEENT: Noncontributory

Pulmonary: Noncontributory

Cardiovascular: Denies palpitations, chest pain, or dyspnea with exertion.

Gastrointestinal: Nausea since pain started, no vomiting. No abdominal pain. Bowel movements have been normal but none since pain began.

Genitourinary: No dysuria, hematuria, frequency or urgency, or penile discharge.

Musculoskeletal: Noncontributory

Neurologic: Noncontributory

Psychiatric: Noncontributory

© 2026 Rao AS et al. *JAMA Network Open*.

Past Medical History:

Medical history: Seasonal allergies.

Surgical history: Left ACL repair at age 16 following a basketball injury.

Medications: Daily cetirizine.

Allergies: No known drug allergies.

Family history: Father has cardiovascular disease.

Social history: He denies current or previous tobacco use; he exercises several times a week. He is a student at the local community college seeking a degree in criminal justice. All relevant immunizations are up-to-date. He denies any recent travel. He denies exposure to any sick contacts. He is sexually active with multiple partners and reports inconsistent condom usage.

Physical Examination:

General appearance: Appears nontoxic but seems uncomfortable on the exam bed.

Vital signs:

Temperature 37¬∞ C

Pulse 99/min

BP 120/60 mm Hg

Respirations: 16/min

Height: 5' 8" (1.73 m)

Weight: 160 lb (73 kg)

Height: 5' 8" (1.73 m)

BMI: 24.1

© 2026 Rao AS et al. *JAMA Network Open*.

Skin: Warm and dry to the touch.

HEENT: Normocephalic without evidence of trauma.

Pulmonary: Breath sounds are present and equal bilaterally. No stridor, rales, or wheezing.

Cardiovascular: Heart rate is regular. Radial pulses are present and equal bilaterally.

Gastrointestinal: Abdomen appears nondistended; no ecchymoses or other visible lesions. Bowel sounds are normal. Abdomen is soft, nontender to light and deep palpation with no guarding, rigidity, or rebound.

Genitourinary: Circumcised penis with no discharge. Left side of scrotum is swollen and extremely tender. It is difficult to palpate left testis secondary to extreme pain. Right testis has normal texture and is normal on palpation but does have a horizontal lie. No tenderness or fullness over the inguinal canals. Negative left cremasteric reflex.

Musculoskeletal: No costovertebral angle tenderness.

Neurologic: Unremarkable

Mental status: Alert and oriented to person, place, time, and event.

Questions

Question 1 | Type: DIFF

At this time, which of the following are the most likely differential diagnoses? (You may select more than one option.)

Epididymitis/orchitis

Groin muscle strain

Incarcerated inguinal hernia

© 2026 Rao AS et al. *JAMA Network Open*.

Nephrolithiasis

Testicular cancer

Testicular torsion

Testicular fracture

Question 2 | Type: **DIAG**

At this time, which of the following are the most appropriate immediate steps to order? (You may select more than one option.)

Basic metabolic profile (BMP)

Complete blood count (CBC)

Doppler ultrasonography of the scrotum

Nuclear scan of scrotum

Surgical exploration of the scrotum

Urethral swab for sexually transmitted infection

Urinalysis

© 2026 Rao AS et al. *JAMA Network Open*.

Question 3 | Type: **DX**

Test result 1

Urinalysis:

Test (units)

Result

Color: Yellow

pH: 5.8

Specific gravity

1.010

RBCs: 0-2/hpf

WBCs: 0-1/hpf

Leukocyte esterase: Negative

Nitrites: Negative

Bacteria: Negative

Doppler ultrasonography of scrotum:

Heterogeneous-appearing left testis, with no demonstrated venous or arterial Doppler blood flow. Normal-appearing right testis with normal venous and arterial blood flow.

At this time what is the most likely diagnosis? (Please select only one option.)

Acute epididymitis

Incarcerated inguinal hernia

Testicular appendage torsion

Testicular torsion

Question 4 | Type: **MANG**

© 2026 Rao AS et al. *JAMA Network Open*.

A diagnosis of testicular torsion is confirmed.

At this time, which of the following is the most appropriate management? (You may select more than one option.)

Administer ciprofloxacin 500 mg IV

Administer ibuprofen 800 mg po

Administer morphine 8 mg IV

Admit the patient to the hospital for bed rest and scrotal elevation, with repeat ultrasonography in 12 h

Consult a urologist for immediate surgical exploration

Do manual detorsion at the bedside

Question 5 | Type: **Q**

At which age are patients at higher risk of testicular torsion? (You may select more than one option.)

Birth to 1 yr

8 to 10 yr

12 to 18 yr

© 2026 Rao AS et al. *JAMA Network Open*.

40 to 50 yr

60 to 70 yr

Question 6 | Type: **Q**

Which deformity in the development of the testes is most associated with increased risk for intravaginal testicular torsion? (You may select more than one option.)

Bell-clapper deformity

Inguinal hernia

Patent processus vaginalis

Spermatic cord lipoma

Question 7 | Type: **Q**

Testicular torsion can quickly cause ischemic necrosis of the testis. On average, how soon after onset of pain do testicular salvage rates drop close to zero percent? (You may select only one option.)

1 h

6 h

12 h

24 h

© 2026 Rao AS et al. *JAMA Network Open*.

48 h

© 2026 Rao AS et al. *JAMA Network Open*.

Case 13: Left-sided Abdominal Pain in a 45-yr-old man

Background

History of Present Illness:

A 45-yr-old man comes to the emergency department because of a 6-h history of acute-onset left-sided lower abdominal pain. He describes the pain as starting over his left flank and radiating down toward his groin. The pain comes and goes but is intense when present. He has had some mild nausea without vomiting. The pain does not change with eating. The pain is less when he walks around than when he sits or lies down flat. He has not had any bloody stools, black stools, or change in bowel habits. He denies having diarrhea and constipation. He has noticed his urine looks "dark" but denies pain during urination and urinary frequency. He says that he swims several times a week for exercise, and this pain started immediately after swimming. He also notes that several days ago, his son ran into his left side while playing football. He took some acetaminophen, which minimally relieved his discomfort. He now presents for evaluation at the emergency department because the pain has not subsided. He says he has never experienced pain like this.

Review of Systems

General: Has otherwise felt well before onset of his current symptoms. Denies fevers, chills, weight loss, and fatigue.

Skin: Denies rashes, itching, and any lesions.

HEENT: Noncontributory

Pulmonary: Noncontributory

Cardiovascular: Denies palpitations and chest pain. No lower-extremity edema.

© 2026 Rao AS et al. *JAMA Network Open*.

Gastrointestinal: Reports nausea when pain is at its most intense but no vomiting. No constipation or diarrhea. Last bowel movement yesterday; no hematochezia or melena. No appetite; he has not eaten since the pain began.

Genitourinary: No dysuria, hematuria, urinary frequency or urgency, or increased volume of urine. States urine appears "dark."

Musculoskeletal: Occasional low back pain with strenuous activity

Neurologic: Noncontributory

Psychiatric: Noncontributory

Past Medical History:

Diabetes mellitus since his 20s that is well-controlled with oral drugs, diet, and exercise. Hypertension and hyperlipidemia both well-controlled on drugs. Obstructive sleep apnea for which he uses a CPAP machine. Recent deep venous thrombosis that developed after a long international flight and that is currently being treated with an oral anticoagulant. Denies history of UTIs, difficulty with urination, COPD, renal stones, and immunosuppressive disorders.

Surgical history: Wisdom tooth extraction as a teenager; no abdominal surgical procedures.

Medications: Lisinopril 10 mg daily, aspirin 81 mg daily, rosuvastatin 10 mg daily, rivaroxaban 20 mg daily, metformin 850 mg daily, and niacin 1 g daily.

Allergies: No known drug allergies.

© 2026 Rao AS et al. *JAMA Network Open*.

Family history: Mother and father have hypertension; sibling has history of kidney stones.

Social history: He denies current and previous tobacco use. He works at a desk job and swims laps at his local pool several times a week. All immunizations are up-to-date. He denies exposure to any sick contacts. For the past 15 yr, he has had unprotected sex only with his wife.

Physical Examination

General appearance: Appears nontoxic but seems uncomfortable on the exam bed.

Vital signs:

Temperature: 37¬∞ C

Pulse: 99 beats/min

BP: 150/90 mm Hg

Respirations: 16/min

Height: 6 ft (1.83 m)

Weight: 300 lb (136 kg)

BMI: 40.7

Skin: Warm and dry to the touch. No rash or lesions.

HEENT: Unremarkable

Pulmonary: Breath sounds present and equal bilaterally. No stridor, rales, or wheezing.

© 2026 Rao AS et al. *JAMA Network Open*.

Cardiovascular: Regular rate and rhythm; no murmurs or rubs. Radial and pedal pulses present and equal bilaterally. No pedal edema.

Gastrointestinal: No ecchymoses. Bowel sounds normal in all quadrants. Abdomen nondistended, soft, and nontender to light and deep palpation; no guarding, rigidity, rebound, or masses. Negative Murphy sign. Rectal exam is heme-negative.

Genitourinary: Circumcised penis, bilateral descended testes. Testes and epididymal bodies nontender to palpation. No costovertebral angle tenderness.

Musculoskeletal: Muscle strength is intact in all four limbs without any tenderness to palpation or movement.

Neurologic: Unremarkable

Mental status: Alert and oriented to person, place, time, and event.

Questions

Question 1 | Type: **DIFF**

At this time, which of the following differential diagnoses cannot be excluded based on history and physical examination in this patient? (You may select more than one option.)

Appendicitis

Bowel obstruction

Cholecystitis

© 2026 Rao AS et al. *JAMA Network Open*.

Colon cancer

Dissecting aortic aneurysm

Diverticulitis

Epididymitis

Gastroenteritis

Ischemic colitis

Nephrolithiasis

Pancreatitis

Splenic laceration or contusion

Urinary tract infection (UTI)

Question 2 | Type: **DIAG**

Given the history and physical exam findings, which of the following are the most appropriate steps to guide diagnosis and treatment? (You may select more than one option.)

Abdominal ultrasonography

© 2026 Rao AS et al. *JAMA Network Open*.

Blood cultures

CBC

Basic metabolic profile (BMP)

CT of the abdomen and pelvis with po and IV contrast

CT of the abdomen and pelvis without contrast

Plain x-rays of abdomen

Urine culture

Urinalysis

Question 3 | Type: **DX**

Image: *13_DX_CT1, 13_DX_CT2, 13_DX_CT3*

Results of appropriate testing return and show the following:

Urinalysis:

Test (units)

Result

Color: Yellow

pH: 5.8

Specific gravity

© 2026 Rao AS et al. *JAMA Network Open*.

1.01

RBCs

20-25/HPF

WBCs

0-1/HPF

Leukocyte esterase

Negative

Nitrites

Negative

Bacteria

Negative

CBC:

Test (units)

Result

Hemoglobin

14 g/dL

WBC count

5000/mcL

Platelet count

150,000/mcL

Basic metabolic profile:

Test (units)

Result

Sodium

140 mEq/L

Potassium

4.0 mEq/L

Creatinine

0.7 mg/dL

Glucose

105 mg/dL

© 2026 Rao AS et al. *JAMA Network Open*.

CT scans without contrast of the abdomen and pelvis are obtained and are shown.
Select each image to review.

At this time, which of the following is the most likely diagnosis. (You may select only one option.)

Ureteral lithiasis

Dissecting aortic aneurysm

Diverticulitis

Splenic laceration or contusion

UTI

Question 4 | Type: **MANG**

At this time, which of the following are the most appropriate management steps? (You may select more than one option.)

Admit the patient to the hospital

Discharge from the emergency department with a urine strainer

Percutaneous nephrolithotomy

Ureteroscopic stone removal

© 2026 Rao AS et al. *JAMA Network Open*.

Shock wave lithotripsy

0.9 % saline solution, 1 L IV over 1 h

Ciprofloxacin 500 mg po bid for 10 days

Ketorolac 15 mg IV

Ondansetron 4 mg IV

Oxycodone / acetaminophen 5 mg/ 325 mg po q 6 h for 7 days

Question 5 | Type: **Q**

Statistically, what is the most likely composition of this patient's ureteral stone? (You may select only one option.)

Calcium oxalate

Calcium phosphate

Cystine

Magnesium ammonium phosphate (struvite)

Uric acid

© 2026 Rao AS et al. *JAMA Network Open*.

Question 6 | Type: **Q**

Which of the following is the only stone type that can realistically be dissolved and thus eliminate the need for surgical intervention? (You may select only one option.)

Calcium phosphate

Calcium oxalate

Cystine

Uric acid

Question 7 | Type: **Q**

In addition to oral pain medication, which other drug should be prescribed for a patient undergoing a trial of medical expulsive therapy for a ureteral stone? (You may select only one option.)

Dutasteride 0.5 mg daily

Finasteride 5 mg daily

Hydrochlorothiazide 12.5 mg daily

Ondansetron 4 mg q 8 h as needed

Question 8 | Type: **Q**

Which of these urinary characteristics increases the risk of stone formation in the urinary tract? (You may select only one option.)

Hyperglycosuria

© 2026 Rao AS et al. *JAMA Network Open*.

Hypocalciuria

Hypocitruria

Hypooxaluria

Hypouricosuria

Specific gravity > 1.015

© 2026 Rao AS et al. *JAMA Network Open*.

Case 14: Leg Pain in a 62-yr-old man

Background

History of Present Illness:

A 62-yr-old man comes to the emergency department in December because he has had 2 days of progressively worsening pain in his right lower leg between the knee and ankle. He says that several hours before arrival in the emergency department, he began to feel generally ill and aching all over. He thought he had a fever but did not take his temperature. He had been in his usual state of generally good health until 2 days ago. He denies any recent injury to the right leg although he notes that since his coronary artery bypass graft (CABG) surgery, that leg is often slightly swollen at the end of the day but is never painful.

Review of Systems:

General: Modestly overweight for most of his adult life.

Skin: States that he has a red area on the right leg that is slightly painful. Denies other skin lesions, rash, or itching.

HEENT: Noncontributory

Pulmonary: No cough, shortness of breath, or pleuritic chest pain.

Cardiovascular: No chest pain, dyspnea during exertion, palpitations, orthopnea, or paroxysmal nocturnal dyspnea.

Gastrointestinal: No nausea, vomiting, or diarrhea. Appetite uncharacteristically poor today.

Genitourinary: No increased volume or frequency of urination; no dysuria. Sexually active only with his wife.

Musculoskeletal: Other than as noted in the history above, no pain in limbs or joints. No recent injuries or immobilization.

Neurologic: Noncontributory

© 2026 Rao AS et al. *JAMA Network Open*.

Psychiatric: Noncontributory

Past Medical History:

Medical history: The patient has had type 2 diabetes for 5 yr, controlled by diet. He does not measure glucose levels at home. His glucose and HbA1c levels were last checked 6 mo ago, but he does not know the results. Coronary artery disease was diagnosed 7 yr ago requiring coronary artery bypass grafting (CABG). No current angina; no history of myocardial infarction. Hypertension and hypercholesterolemia since age 50, controlled on medications. No history of cancer, HIV, blood clots or clotting disorder. No recent hospitalizations.

Surgical history: CABG 7 yr ago, with harvesting of his right saphenous vein.

Medications: Hydrochlorothiazide 25 mg once/day, enalapril 40 mg once/day, aspirin 81 mg once/day, pravastatin 40 once/day.

Allergies: No known drug allergies; no seasonal allergies.

Family history: Mother alive; age 80 with hypertension, type 2 diabetes, and high cholesterol. Father died at age 64 of myocardial infarction.

Social history: Employed as a police officer, has been going to work as usual until today. He plays squash and/or basketball twice a week. He adheres to a low-fat diet and consumes 2 to 3 alcoholic drinks/wk. He smoked 1 pack of cigarettes daily for 20 yr but quit at age 44. Denies other recreational drug use, including parenteral use or skin-popping. He has not traveled outside the northeast US in more than 10 yr. No pets. Up to date on vaccines.

Physical Examination:

General appearance: Middle-aged male in no apparent distress.

© 2026 Rao AS et al. *JAMA Network Open*.

Vital signs:

Temperature: 38.5¬∞ C

Pulse: 118 beats/min

BP: 128/74 mmHg

Skin: Diffusely flushed and diaphoretic. No rash or lesions, except for a 15 x 10 cm area of erythema on the lateral aspect of the right calf; the area is warm, indurated, and mildly tender. No bullae, crepitus, or fluctuance. No visible wounds, skin breaks, or abrasions. No athleteÔøΩs foot. Has a healed scar on the right leg running from ankle to mid-thigh from saphenous vein harvesting.

HEENT: Unremarkable

Neck: Normal carotid upstrokes. Jugular venous distention 12 cm above sternal notch at 45¬∞.

Pulmonary: Normal breath sounds bilaterally without crackles, rhonchi, or rubs.

Cardiovascular: Tachycardia at 120 beats/min. No gallops, murmurs, or rubs. Pulses 2+ and symmetric bilaterally. No bruits.

Gastrointestinal: Unremarkable

Genitourinary: Unremarkable

Musculoskeletal: In right leg shows 1+ pitting edema over the shin; right calf diameter is 2-cm greater than that of the left. No calf tenderness to compression although the erythematous area noted on the skin exam is mildly tender. No visibly or palpably distended veins. No clubbing or splinter hemorrhages. Left leg appears normal.

Neurologic: Unremarkable

Mental status: Alert, oriented to person, place, and time.

Questions

Question 1 | Type: **DIFF**

© 2026 Rao AS et al. *JAMA Network Open*.

At this time, which of the following differential diagnoses cannot be excluded based on history and physical examination in this patient? (You may select more than one option.)

Abscess

Allergic reaction

Cellulitis

Contusion

Deep vein thrombosis (DVT)

Erythema migrans

Insect sting

Necrotizing soft-tissue infection

Superficial thrombophlebitis

Question 2 | Type: **DIAG**

At this time, which of the following are the most appropriate immediate steps to order? (You may select more than one option.)

No additional testing is needed at this time (clinical diagnosis only)

© 2026 Rao AS et al. *JAMA Network Open*.

Basic metabolic panel (Na+, Cl-, K+, HCO_3-, BUN, creatinine, glucose)
Blood cultures
Complete blood count (CBC)
Chest x-ray
Doppler ultrasonography of leg
ECG
Leg x-ray
Skin cultures

Question 3 | Type: **DX**

Results of appropriate testing return and show the following:

Blood:

Test (units)

Result

Hemoglobin

13 g/dL

Platelets

310,000/mcL

WBC

14,500/mm3

Neutrophils

86%

Bands

8%

Serum:

Test (units)

Result

Glucose

220 mg/dL

Blood urea nitrogen (BUN)

28 mg/dL

Creatinine

1.4 mg/dL

© 2026 Rao AS et al. *JAMA Network Open*.

Na+

135 mmol/L

K+

4.2 mmol/L

Cl-

100 mmol/L

HCO3-

21 mmol/L

Ketones

Negative

Electrocardiogram (ECG): Sinus tachycardia at 120 beats beats/min; no change compared with 5 years yr ago.

X-ray right lower leg: Normal

Venous Doppler venous studyultrasonography, right lower leg: Normal

At this time, which of the following is the most likely diagnosis for this patient? (Please select only one option.)

Abscess

Cellulitis

Deep vein thrombosis (DVT)

Necrotizing subcutaneous infection

Question 4 | Type: **MANG**

Which of the following choices are the most appropriate management of this patient's condition? (You may select more than one option.)

Admit the patient to the hospital

© 2026 Rao AS et al. *JAMA Network Open*.

Encourage ambulation

Elevate the right leg while in bed

Blood cultures

Cephalexin 500 mg po every q 6 h

Diphenhydramine 50 mg po every q 6 h

Ibuprofen 600 mg po every q 6 h as needed for pain

Morphine 8 mg IV every q 6 h as needed for pain

Nafcillin 1 g IV every q 6 h

Tobramycin 100 mg IV every q 8 h plus piperacillin 3 g IV every q 4 hr

Vancomycin 1 g IV every q 12 h

Question 5 | Type: **Q**

Which of the following antimicrobials are appropriate treatments for mild cellulitis? (You may select more than one option.)

© 2026 Rao AS et al. *JAMA Network Open*.

Cephalexin

Chloramphenicol

Dicloxacillin

Gentamicin

Levofloxacin

Tetracycline

Question 6 | Type: **Q**

Which of the following factors affects the choice of antimicrobial therapy in patients with cellulitis? (You may select more than one option.)

High prevalence of MRSA in the community

History of upset stomach after taking penicillin

History of hives after taking penicillin

Intensity of pain at the infected site

Presence of regional adenopathy

© 2026 Rao AS et al. *JAMA Network Open*.

Previous splenectomy

Size of the area of erythema

Question 7 | Type: **Q**

Which of the following factors suggest that a patient with cellulitis needs to be admitted to the hospital? (You may select more than one option.)

Delirium

Hypotension

Leukocytosis

Low-grade fever

Lymphangitis

Vomiting

Question 8 | Type: **Q**

Which features, if present, help differentiate cellulitis and DVT of the leg? (You may select more than one option.)

High fever

Leg swelling

© 2026 Rao AS et al. *JAMA Network Open*.

Lymphangitis

Low-grade fever

Peau d'orange skin

Positive Homan sign

© 2026 Rao AS et al. *JAMA Network Open*.

Case 15: Leg tingling in a 20-yr-old woman

Background

History of present illness:

A 20-yr-old woman comes to the office because of a 1-wk history of a feeling she describes as "novocaine wearing off" extending from her waist to her toes on both sides of her body. She says this feeling has been worsening. She has had no prior viral illness and has not received any recent vaccinations or had any recent injuries. She has no weakness in her arms or legs, but says she may be tripping more than usual; however, she has not fallen. She thinks her fingertips feel "funny" as well. She has no headache, face pain, face numbness, double vision, or other visual complaints. She does not have dysarthria, dysphasia, or difficulty swallowing. She says that when wiping her perineal region, it feels "funny." She had an episode of left leg numbness 2 yr ago that lasted 3 wk and that resolved by itself.

Review of systems:

General: She has felt well until this current episode began and has had no fevers, chills, or recent weight loss.

Skin: No rash or lesions.

HEENT: Noncontributory

Pulmonary: Noncontributory

Cardiovascular: Born with double outlet right ventricle, corrected at birth, then stented in 2005 because of residual scarring. Gets annual cardiology check-ups.

Gastrointestinal: Noncontributory

Genitourinary: Normal bladder and bowel function. Last menstrual period was 2 wk ago.

Musculoskeletal: No pain in neck, back, or joints.

Neurologic: See previous screen (History of Present Illness).

© 2026 Rao AS et al. *JAMA Network Open*.

Psychiatric: She says her mood and general spirits have been good until this current.

Past Medical History

Medical history: Migraine headaches several times per year, last episode 4 mo prior.

Surgical history: Born with double outlet right ventricle, corrected at birth, then stented in 2005 because of residual scarring. Gets annual cardiology check-ups.

Medications: None

Allergies: None

Family history: Noncontributory

Social history: Regular diet, does not exercise. No history of recent travel. No drug, alcohol, or tobacco use. Immunizations up-to-date, no recent exposure to illness. Sexually active with one partner; uses condoms consistently.

Physical Examination

General appearance: Young woman in no apparent distress.

Vital signs:

Temperature: 37¬∞ C

Pulse: 75 beats/min

BP: 120/80 mm Hg

Respirations: 12/min

Skin: Warm, dry; no erythema or rash.

© 2026 Rao AS et al. *JAMA Network Open*.

HEENT: Unremarkable

Pulmonary: Unremarkable

Cardiovascular: Unremarkable

Gastrointestinal: Unremarkable

Genitourinary: Unremarkable

Musculoskeletal: No visible or palpable lesions or tenderness along spine. Joints nontender, full range of motion.

Neurologic:

Cranial nerves: 2 through 12 intact

Motor exam: Strength and muscle tone normal in both upper and both lower extremities. No muscle atrophy or abnormal movements seen.

Sensory exam: Sensation intact bilaterally to light touch, pinprick, temperature, vibration, and joint position

Reflexes: Normoactive and symmetric throughout

Coordination: Normal

Gait and balance: Normal

Detailed movement exam: Normal

Rectal examination: Normal perineal sensation and normal rectal sphincter tone

Mental status: Alert, oriented x3. Normal affect. Euthymic mood. Normal memory, judgment, and insight.

*

Questions

Question 1 | Type: Q

At this time, based on history and physical examination, which of the following components of the nervous system are most likely to be involved in this patient's symptoms? (You may select more than one option.)

© 2026 Rao AS et al. *JAMA Network Open*.

Autonomic ganglia

Cerebral cortical sensory areas

Midbrain

Peripheral nerves

Spinal cord

Question 2 | Type: **DIFF**

At this time, which of the following differential diagnoses cannot be excluded based on history and physical examination in this patient? (You may select more than one option.)

Acute viral infection

A connective tissue disorder

Lumbosacral plexitis

Myelopathy due to vitamin deficiency

Multiple sclerosis (MS)

Peripheral neuropathy due to toxins

© 2026 Rao AS et al. *JAMA Network Open*.

Sarcoidosis

Spinal cord compression by tumor

Spinal cord hemorrhage

Spinal epidural abscess

Question 3 | Type: **DIAG**

At this time, the most appropriate immediate next steps are? (You may select more than one option.)

Carotid Doppler ultrasonography

CT of chest and abdomen

Doppler ultrasonography of the legs

Electromyography (EMG) of the legs

MRI of the cervical spine

MRI of the thoracic spine

© 2026 Rao AS et al. *JAMA Network Open*.

X-ray of the thoracic spine

Question 4 | Type: **DX**

🖼 **Image:** *15_DX_MRI1, 15_DX_MRI2, 15_DX_MRI3, 15_DX_MRI4, 15_DX_MRI5, 15_DX_MRI6, 15_DX_MRI7*

Results of the MRI return and are shown below.

Please review the result by clicking on each image and answer the question below.

MRI OF THORACIC SPINE

MRI OF CERVICAL SPINE

Which of the following is a correct interpretation of the MRI of the thoracic and cervical spine? (You may choose only one option.)

Intra-axial spinal cord tumor

Spinal cord abscess

Spinal cord infarction

Transverse myelitis

Question 5 | Type: **MANG**

Given the MRI findings, which of the following are the most appropriate steps to guide diagnosis and treatment? (You may select more than one option.)

CT of the spine

Doppler studies of the legs

© 2026 Rao AS et al. *JAMA Network Open*.

EEG

MRI of the brain

Nerve conduction studies and electromyography

Spinal tap

Question 6 | Type: **DX**

🖼 **Image:** *15_DX_MRI8, 15_DX_MRI9, 15_DX_MRI10, 15_DX_MRI11*

MRI of the brain is obtained and is shown.

Please review the result by clicking on each image and answer the question below.

Which of the following is a correct interpretation of the MRI? (You may choose only one option.)

Normal findings

Multifocal metastases

Multifocal strokes

Primary demyelinating disease

Question 7 | Type: **DX**

At this time what is the most likely diagnosis?

Acute disseminated encephalomyelitis (ADEM)

Infectious meningoencephalomyelitis

© 2026 Rao AS et al. *JAMA Network Open*.

Multiple sclerosis

Sarcoidosis

Question 8 | Type: **MANG**

A diagnosis of multiple sclerosis is confirmed. Which of the following can be considered initial therapies for this patient? (You may choose more than one option.)

Azathioprine 3 mg/kg once/day

Cyclophosphamide 2 mg/kg po once/day

Dimethyl fumarate 120 mg po bid for 7 days

Interferon beta-1a, 30 mcg IM once/wk

Intravenous immune globulin (IVIG): 0.4 g/kg/day for 5 days

Methylprednisolone 1 g IV once per day for 3 d

Mitoxantrone 12 mg/m2 IV

Natalizumab 300 mg IV q 4 wk

Question 9 | Type: **Q**

What are the symptoms and signs of transverse myelitis? (You may select more than one option.)

© 2026 Rao AS et al. *JAMA Network Open*.

Dysarthria

Dysphasia

Sensory loss below the level of the lesion

Sphincteric and sexual dysfunction

Visual loss

Weakness below the level of the lesion

Question 10 | Type: **Q**

Which of the following are associated with a higher risk of developing multiple sclerosis? (You may select more than one option.)

Alcohol use

Cigarette smoking

Exposure to human papillomavirus (HPV)

Female sex

Human leukocyte antigen (HLA) DR2 allotype

© 2026 Rao AS et al. *JAMA Network Open*.

Living in temperate climate during their first 15 yr of life

Obesity

Question 11 | Type: **Q**

Which of the following ocular findings are commonly seen in patients with multiple sclerosis (MS)? (You may select more than one option.)

Internuclear ophthalmoplegia

Marcus-Gunn pupil

Optic neuritis

Proptosis

Question 12 | Type: **Q**

Which is the most common subtype of multiple sclerosis? (You may choose only one option.)

Primary progressive

Progressive - relapsing

Relapsing - remitting

Secondary progressive

© 2026 Rao AS et al. *JAMA Network Open*.

Case 16: Lethargy in a 65-yr-old man

Background

History of Present Illness

A 65-yr-old man is brought to the emergency department (ED) because his neighbors noted that he was lethargic and confused. When emergency medical services (EMS) arrived at the man's home, they found an empty oxycodone pill bottle next to him, and they noted that he had pinpoint pupils. The neighbors stated that the patient has a history of chronic pain. In the ED, he is given naloxone 0.4 mg IV, resulting in prompt improvement in his mental status. Thereafter, the patient is able to answer questions and states that he wanted to die and had taken an overdose of several of his medications in an attempt to end his life. He is not sure exactly which medications he took.

Review of Systems

General: No fevers, chills, weight loss, or fatigue.

Skin: No rash or skin lesions; no excessive sweating.

HEENT: No vision changes; no excessive salivation or lacrimation.

Pulmonary: No cough, shortness of breath, or wheezing.

Cardiovascular: No chest pain or pressure; no palpitations or lower-extremity edema.

Gastrointestinal: No nausea or vomiting; no constipation; no diarrhea; no abdominal pain.

Genitourinary: No hematuria, dysuria, or change in urine output.

Musculoskeletal: Chronic low back pain since a motor vehicle accident 7 yr ago; no recent falls or injuries.

Neurologic: No numbness or weakness of extremities; no headache.

Psychiatric: Describes depressed mood and suicidal ideation; admits he attempted to end his life today by ingesting his medications.

Past Medical History

Medical history: Hypertension since age 47 yr that is moderately controlled with three antihypertensive drugs. Hypercholesterolemia that was diagnosed in his early 50s and for which he takes an unknown drug. Depression for which he takes an unknown drug. Chronic back pain for which he takes oxycodone. No history of stroke or transient ischemic attacks, coronary artery disease, renal disease, cancer, diabetes, or autoimmune disorders.

Surgical history: Negative

© 2026 Rao AS et al. *JAMA Network Open*.

Medications: Oxycodone and unknown antihypertensive drugs, lipid-lowering drug, and antidepressant.

Allergies: No known drug or environmental allergies.

Family history: One brother with hypertension. Father died at age 76 from complications of myocardial infarction.

Social history: Patient is divorced with 2 children. He used to work as a bus driver but is on disability because of chronic back pain. He used to smoke cigarettes but quit 10 yr ago. He drinks 2 to 3 beers daily. He smokes marijuana once or twice/yr.

Physical Examination

General appearance: Height is 5' 7"; weight is 240 lb. Patient appears nontoxic and in no apparent distress.

Vital signs:

Temperature: 37.2¬∞ C

Pulse: 45 beats/min

BP: 99/72 mm Hg

Respirations: 20/min

Pulse oximetry: 97% on room air

Skin: Warm, dry, normal color, and well-perfused.

HEENT: Pupils equal, 5 mm, and reactive to light; no scleral icterus. Head appears normal and no evidence of trauma; mucous membranes are moist.

Pulmonary: Lungs clear to auscultation and percussion bilaterally; no rales, rhonchi, or wheezes.

Cardiovascular: Regular rate and rhythm; no murmurs, rubs, or gallops.

Gastrointestinal: No abdominal scars observed. Abdomen soft, nontender, and nondistended. No pulsating masses or organomegaly palpated. Bowel sounds auscultated in all four quadrants. Rectal examination normal.

Genitourinary: Not examined

Musculoskeletal: Normal tone and strength; 2+ deep tendon reflexes equal bilaterally.

© 2026 Rao AS et al. *JAMA Network Open*.

Neurologic: Cranial nerves 2 to 12 intact. Bilateral sensation to touch, vibration, and pinpoint intact. Negative pronator drift. Finger-to-nose movement intact. Speech and cognition within normal limits. Gait normal.

Mental status: Flat affect. Alert and oriented to self, place, and time.

*

Shortly after initial examination, the patient becomes extremely lethargic. He responds to deep pain by withdrawing, and he is able to protect his airway. He is noted to have the following:

* Heart rate 30 beats/min
* BP 74/52 mm Hg
* Respirations 10/min
* Pulse oximetry 94% on room air.
* His pupils remain equal and reactive.

Questions

Question 1 | Type: DIFF

At this time, essential differential diagnoses should include which of the following? (You may select more than one option.)

Adrenal insufficiency

Beta-blocker overdose

Calcium channel blocker overdose

Digoxin overdose

Opioid overdose

Elevated intracranial pressure

© 2026 Rao AS et al. *JAMA Network Open*.

Tricyclic antidepressant (TCA) overdose

Hypocalcemia

Hypoglycemia

Hypokalemia

Hypothyroidism

Myocardial infarction

Pulmonary embolism

Question 2 | Type: **MANG**

Which of the following steps are essential to do immediately? (You may select more than one option.)

2 L 0.9% normal saline IV bolus

Naloxone 0.4 mg IV and monitoring for improvement in symptoms.

Cardiac monitoring

© 2026 Rao AS et al. *JAMA Network Open*.

Bedside glucose level

Blood cultures

CBC

Serum acetaminophen level

Serum digoxin level

Serum salicylate levels

Serum thyroid- stimulating hormone (TSH) measurement

Serum electrolyte concentrations

Chest x- ray

Urine toxicology screening

Question 3 | Type: **DX**

🖼 **Image:** *16_DX_ECG*

Results of appropriate testing return and show the following:

Electrolytes:

© 2026 Rao AS et al. *JAMA Network Open*.

Test (units)
Result
Sodium
138 mEq/L
Potassium
4.0 mEq/L
Chloride
100 mEq/L
Bicarbonate
24 mEq/L

Blood:
Test (units)
Result
TSH
2.0 mcIU/mL
TSH
2.0 mcIU/mL
Acetaminophen and salicylate levels
Undetectable
Digoxin level
0
Bedside glucose
68 mg/dL

Urine:
Test (units)
Result
Toxicology screen
Positive for opioids and marijuana

The rhythm strip below is obtained.

Which of the following is a correct interpretation of this rhythm strip? (You may select only one option.)

© 2026 Rao AS et al. *JAMA Network Open*.

Atrial fibrillation

Sinus arrhythmia

Sinus bradycardia

Sinus rhythm with long QTc

Sinus rhythm with 2nd-degree (type I) AV block

Question 4 | Type: **N/A**

After receiving 2 L normal saline and 0.4 mg naloxone IV, the patient's mental status is unimproved. His pulse is now 30 beats/min and BP is 76/54 mm Hg.

At this time what is the most likely diagnosis? (You may select only one option.)

Beta-blocker overdose

Calcium channel blocker overdose

Digoxin overdose

Marijuana overdose

© 2026 Rao AS et al. *JAMA Network Open*.

Opioid overdose

Hypocalcemia

Hypoglycemia

Hypokalemia

Hypothyroidism

Question 5 | Type: **MANG**

At this time, which of the following are the most appropriate management? (You may select more than one option.)

Admit the patient to the general medicine floor

Schedule outpatient psychiatry follow-up

Cardiac pacing

Atropine 0.5 to 1 mg IV q 3 to 5 min

Epinephrine 1 mcg/min IV

Glucagon 5 mg over 1 min IV followed by 2 to 5 mg/h IV infusion

© 2026 Rao AS et al. *JAMA Network Open*.

Question 6 | Type: **Q**

Which of the following symptoms are typically present in opioid overdose? (You may select more than one option.)

Bradycardia

Dilated pupils

Lethargy

Hyperkalemia

Hypothermia

Hypotension

Tachypnea

Question 7 | Type: **Q**

Which of the following can be used in treatment of beta-blocker overdose? (You may select more than one option.)

Activated charcoal 1 g/ kg by mouth or nasogastric tube

Calcium chloride 1 g via central line

Calcium chloride 1 g via peripheral IV

© 2026 Rao AS et al. *JAMA Network Open*.

Calcium gluconate 1 g via peripheral IV

High-dose insulin (bolus of 1 unit/ kg of regular, short - acting insulin, then 0.5 units/ kg / h IV) and glucose therapy titrated to maintain normal glucose levels

Lipid emulsion therapy 1.5 mL/ kg of 20% solution over 2 min, then 1.5 mL/ kg over 60 min

Question 8 | Type: **Q**

Which symptoms and signs are typical of the Cushing reflex? (You may select more than one option.)

Bradycardia

Central obesity

Cheyne-Stokes respiration

Dorsal fat pad

Hypertension

Moon facies

Respiratory depression

© 2026 Rao AS et al. *JAMA Network Open*.

Widened pulse pressure

© 2026 Rao AS et al. *JAMA Network Open*.

Case 17: Loss of consciousness in a 50-yr-old man

Background

History of Present Illness

A man who appears to be in his 50s is brought to the emergency department. The history is given by emergency medical technicians (EMTs), who state they were called to a store for a 50-yr-old man who possibly had a stroke. At the store, they found him lying on the ground unconscious but breathing normally. After their arrival, the patient awoke but was unable to give coherent responses to questions or commands.

Store employees who saw the patient reported to EMTs that he fell to the ground suddenly from a sitting position, then began drooling, and his arms and legs shook for what seemed to be a long time, but they cannot quantify how long. They state he had not appeared ill before the event.

Review of Systems

Patient is unable to provide any information at this time.

Past Medical History

Not available at this time.

Physical Examination

General apperance: Awake; saliva and blood at corner of his mouth. He appears well-developed, and appearance is consistent with stated age.

Vital signs:

Temperature: 36.5¬∞ C (97.7¬∞ F)

Pulse: 80 beats/min and regular

BP: 165/90 mm Hg

Respirations: 20/min

© 2026 Rao AS et al. *JAMA Network Open*.

Skin: Pink, warm, and dry, with a few punctate red spots on right lower leg

HEENT: No evidence of scalp trauma. Tympanic membranes are clear; there is a puncture wound on the bottom of his tongue. HEENT exam is otherwise unremarkable.

Pulmonary: No respiratory distress. Equal breath sounds bilaterally with good air entry/exit; no wheezing.

Cardiovascular: Regular rate and rhythm. Normal S1 and S2. No murmurs.

Gastrointestinal: Unremarkable

Genitourinary: Unremarkable except for urine on trousers.

Musculoskeletal: Full range of motion; no deformities, tenderness, or bruises.

Neurologic: Pupils are equal and normally reactive to light with no nystagmus. Face appears symmetric; gag reflex is present and symmetric. The patient can move all limbs equally to command but patient does not comprehend commands for more detailed motor function testing. Deep tendon reflexes are symmetric and 2+ throughout; plantar reflexes are downgoing bilaterally. No meningismus.

Mental status: Awake; eyes open. Oriented only to name; gives confused, incoherent responses to other questions; follows simple commands using all limbs.

* Shortly after initial examination, the patient becomes extremely lethargic. He responds to deep pain by withdrawing, and he is able to protect his airway.

© 2026 Rao AS et al. *JAMA Network Open*.

He is noted to have the following: heart rate 30 beats/min, BP 74/52 mm Hg, respirations 10/min, and pulse oximetry 94% on room air. His pupils remain equal and reactive.

Questions

Question 1 | Type: DIFF

At this time, which of the following differential diagnoses cannot be excluded based on history and physical examination in this patient? (You may select more than one option.)

Alcohol intoxication

Alcohol withdrawal

Cardiac arrhythmia

Hypoglycemia

Hyperventilation syndrome

Intracerebral hemorrhage

Meningitis

Opioid overdose

Orthostatic hypotension

© 2026 Rao AS et al. *JAMA Network Open*.

Psychiatric disorder

Pulmonary embolism (PE)

Seizure

Stroke affecting the posterior circulation

Subarachnoid hemorrhage

Vasovagal syncope

Question 2 | Type: **MANG**

Which of the following steps are essential to do immediately? (You may select more than one option.)

Bedside glucose measurement

BP measurement every 5 min until stable

Cardiac monitoring

Cold caloric testing

Dextrose 50 g IV

© 2026 Rao AS et al. *JAMA Network Open*.

Endotracheal intubation

Lumbar puncture

Naloxone 0.4 mg IV

Pulse oximetry

Toxicology screening

Question 3 | Type: **MANG**

Appropriate testing is done and results are shown:

Normal sinus rhythm at 80 beats/min on cardiac monitor

Pulse oximetry: 96% on room air

ECG: Sinus rhythm; no signs of arrhythmia or ischemia

Bedside glucose: 94 mg/dL

Given the previous findings, which of the following are the most appropriate steps to guide diagnosis and treatment? (You may select more than one option.)

Carotid Doppler testing

CBC

ECG

© 2026 Rao AS et al. *JAMA Network Open*.

Head CT

Lumbar puncture for examination of CSF

Reexamination of the patient

Seek additional history from patient's family and records

Serum chemistry profile

Tilt table testing

Toxicology screen

Question 4 | Type: **DX**

Image: *17_DX_CT*

Results of appropriate studies and additional information gathering show the following:

Blood:

Test (units)

Result

Hemoglobin

15.2 g/dL

Hematocrit

45.4%

WBC count

© 2026 Rao AS et al. *JAMA Network Open*.

9700/mcL

Serum chemistry profile: Normal

ECG: Sinus rhythm; no signs of arrhythmia or ischemia

The patient's family has now arrived at the hospital. They state that:

Medical history: Osteoarthritis; intermittent, well-controlled asthma; no recent hospitalizations

Surgical history: Tonsillectomy during childhood

Medications: Albuterol inhaler as needed, OTC fish oil capsules, aspirin as needed

Allergies: No known drug allergies

Family history: Noncontributory

Social history: Social drinker; no other recreational drug use, never smoked cigarettes or used tobacco. No recent travel. Is employed as an attorney, married with children.

Re-examination shows patient is now awake, alert, and complaining only of mild headache. Repeat temperature is 37° C. Repeat neurologic exam shows normal gait, balance, and motor function, but with subtle dysdiadochokinesia in the left upper extremity. Monitor has continued to show normal sinus rhythm in the 80s.

The patient is sent for head CT, and the scan is shown. Which is the following is a correct interpretation of this CT scan? (Please choose only one answer.)

Dilated right lateral ventricle

Intraparenchymal hemorrhage in the right parietal area

Ischemic stroke in the right parietal area

Mild cerebral edema

© 2026 Rao AS et al. *JAMA Network Open*.

Question 5 | Type: **DX**

At this time, likely differential diagnoses now include which of the following? (You may select more than one option.)

Alcohol withdrawal

Cardiac arrhythmia

Intracerebral hemorrhage

Seizure

Subarachnoid hemorrhage

Thrombocytopenia

Vasovagal syncope

Question 6 | Type: **DIAG**

Given the previous findings, which of the following steps are most appropriate for guiding diagnosis and treatment? (You may select more than one option.)

No additional testing is needed; proceed to treatment

Bleeding time test

© 2026 Rao AS et al. *JAMA Network Open*.

Bone marrow biopsy

Coombs test

Hepatitis C testing

HIV testing

Liver function tests

Plasma fibrinogen and D-dimer levels

Peripheral blood smear

PT/PTT

Repeat physical examination of patient for signs of bleeding

Question 7 | Type: **DX**

Results from additional testing return and show the following:

Hepatitis serology panel:

© 2026 Rao AS et al. *JAMA Network Open*.

Normal
HIV testing:
Normal
Liver function tests:
Normal
Peripheral blood smear:
Thrombocytopenia, otherwise normal
PT/PTT:
Normal

Repeat examination for signs of bleeding shows petechiae present on both lower legs and an area of ecchymosis on the upper back. Stool is negative for occult blood, and the spleen is not palpably enlarged.

At this time, which of the following is the most likely diagnosis?

Bone marrow failure

Drug effects

Immune thrombocytopenia (ITP)

Sepsis

Von Willebrand disease (VWD)

Question 8 | Type: **MANG**

At this time, which of the following are part of the most appropriate management? (You may select more than one option.)

Admit the patient to the hospital

© 2026 Rao AS et al. *JAMA Network Open*.

Alteplase 0.9 mg/kg IV

Fresh frozen plasma 1 U IV

Immune globulin 1 g/kg IV

Lorazepam 2 mg IV

Phenytoin 18 mg/kg IV over 1 h

Platelet transfusion 6 U IV

Prednisone 1 mg/kg po

Prothrombin complex concentrate (PCC) 25 U/kg IV

Splenectomy

Question 9 | Type: Q

Which of the following features, if present, would suggest a dangerous cause of syncope? (You may select more than one option.)

Associated heart murmur

Onset of syncope during exertion

© 2026 Rao AS et al. *JAMA Network Open*.

Onset of syncope during strong emotional display

Onset of syncope immediately after standing up

Multiple recurrences of syncope within a short time

Syncope preceded by diaphoresis

Question 10 | Type: **Q**

Which of the following drugs are more often associated with development of thrombocytopenia? (You may select more than one option.)

Acetaminophen

Aspirin

Bivalirudin

Clopidogrel

Heparin

Quinine

© 2026 Rao AS et al. *JAMA Network Open*.

Ranitidine

Trimethoprim / sulfamethoxazole

Warfarin

Question 11 | Type: **Q**

Which of the following infections are associated with development of thrombocytopenia? (You may select more than one option.)

Epstein-Barr virus infection

Helicobacter pylori infection

Hepatitis C

HIV infection

Lyme disease

Syphilis

© 2026 Rao AS et al. *JAMA Network Open*.

Trichinosis

Question 12 | Type: **Q**

Which of the following types of stroke is least likely to cause loss of consciousness (LOC)?

Anterior circulation ischemic stroke

Embolic stroke

Posterior circulation stroke

Subarachnoid hemorrhage

© 2026 Rao AS et al. *JAMA Network Open*.

Case 18: Nausea and Increased Thirst in a 31-year-old woman

Background

History of Present Illness

31-year-old woman comes to the emergency department because of a 3-week history of nausea, reduced appetite, and increased thirst. She says that she is now drinking 3 to 4 liters of water per day with subsequent polyuria.

Review of Systems

General: Patient complains of lethargy and an increased thirst causing her to drink 3 to 4 liters of water per day. Her weight has not changed, and she has had no fever.

Skin: She reports mild generalized pruritus but no rash or lesions.

HEENT: Noncontributory

Pulmonary: She has no shortness of breath or cough.

Cardiovascular: Noncontributory

Gastrointestinal: Continuous nausea which is worse in the mornings but no vomiting.

Genitourinary: She passes urine every 1 to 2 hours including during night time. No dysuria or urinary urgency. Last normal menstrual period 3 weeks ago.

Musculoskeletal: No pain or swelling in joints or extremities, no back or neck pain.

© 2026 Rao AS et al. *JAMA Network Open*.

Neurologic: Mild generalized muscle weakness for 1 week. No difficulty seeing, speaking, swallowing, or walking. No numbness or paresthesia.

Psychiatric: Patient's husband reports the patient has recently developed labile mood with frequent episodes of depression and reduced concentration.

Past Medical History

Medical history: Primary hyperthyroidism due to Graves disease treated with radioactive iodine 6 years prior with subsequent hypothyroidism; depression 5 years prior that resolved with medication and counseling; no other psychiatric disorder. No known diabetes, pulmonary, or renal disease.

Surgical history: None

Medications: Levothyroxine 125 mcg daily, vitamin D 800 U daily.

Allergies: No known drug allergies.

Family history: Mother has type 2 diabetes mellitus and primary hypothyroidism.

Social history: Patient is married and has been a homemaker since the birth of her daughter 2 years ago. She used to work in an office. She is a nonsmoker, has no alcohol intake, and denies taking any recreational drugs. Her diet is healthy and balanced. She eats a moderate amount of dairy products, lean meat, fruit, and vegetables. She last traveled abroad 6 months ago to Portugal. She has not had any recent contact with anyone with febrile illness or GI symptoms.

© 2026 Rao AS et al. *JAMA Network Open*.

Physical Examination

General appearance: Well-appearing female with normal body habitus. She is carrying a bottle of water from which she sips during the evaluation.

Vital signs:

Temperature: 36.9¬∞ C

Pulse: 88 beats/min

Respirations 14/min, oxygen saturation 96%

BP 135/82 mm Hg

Skin: Normal fair skin.

HEENT: Unremarkable

Neck: Jugular venous pressure 5 cm above the clavicle at 45 degrees. Delayed carotid upstrokes.

Pulmonary: Bilateral and symmetric air entry; no rhonchi, crackles or wheezes.

Cardiovascular: Normal heart sounds, and jugular venous pressure is normal; no peripheral edema.

Gastrointestinal: Abdomen is soft, nontender on palpation; bowel sounds are normal. Rectal examination shows moderate amount of soft, brown, heme-negative stool.

Genitourinary: Unremarkable

© 2026 Rao AS et al. *JAMA Network Open*.

Musculoskeletal: Unremarkable

Neurologic: Alert and oriented. Muscle tone is generally normal, power is 5/5 in all muscles tested. Reflexes are 2/4 and symmetric in upper and lower extremities; plantar reflexes are downgoing.

Mental status: Patient reports a decreased mood but no suicidal thoughts.

Questions

Question 1 | Type: DIFF

At this time, which of the following diagnoses CANNOT be excluded based on history and physical examination? (You may select more than one option.)

Diabetes mellitus

Drug ingestion

Hypercalcemia

Hyperthyroidism

Hypothyroidism

Diabetes insipidus

Urinary tract infection

© 2026 Rao AS et al. *JAMA Network Open*.

Psychogenic polydipsia

Question 2 | Type: **DIAG**

Which of the following are the most appropriate studies to obtain at this time? (You may select more than one option.)

Complete blood count (CBC)

Random blood glucose

Serum C-reactive protein (CRP)

Serum electrolyte concentrations

Serum calcium and phosphate

Serum liver function studies (LFT)

Serum TSH and free T4

Serum urea and creatinine

Serum and urine osmolality

CT scan of the chest, abdomen, and pelvis

© 2026 Rao AS et al. *JAMA Network Open*.

Question 3 | Type: **DX**

Results of appropriate testing return and show the following:

Blood:

Test (units)

Result

Glucose, random

112 mg/dL

Serum:

Test (units)

Result

Calcium, total

13.6 mg/dL

Na+

136 mEq/L

K+

4.9 mEq/L

Cl-

95 mEq/L

HCO3-

22 mEq/L

Creatinine

0.8 mg/dL

TSH

0.45 mU/L

FT4

1.2 ng/dL [14 pmol/L]

Phosphate

1.9 mg/dL [0.6 mmol/L]

Osmolality

285 mOsmol/kg

Urine

Test (units)

Result

© 2026 Rao AS et al. *JAMA Network Open*.

Osmolality

210 mOsmol/kg

At this time, which of the differential diagnoses best account for the patient's findings? (You may select more than one option.)

Central diabetes insipidus

Diabetes mellitus

Hypercalcemia

Nephrogenic diabetes insipidus

Psychogenic polydipsia

Question 4 | Type: **Q**

Which of the following should be evaluated as possible causes of the patient's hypercalcemia? (You may select more than one option.)

Addison disease

Bone resorption due to malignant infiltration

Excessive calcium consumption

Familial hypocalciuric hypercalcemia

© 2026 Rao AS et al. *JAMA Network Open*.

Vitamin D toxicity

Primary hyperparathyroidism

Sarcoidosis

Secondary hyperparathyroidism due to chronic kidney disease

Paraneoplastic syndrome with cancer secreting PTH-related peptide

Question 5 | Type: **DIAG**

At this time, which of the following are the most appropriate studies to aid in establishing the diagnosis? (You may select more than one option.)

Serum angiotensin converting enzyme (ACE)

Serum IGF-1 level

Chest x-ray

Serum prolactin level

Serum total vitamin D level

© 2026 Rao AS et al. *JAMA Network Open*.

Ultrasonography of the neck

Urine calcium excretion ratio

24-hour urine calcium

Water deprivation test

Serum parathyroid hormone (PTH) level

MRI of the pituitary gland

Sestamibi scintigraphy scan of the neck

Serum and urine protein electrophoresis

Question 6 | Type: **Q**

🖼 **Image:** *18_Q_UT*

Results of appropriate studies return and show the following:

Serum:

Test (units)

Result

Protein electrophoresis

Normal

Parathyroid hormone (PTH)

© 2026 Rao AS et al. *JAMA Network Open*.

254 pg/mL, 26.9 pmol/L (normal 15-65 pg/mL, 1.6-6.9 pmol/L)
Total vitamin D
22.8 ng/mL, 57 nmol/L (normal 20-50 ng/mL, 50-125 nmol/L)
Angiotensin converting enzyme (ACE)
38 U/L, 633 nkat/L (normal <40 U/L, <670 nkat/L)

Urine:
Test (units)
Result
24-hour urine calcium
11 mmol/24 h (normal 2.5-7.5 mmol/24 h)
Urine calcium excretion ratio
0.09
Protein electrophoresis
Normal

Imaging:
Test (units)
Result
Ultrasonography of the neck
Nodule in right lower pole of thyroid
Chest x-ray
Normal

Results of technetium-99 sestamibi scan of the neck return and are shown.

Which of the following is a correct interpretation of the sestamibi scan? (Select only one option.)

Bilateral parotiditis

Increased uptake of the tracer by the right ectopic parathyroid adenoma

Increased uptake of the tracer by the right inferior parathyroid lesion

© 2026 Rao AS et al. *JAMA Network Open*.

Right toxic thyroid nodule

Toxic multinodular goiter

Question 7 | Type: **DX**

Which of the following is the most likely cause of hypercalcemia in this patient? (You may select more than one option.)

Ectopic parathyroid adenoma

Lymphoma

Multiple myeloma

Primary hyperparathyroidism due to right inferior parathyroid adenoma or cancer

Sarcoidosis

Secondary hyperparathyroidism due to vitamin D deficiency

Small-cell lung cancer secreting PTH-related peptide

Vitamin D toxicity

Question 8 | Type: **MANG**

© 2026 Rao AS et al. *JAMA Network Open*.

A diagnosis of hypercalcemia due to primary hyperparathyroidism likely due to right inferior parathyroid adenoma is confirmed. At this time, which of the following are most appropriate management steps for this patient? (You may select more than one option.)

Watch and wait, with regular monitoring of serum calcium levels and repeat neck imaging in 6 months

Desmopressin 50 mcg twice a day

Furosemide 80 mg twice daily

Prednisolone 40 mg once a day

Calcitonin 5 IU/kg subcutaneously twice a day subcutaneously

Intravenous 0.9% saline every 6 hours

Pamidronate 60 to 90 mg IV infusion

Hemofiltration

3-and-a-half glands parathyroidectomy

Right inferior parathyroidectomy

© 2026 Rao AS et al. *JAMA Network Open*.

Radiotherapy of the parathyroid lesion

Question 9 | Type: **Q**

Which of the following are symptoms of hypercalcemia? (You may select more than one option)

Abdominal pain

Confusion

Constipation

Depression

Diarrhea

Lethargy

Nausea and vomiting

Palpitations

Pruritus

© 2026 Rao AS et al. *JAMA Network Open*.

Polydipsia and polyuria

Reduced appetite

Tetany

Urinary retention

Question 10 | Type: **Q**

Which of the following are causes of hypercalcemia? (You may select more than one option)

Addison disease

Cushing syndrome

Hyperthyroidism

Hypomagnesemia

Hypothyroidism

Lymphoma

© 2026 Rao AS et al. *JAMA Network Open*.

Metastatic breast cancer

Metastatic prostate cancer

Milk-alkali syndrome

Multiple myeloma

Neuroendocrine tumor secreting PTH related peptide

Proton pump inhibitors

Small-cell lung carcinoma

Tuberculosis

Vitamin D intoxication

Question 11 | Type: Q

Which of the following are possible complications of parathyroidectomy? (You may select more than one option.)

Hypocalcemia

Hypoglycemia

© 2026 Rao AS et al. *JAMA Network Open*.

Hypokalemia

Hypothyroidism

Recurrent laryngeal nerve palsy

Tetany

© 2026 Rao AS et al. *JAMA Network Open*.

Case 19: Nipple discharge in a 31 yo female

Background

History of Present Illness

A 31-year-old woman comes to the office because of a 6-month history of nipple discharge. She has noticed that her breasts are tender and both nipples produce milky discharge on applying mild pressure. She has not noted any bloody or clear nipple discharge, breast lumps, or skin changes. She also states she has not had a menstrual period for 6 months, and her periods had been irregular for 8 months before they stopped altogether. Prior to her menstrual irregularities, her menses occurred at a normal frequency and duration. She is sexually active with a single partner and is trying to conceive.

Review of Systems

General: Gradually increasing lethargy over the past 2 months. 3 kg unintentional weight gain in the past 6 months.

Skin: No rash or history of excessive facial hair or acne.

HEENT: No rhinorrhea, tinnitus, vertigo, or change in hearing.

Pulmonary: Noncontributory

Cardiovascular: Noncontributory

Gastrointestinal: Noncontributory

Genitourinary: Noncontributory

© 2026 Rao AS et al. *JAMA Network Open*.

Gynecologic: Decreased libido. Vaginal dryness during sexual intercourse. She has been trying to conceive for the last 2 years. One full-term, uncomplicated pregnancy 5 years ago.

Musculoskeletal: Noncontributory

Neurologic: 6-month history of dull frontal and occasionally retro-orbital headaches that are increasing in frequency and that now occur almost daily and affect her work performance. There are no associated neurologic symptoms, including disturbances of vision, strength, balance, or gait. She denies nausea, photophobia, or phonophobia. Until 1 month ago, the headaches would resolve completely with nonsteroidal antiinflammatory drugs, but for the last month taking simple analgesia does not suffice. She denies history of headaches prior to 6 months ago.

Psychiatric: She is mildly depressed recently, which she attributes to the frequent headaches. No suicidal ideation.

Past Medical History

Medical history: She had mild asthma in childhood, which she outgrew. She has a 5-year-old son after an uncomplicated pregnancy with spontaneous vertex delivery at 40 weeks.

Surgical history: No previous operations.

Medications: Acetaminophen 1 g daily as needed; ibuprofen 400 mg daily as needed. Ibuprofen recently increased to as much as 400 to 600 mg twice daily.

Allergies: No known drug allergies.

© 2026 Rao AS et al. *JAMA Network Open*.

Family history: Her father had a stroke in his 80s; mother had osteoporosis.

Social history: She eats a healthy balanced diet. She does not exercise regularly. She is nonsmoker and drinks alcohol very occasionally. She works as a primary school teacher. Her last travel outside the country was to Italy 8 months ago.

Physical Examination

General appearance: Comfortable-appearing female who generally looks well and appears her stated age.

Vital signs:

BMI: 22 kg/m2

Temperature: 36.6¬∞ C

Pulse: 80 beats/minute and regular

Respirations: 18/min

BP: 135/74 mm Hg

Skin: Normal color, no rash, hirsutism, or acne.

HEENT: Pupils equal and reactive to light and accommodation. Normal visual fields to confrontation. Visual acuity 20/20 in both eyes. Fundi normal bilaterally. No thyroid enlargement or nodules.

Pulmonary: Equal air entry and chest is clear on auscultation. No crackles or wheezes.

© 2026 Rao AS et al. *JAMA Network Open*.

Cardiovascular: Regular rate and rhythm with normal heart sounds. No murmurs. Normal peripheral pulses and capillary refill.

GYN: No breast masses or skin changes. No axillary lymphadenopathy. Mild diffuse breast tenderness on palpation. Milky nipple discharge elicited bilaterally with pressure around areolae.

Gastrointestinal: Abdomen soft, nontender, and nondistended. No organomegaly, and bowel sounds are normal.

Genitourinary: Unremarkable

Musculoskeletal: Unremarkable

Neurologic: Normal and symmetric motor strength and reflexes on extremities. Sensation grossly intact to light touch. Cranial nerves 2 through 12 intact. Gait and balance normal.

Mental status: Alert, oriented to person, place, and time. Affect seems slightly depressed.

Questions

Question 1 | Type: DIFF

At this time, which of the following are common and/or dangerous differential diagnoses that cannot be excluded based on history and physical examination in this patient? (You may select more than one option.)

Breast cancer

End-stage renal disease (ESRD)

© 2026 Rao AS et al. *JAMA Network Open*.

Fibrocystic breast changes

Hypothalamic tumor

Physiologic nipple discharge

Pituitary tumor

Polycystic ovarian syndrome (PCOS)

Pregnancy

Primary hypothyroidism

Question 2 | Type: **DIAG**

Given the previous findings, which of the following are the most appropriate initial steps to guide diagnosis and treatment? (You may select more than one option.)

Serum β-hCG concentration

Serum free T4 level

© 2026 Rao AS et al. *JAMA Network Open*.

Serum prolactin (PRL) level

Serum thyroid-stimulating hormone (TSH)

Urine pregnancy test

CT scan of the brain without contrast

Mammography

MRI of the brain with and without contrast

Pelvic ultrasound

Ultrasound of the thyroid

Question 3 | Type: **DIAG**

Results of appropriate testing return and show the following (SI units are in parentheses):

Serum:

Test (units)

Result

Prolactin

500 µg/L

TSH

2.1 microIU/ml

© 2026 Rao AS et al. *JAMA Network Open*.

Urine pregnancy test: Negative

MRI brain with contrast: Slightly enlarged sella turcica, sellar mass measuring 1.1 cm x 1.6 cm x 2.1 cm, no optic chiasm compression, no hydrocephalus

Given the suspicion for pituitary tumor, which of the following steps are now most appropriate for guiding diagnosis and treatment? (You may select more than one option.)

Bilateral ocular tonometry

Humphrey's visual field test

Serum ACTH stimulation test

Serum AM cortisol level

Serum estradiol level

Serum FSH level

Serum free cortisol level

Serum free T4 level

Serum insulin-like growth factor 1 (IGF-1) level

© 2026 Rao AS et al. *JAMA Network Open*.

Serum LH level

Question 4 | Type: **DX**

Appropriate testing is obtained and the results are shown:

Humphrey's visual field test : Normal

Serum:

Test (units)

Result

LH

Normal

FSH

Normal

Free T4

Normal

Estradiol

Normal

IGF-1

Normal

AM cortisol

Normal

The most likely diagnosis in this patient is hyperprolactinemia due to:

Pituitary macroadenoma

Pituitary microadenoma

Pituitary stalk compression by nonfunctioning pituitary macroadenoma

Primary hypothyroidism

© 2026 Rao AS et al. *JAMA Network Open*.

Pregnancy

Question 5 | Type: **MANG**

A diagnosis of hyperprolactinemia due to pituitary macroadenoma is confirmed. At this time, which of the following is appropriate treatment for this patient? (You may select more than one option.)

Admit to the hospital for immediate transphenoidal surgical resection of pituitary tumor

Outpatient pituitary radiotherapy

Repeat MRI of the brain in 3 months and treat if tumor is becoming larger

Repeat serum prolactin level in 3 months and treat if serum prolactin level increases

Bromocriptine 2.5 mg orally twice a day

Cabergoline 1 mg orally 2 times per week

Lanreotide 90 mg subcutaneously 4 times per week

Pegvisomant 10 mg subcutaneously daily

Quinagolide 25 mcg orally once daily

© 2026 Rao AS et al. *JAMA Network Open*.

Question 6 | Type: **Q**

What are the most common complications of macroprolactinoma? (You may select more than one option.)

Bitemporal hemianopia

Cushing disease

Heart failure

Hirsutism

Hydrocephalus

Hypocalcemia

Infertility

Meningitis

Optic neuropathy

© 2026 Rao AS et al. *JAMA Network Open*.

Question 7 | Type: **Q**

What are the conditions most commonly associated with prolactinoma? (You may select more than one option.)

Carney complex

Cowden syndrome

Familiar hypocalcuric hypercalcemia

McCune-Albright syndrome

Multiple endocrine neoplasia Type 1 (MEN1)

POEMS syndrome

von Hippel-Lindau syndrome

Question 8 | Type: **Q**

Which drugs can cause hyperprolactinemia? (You may select more than one option.)

Haloperidol

Lisinopril

© 2026 Rao AS et al. *JAMA Network Open*.

Metoclopramide

Octreotide

Omeprazole

Ranitidine

Risperidone

Sildenafil

Simvastatin

Verapamil

© 2026 Rao AS et al. *JAMA Network Open*.

Case 20: Nocturia in a 70-yr-old man

Background

History of Present Illness

A 70-yr-old man comes to his internal medicine physician with a complaint of waking to urinate. This has been present for several years but has gradually increased in frequency from 2 times/night to 6 times/night. He is not urinating more during the day. He states that when he does urinate, he has to strain to initiate a stream and that his stream is "not like it was when he was 20 years old." He denies incontinence, dysuria, hematuria, flank pain, fevers, or any other complaints. He is otherwise relatively healthy and has not seen a physician in several years.

Review of Systems

General: Overall feels well, good appetite, no recent weight gain or loss, not drinking more fluids than usual.

Skin: Noncontributory

HEENT: Noncontributory

Pulmonary: Noncontributory

Cardiovascular: Denies palpitations or chest pain.

Gastrointestinal: No abdominal pain, nausea, vomiting, diarrhea, or constipation.

Genitourinary: See HPI.

Musculoskeletal: No pains in muscles, bones, or joints.

© 2026 Rao AS et al. *JAMA Network Open*.

Neurologic: Noncontributory

Psychiatric: Noncontributory

Past Medical History

Medical history: Atrial fibrillation diagnosed 6 mo ago, not taking anticoagulation drugs after informed discussion with cardiologist.

Surgical history: None

Medications: Avanafil 100 mg as needed; aspirin 325 mg daily.

Allergies: No known drug allergies.

Family history: His father died from prostate cancer at age 78.

Social history: The patient is a retired businessman who splits his time between sailing on a yacht and spending time with his grandchildren. He is active and exercises daily. He denies current or previous tobacco use. He has a glass of wine daily. He denies any illicit drug use. All relevant immunizations are up to date. He denies exposure to any sick contacts. He is sexually active with his wife of 35 yr. He recently returned from a sailing trip to the western Caribbean.

Physical Examination

General appearance: Appears young for stated age and is in no acute distress.

© 2026 Rao AS et al. *JAMA Network Open*.

Vital signs:
Temperature 37¬∞ C
Pulse: 80/min
BP: 125/62 mm Hg
Respirations: 16/min
Height:5'11" (1.8 m)
Weight: 160 lb (72.5 kg)
BMI: 22.3

Skin: Pink, warm, and dry to the touch. No rash.

HEENT: Normocephalic with no evidence of trauma.

Pulmonary: Breath sounds present and equal bilaterally. No stridor, rales, or wheezing.

Cardiovascular: Heart rate is irregularly irregular. Radial pulses are present and equal bilaterally. No murmurs, rubs, or gallops.

Gastrointestinal: Abdomen overall is soft, nontender to light and deep palpation. No guarding. No palpable bladder or suprapubic tenderness.

Genitourinary: Circumcised phallus, bilateral descended testes which are normal in texture and without pain on palpation. Digital rectal exam shows approximately 70-g prostate gland that is smooth and with no identifiable central sulcus. No nodules or tenderness.

Musculoskeletal: No costovertebral angle tenderness.

© 2026 Rao AS et al. *JAMA Network Open*.

Neurologic: Unremarkable

Mental status: Alert and oriented to person, place, and time.

*

Questions

Question 1 | Type: DIFF

At this time, the differential diagnoses should include which of the following? (You may select more than one option.)

Benign prostatic enlargement (also called benign prostatic hypertrophy [BPH])

Bladder stone

Chronic bacterial prostatitis

Detrusor-sphincter dyssynergia

Diabetes mellitus

Drug side effects

Prostate cancer

Urethral stricture

© 2026 Rao AS et al. *JAMA Network Open*.

Urinary tract infection

Question 2 | Type: **DIAG**

At this time, which of the following are the most appropriate next steps? (You may select more than one option.)

Post-void residual measurement

Prostate ultrasound

Serum creatinine concentration

Serum PSA level

Urinalysis and urine culture

Question 3 | Type: **DX**

Results of appropriate testing return and show the following results:

Urinalysis and urine culture: Negative for blood, glucose, ketones, bilirubin, leukocyte esterase, nitrites, protein and bacteria

Test (units)

Result

Serum PSA level

3.2 ng/ml [3.2 mcg/L]

At this time what is the most likely diagnosis? (You may select only one option.)

Benign prostatic hyperplasia (BPH)

Chronic bacterial prostatitis

© 2026 Rao AS et al. *JAMA Network Open*.

Prostate cancer

Bladder stone

Question 4 | Type: **MANG**

A diagnosis of BPH is confirmed. At this time, which of the following is the most appropriate management of this patient? (You may select more than one option.)

Doxazosin 1 mg po daily

Finasteride 5 mg po daily

Oxybutinin 5 mg po bid

Tadalafil 2.5 mg po daily

Prostate biopsy

Transurethral resection of the prostate

Question 5 | Type: **Q**

Which of the following are features of the American Urologic Association (AUA) symptom score? (You may select more than one option.)

Dysuria

Hematuria

© 2026 Rao AS et al. *JAMA Network Open*.

Nocturia

Incomplete emptying when urinating

Reduced quality of life

Urinary frequency

Urinary hesitancy

Urinary intermittence

Urinary urgency

Weak urinary stream

Question 6 | Type: **Q**

Which of the following drugs can worsen symptoms of benign prostatic hyperplasia? (You may select more than one option.)

Anticholinergics

Antihistamines

Bronchodilators

© 2026 Rao AS et al. *JAMA Network Open*.

NSAIDs

Opioids

Statins

Sympathomimetics

© 2026 Rao AS et al. *JAMA Network Open*.

Case 21: Recurrent headaches in a 31-yr-old woman

Background

History of Present Illness

A 31-yr-old woman comes to the emergency department because of a severe headache in the occipital region. She says the headache began suddenly about 1 h ago, and she describes it as "the worst headache of my life." She says the pain is constant and is accompanied by nausea but not vomiting. She denies visual symptoms, focal weakness, and problems with gait and balance.

She has frequently had similar headaches beginning about 2 yr ago. The headaches have increased in frequency this past week, and this episode is by far the worst. She says the headaches start suddenly, last about an hour, and are associated with palpitations, unexplained anxiety, and light-headedness. She has also had nausea and occasional swelling of the neck during these episodes. The headaches are usually associated with exertion or with straining during bowel movements but sometimes occur without any provocation. She has not been evaluated for these headaches before.

Review of Systems

General: She denies weight change and fevers but does feel generally fatigued.

Skin: Noncontributory

HEENT: She has noted occasional bilateral nontender swelling of the neck associated with the headaches. She denies any vision changes, lacrimation, nasal congestion or discharge, tinnitus, earache, discharge from the ear, sore throat, tooth pain, and facial flushing.

Pulmonary: Mild shortness of breath sometimes accompanies her palpitations and neck swelling, but she has no other respiratory symptoms.

© 2026 Rao AS et al. *JAMA Network Open*.

Cardiovascular: Patient reports palpitations that occur during headaches. Palpitations last from a few minutes to several hours and resolve spontaneously. She has no chest pain with the palpitations or at other times. Her exercise tolerance is normal when she is asymptomatic.

Gastrointestinal: Patient complains of nausea associated with the headaches but no vomiting. She denies abdominal pain, and her bowel movements have been normal except for one episode in which she felt as though she was going to faint while straining on the toilet.

Genitourinary: She has occasional left flank pain but no dysuria or hematuria. Last normal menstrual period was 2 wk ago; duration and flow were normal.

Musculoskeletal: She has no muscle aches, joint pain, or stiffness.

Neurologic: She reports a single episode of transient right-sided arm weakness and altered sensation that followed one of the headaches. The weakness lasted < 1 h and resolved completely. Patient denies photophobia, phonophobia, and neck pain. She has had no previous head or neck injury.

Psychiatric: She reports episodes of feeling suddenly anxious even when sitting relaxed in the evening.

Past Medical history

Medical history: Polycystic ovary syndrome with subfertility. Prehypertension: Patient was told her BP was 136/82 mm Hg at a routine physical examination 3 mo ago. At that time, she was asked to lose some weight and restrict salt intake.

Surgical history: Cesarean delivery 5 yr ago.

© 2026 Rao AS et al. *JAMA Network Open*.

Medications: Ibuprofen 400 mg tid as needed.

Allergies: Penicillin and erythromycin.

Family history: 3-yr-old sister died because of a kidney problem (details unknown to the patient). Two brothers are currently being treated for epilepsy. She has no family history of cardiovascular disease, heart attack, or cancer.

Social history: The patient has a healthy balanced diet, is a nonsmoker, and occasionally drinks alcohol. She denies any use of recreational drugs. She works in administration. She lives with her husband and two children. Her last foreign travel was 1 yr ago; it was a week-long holiday in Spain.

Physical Examination

General appearance: Woman who appears uncomfortable because of headache.

Vital signs:

Temperature: 37.6¬∞ C

Pulse: 104 beats/min

BP: 150/90 mm Hg

Respirations: 20/min

Oxygen saturation: 99% on room air

Skin: No pallor, rashes, or skin lesions.

© 2026 Rao AS et al. *JAMA Network Open*.

HEENT: Neck supple, no adenopathy. Scalp nontender, no focal swelling. Conjunctiva and sclera appear normal; no hemorrhage, exudate, or papilledema in fundi. Nasal passages clear. No erythema or exudate in pharynx.

Pulmonary: Chest is clear to auscultation bilaterally.

Cardiovascular: Jugular venous pressure is normal; heart rate is tachycardic and regular with normal heart sounds. No murmurs. Well-perfused peripheries with normal pulses and no peripheral edema.

Gastrointestinal: Soft with mild tenderness to palpation in epigastric area; patient states that the palpation worsened her palpitations. No guarding, rigidity, or rebound. Bowel sounds are normal.

Genitourinary: No costovertebral angle tenderness.

Musculoskeletal: Unremarkable

Neurologic: Alert, oriented to person, place, and time, and cooperating fully with examination. Pupils equal and reactive to light and accommodation. Remainder of cranial nerve examination also normal. Strength, sensation, and deep tendon reflexes normal and symmetric throughout. Gait not tested because of discomfort, but finger-nose and heel-shin test normal. Negative Kernig and Brudzinski signs.

Mental status: The patient appears anxious.

Questions

Question 1 | Type: DIFF

At this time, which of the following differential diagnoses cannot be excluded based on history and physical examination in this patient? (You may select more than one option.)

© 2026 Rao AS et al. *JAMA Network Open*.

Anxiety disorder

Brain tumor

Cluster headaches

Giant cell arteritis

Idiopathic intracranial hypertension

Meningitis

Migraine

Pheochromocytoma

Subarachnoid hemorrhage

Tension headache

Question 2 | Type: **DIAG**

Which of the following steps are essential to do immediately? (You may select more than one option.)

© 2026 Rao AS et al. *JAMA Network Open*.

Complete blood count (CBC)

Complete metabolic profile

CT scan of the head without IV contrast

CT scan of the head with IV contrast

Ibuprofen 600 mg po q 6 h and follow-up in the office in 24 h

MRI of the head

Question 3 | Type: **DX**

Image: *21_DX_CT*

CT scan of the head without contrast is obtained and is shown. Which of the following is a correct reading of the CT scan?

Crescent-shaped hyperdense collection overlying the left hemisphere

Effacement of cisterns and sulci

Hyperdense fluid collection in left sylvian fissure

Motion artifact

Normal head CT

© 2026 Rao AS et al. *JAMA Network Open*.

Question 4 | Type: **DX**

At this time, which of the following is the most likely diagnosis? (Please select only one option.)

Idiopathic intracranial hypertension

Migraine

Pheochromocytoma

Subarachnoid hemorrhage

Tension headache

Question 5 | Type: **MANG**

At this time, which of the following are part of appropriate management? (You may select more than one option.)

Admit the patient to the hospital

Discharge on hydrochlorothiazide 25 mg po once/day plus ramipril 5 mg po once/day

Furosemide 40 mg po bid

Ramipril 5 mg po once/day

© 2026 Rao AS et al. *JAMA Network Open*.

Propranolol 10 mg po q 6 h

Phenoxybenzamine 10 mg po tid

Morphine sulfate 5 mg IV q 4 h prn for headache

Ibuprofen 400 mg tid prn for headache

MRI of the abdomen and pelvis

^{123}I-MIBG (iodine-123-labeled metaiodobenzylguanidine) scintigraphy

Ultrasonography of the abdomen

Consult interventional radiology for tumor embolization

Question 6 | Type: **Q**

Which genetic conditions are more commonly associated with pheochromocytoma? (You may select more than one option.)

Carney complex

McCune-Albright disease

Multiple endocrine neoplasia type 1 (MEN1)

© 2026 Rao AS et al. *JAMA Network Open*.

Multiple endocrine neoplasia type 2 (MEN2)

Neurofibromatosis type 1 (NF1)

Succinate dehydrogenase subunit B-D (SDHB-D) mutations

Von Hippel-Lindau (VHL) disease

Question 7 | Type: **Q**

Which of the following drug classes are most likely to provoke a hypertensive crisis and therefore are contraindicated in patients with pheochromocytoma? (You may select more than one option.)

ACE inhibitors

Calcium channel blockers

Dopamine antagonists

Monoamine oxidase (MAO) inhibitors

NSAIDs

Opioids

© 2026 Rao AS et al. *JAMA Network Open*.

Tricyclic antidepressants

Question 8 | Type: **Q**

What are the most likely complications of pheochromocytoma? (You may select more than one option.)

Dilated cardiomyopathy

Gout

Hypertensive encephalopathy

Hypoglycemia

Hyponatremia

Left ventricular failure

Pulmonary edema

© 2026 Rao AS et al. *JAMA Network Open*.

Case 22: Right testicular mass in a 28-yo man

Background

History of Present Illness

A 28-yr-old man comes to the outpatient clinic for evaluation of a lump in his right testis. About 1 month ago, while taking a shower, he noted a hard area in his right testis that felt "like a marble." He had never noticed this before and decided to wait and see if anything would change. When the mass did not go away, he scheduled this appointment. Today, he states the lump is not painful or tender to the touch and has not changed in size. He has had annual physical exams and was never told that a lump was present. Four years ago, he had a scrotal ultrasound following a brief episode of scrotal discomfort after competing in a bicycle race; the result of the ultrasound was normal.

Review of Systems

General: Overall feels well. Denies any weight loss, fevers, or chills.

Skin: Denies rashes, itching, or other lesions.

HEENT: Noncontributory

Pulmonary: No cough or shortness of breath.

Cardiovascular: No palpitations or chest pain.

Gastrointestinal: Good appetite, bowel movements are normal. Denies nausea or vomiting. Denies abdominal or flank pain.

Genitourinary: No dysuria, hematuria, frequency, or urgency. No urethral discharge.

© 2026 Rao AS et al. *JAMA Network Open*.

Musculoskeletal: No back or joint pain, no leg swelling.

Neurologic: Noncontributory

Psychiatric: Noncontributory

Past Medical History

Medical history: No reported medical history. Denies history of urinary tract infections, difficulty with urination, immunosuppressive disorders, or sexually transmitted infections.

Surgical history: Rotator cuff repair 4 yr ago; left inguinal hernia repair as a child.

Medications: Occasional NSAIDs.

Allergies: No known drug allergies.

Family history: Mother and father healthy. No history of genitourinary malignancies in any known relatives.

Social history: He denies current or previous tobacco use. He exercises almost every day with a combination of weight training and cardio. He works primarily at a desk job with somewhat frequent travel. All relevant immunizations are up to date. He denies exposure to any sick contacts. He has had unprotected sex only with his wife for the past 3 yr.

© 2026 Rao AS et al. *JAMA Network Open*.

Physical Examination

General appearance: Thin male who appears fatigued and older than stated age; in no apparent respiratory distress.

Vital signs:

Temperature: 37¬∞ C

Pulse: 60 beats/min

BP: 112/65 mm Hg

Respirations: 16/min

Height: 6'

Weight: 180 lb

BMI: 24.4

Skin: Warm and dry to the touch.

HEENT: Nasopharynx appears normal; no neck masses or lymphadenopathy.

Pulmonary: Breath sounds present and equal bilaterally. No stridor, rales, or wheezing.

Cardiovascular: Heart rate is regular; radial and dorsalis pedis pulses are present and equal bilaterally; no pedal edema.

Gastrointestinal: Abdomen is not distended; there are no ecchymoses. Normal bowel sounds are present. Abdomen is soft and nontender to light and deep palpation; no masses are present.

© 2026 Rao AS et al. *JAMA Network Open*.

Genitourinary: Circumcised phallus, no visible lesions, bilateral descended testes. Left testis smooth and nontender. Left epididymis is normal in texture and nontender. Right testis has firm mass in the midsection that is nontender on palpation. Right epididymis is normal in texture and nontender. There are no palpable inguinal lymph nodes. No urethral discharge.

Musculoskeletal: No costovertebral angle tenderness.

Neurologic: Unremarkable

Mental status: Alert and oriented to person, place, and time.

Questions

Question 1 | Type: DIFF

At this time, the differential diagnosis should include which of the following? (You may select more than one option.)

Epididymitis/orchitis

Hematocele

Inguinal hernia

Spermatocele

Testicular abscess

© 2026 Rao AS et al. *JAMA Network Open*.

Testicular cancer

Testicular cyst

Testicular torsion

Varicocele

Question 2 | Type: **DIAG**

At this time, which of the following is the most appropriate next step in managing this patient? (You may select more than one option.)

Complete blood count (CBC)

Sexually transmitted disease (STD) panel

Urinalysis and culture

MRI of pelvis and scrotum

Scrotal ultrasound

Biopsy of testis

Question 3 | Type: **DX**

© 2026 Rao AS et al. *JAMA Network Open*.

🖼 **Image:** *22_DX_UT*

Appropriate testing is done and the result of the imaging study is shown. Which of the following is a correct interpretation of the scrotal ultrasonography?

Cystic testicular mass

Hydrocele

Inguinal hernia

Normal

Solid testicular mass

Question 4 | Type: **Q**

In this patient, which of the following is the most likely cause of a solid testicular mass?

Hydrocele

Inguinal hernia

Testicular cancer

Testicular cyst

Question 5 | Type: **MANG**

A diagnosis of testicular cancer is confirmed. At this time, which of the following are the most appropriate next steps? (You may select more than one option.)

© 2026 Rao AS et al. *JAMA Network Open*.

Discharge with repeat exam and ultrasonography in 4 wk

Urology consultation

Trans-scrotal orchiectomy

Bone scan

Chest x-ray

CT scan of the abdomen

CT scan of the chest

CBC

Serum alfa-fetoprotein level

Serum amylase level

Serum beta HCG level

Serum LDH level

© 2026 Rao AS et al. *JAMA Network Open*.

Serum testosterone level

Ciprofloxacin 500 mg po bid

Question 6 | Type: **Q**

What is the most common solid cancer in men age 14-35 yr? (You may select only one option.)

Bladder cancer

Colon cancer

Lung cancer

Prostate cancer

Testicular cancer

Question 7 | Type: **Q**

What is the most common complaint in patients presenting with a testicular mass? (You may select only one option.)

Hematospermia

Inguinal adenopathy

© 2026 Rao AS et al. *JAMA Network Open*.

Painless mass on testis

Scrotal erythema

Urethral discharge

Question 8 | Type: **Q**

Which of the following scrotal masses are expected to transilluminate on physical examination? (You may select more than one option.)

Hydrocele

Spermatocele

Testicular cancer

Varicocele

© 2026 Rao AS et al. *JAMA Network Open*.

Case 23: Scrotal pain in a 10-yr-old boy

Background

History of Present Illness

A 10-year-old boy is brought to the emergency department because of constant right-sided scrotal pain. The pain began several hours prior to presentation and awoke him from sleep. He is an otherwise healthy boy who states he has never experienced pain like this before. He denies a history of genital trauma or any sexual activity, and he has not noticed any swelling or lumps in his groin. He has not had any fevers, chills, urinary frequency, dysuria, hematuria, or urethral discharge.

Review of Systems

General: Denies subjective fevers or chills.

Skin: Denies itching and rashes or other lesions.

HEENT: Noncontributory

Pulmonary: Noncontributory

Cardiovascular: Denies palpitations, chest pain.

Gastrointestinal: Denies abdominal pain, diarrhea, constipation, nausea, or vomiting.

Genitourinary: No dysuria, hematuria, frequency, or urgency.

Musculoskeletal: Denies joint pain.

Neurologic: Denies numbness or paresthesias in any extremities.

© 2026 Rao AS et al. *JAMA Network Open*.

Psychiatric: Noncontributory

Past Medical History

Medical history: Eczema

Surgical history: Neonatal circumcision

Medications: Daily pediatric multivitamin

Allergies: No known drug allergies.

Family history: Grandfather with hypertension.

Social history: He is currently in 4th grade; immunizations are up-to-date; not exposed to tobacco smoke at home; no known sick contacts; has not traveled to any foreign countries.

Physical Examination

General appearance: In mild distress due to pain.

Vital signs:

Temperature: 36.7¬∞ C

Pulse: 87 beats/min

BP: 123/68 mm Hg

© 2026 Rao AS et al. *JAMA Network Open*.

Respirations: 16/min

Height: 137 cm

Weight: 32 kg

Skin: Warm and dry to the touch, no rashes.

HEENT: Normocephalic, atraumatic.

Pulmonary: Breath sounds present and equal bilaterally. No stridor, no rales, no wheezing.

Cardiovascular: Regular heart rate and rhythm, no murmurs, radial pulses are present and equal bilaterally.

Gastrointestinal: Abdomen is flat, no visible lesions, bowel sounds normal. On palpation, abdomen is soft, nontender, no guarding, no masses.

Genitourinary: Circumcised phallus, no urethral discharge or lesions, no scrotal swelling or erythema. Testes normal size, no masses.

Musculoskeletal: No costovertebral angle tenderness.

Neurologic: Unremarkable

Mental status: Alert and oriented to person, place, time, and event.

© 2026 Rao AS et al. *JAMA Network Open*.

Questions

Question 1 | Type: DIFF

At this time, the differential diagnoses should include which of the following? (You may select more than one option.)

Epididymitis

Incarcerated inguinal hernia

Nephrolithiasis

Orchitis

Testicular cancer

Testicular fracture

Testicular torsion

Torsion of the testicular appendage

Question 2 | Type: DIAG

Given the patient's history and physical exam findings, which of the following are the most appropriate tests to obtain in order to guide diagnosis and management? (You may select more than one option.)

© 2026 Rao AS et al. *JAMA Network Open*.

Basic metabolic profile (BMP)

Complete blood count (CBC)

Doppler ultrasonography of the scrotum

Serum erythrocyte sedimentation rate (ESR)

Urethral swab for sexually transmitted disease testing

Urinalysis and culture

Question 3 | Type: **DX**

Appropriate testing is ordered and results are shown:

Urinalysis: Normal

Doppler ultrasonography of the scrotum: 8 x 5 mm microcystic lesion adjacent to the right epididymal head. There is no significant flow within or surrounding the lesion. The testes appear normal bilaterally with appropriate arterial and venous flow.

At this time, which of the following is a correct diagnosis of this patient's condition? (Please select only one option.)

Epididymitis

Orchitis

© 2026 Rao AS et al. *JAMA Network Open*.

Testicular cancer

Testicular torsion

Torsion of the testicular appendage

Question 4 | Type: **MANG**

A diagnosis of torsion of the testicular appendage is confirmed. The most appropriate management of this patient's condition will include which of the following? (You many select more than one option.)

Admission to the hospital for surgical repair

Discharge with follow up in 3 days

Apply ice packs and prescribe supportive underwear

Prescribe ciprofloxacin 400 mg IV every 12 hours

Prescribe oral ibuprofen 300 mg every 6 hours

Prescribe oral ibuprofen 800 mg every 8 hours

Question 5 | Type: **Q**

In which stage of life is the incidence of torsion of an epididymal/testicular appendage greatest?

Perinatal

© 2026 Rao AS et al. *JAMA Network Open*.

Prepubertal

Adolescence

Adult

Geriatric

Question 6 | Type: **Q**

What is the embryologic origin of an epididymal appendage?

Mesonephric (wolffian) duct

Metanephros

Paramesonephric (müllerian) duct

Urachus

Question 7 | Type: **Q**

Which physical exam finding is most suggestive of a torsed epididymal/testicular appendage?

Blue dot sign

© 2026 Rao AS et al. *JAMA Network Open*.

Lack of sensation with palpation of scrotal skin

Palpable inguinal lymph nodes

Presence of a cremaster reflex

© 2026 Rao AS et al. *JAMA Network Open*.

Case 24: Scrotal pain in a 21 yo man

Background

History of Present Illness

A 21-year-old man comes to the emergency department because of a 3-day history of progressively worsening right scrotal pain. He says the pain started 3 days ago when he woke up. At first, the pain was a minor annoyance, but it has gradually increased in intensity to the point that it is now somewhat uncomfortable to walk. He has also noted a gradual swelling of his right scrotum but denies penile discharge, urinary frequency, dysuria, hematuria, or fevers. He denies any trauma to the area. He competed in a basketball game the previous evening but did not feel as though he strained himself during the game. He denies experiencing anything like this in the past. He has taken acetaminophen for the scrotal pain without any relief.

Review of Systems

General: Overall, has felt well. No unexplained weight loss; no fevers, chills, or rigors.

Skin: Denies rashes, itching, and other lesions.

HEENT: Sore throat a few days ago.

Pulmonary: Noncontributory

Cardiovascular: Denies palpitations and chest pain.

Gastrointestinal: No abdominal or flank pain; no change in bowel habits

Genitourinary: No dysuria, hematuria, frequency, or urgency. Right testicular pain and swelling. No urethral discharge.

© 2026 Rao AS et al. *JAMA Network Open*.

Musculoskeletal: Noncontributory

Neurologic: Noncontributory

Psychiatric: Noncontributory

Past Medical History

Medical history: None

Surgical history: Myringotomy tube placement as toddler.

Medications: Acetaminophen

Allergies: No known drug allergies.

Family history: Noncontributory

Social history: Patient is a student at the local community college. He denies current and previous tobacco use; he drinks a 6-pack of beer on weekends and denies illicit drug use. He exercises several times a week. All relevant immunizations are up-to-date. He denies any recent travel. He denies exposure to any sick contacts. He is sexually active with four partners and does not use condoms.

© 2026 Rao AS et al. *JAMA Network Open*.

Physical Examination

General appearance: Appears nontoxic but seems uncomfortable on the examination table

Vital signs:

Temperature: 37¬∞ C

Pulse: 80 beats/min

Respirations: 16/min

BP: 110/55 mm Hg

Height: 6'0"

Weight: 180 lb

BMI: 24.4

Skin: Warm and dry to the touch.

HEENT: Oropharynx is normal without erythema or exudate.

Pulmonary: Breath sounds present and equal bilaterally. No stridor, rales, or wheezing.

Cardiovascular: Regular heart rate and rhythm without murmurs; radial pulses present and equal bilaterally.

Gastrointestinal: Abdomen overall is soft and nontender to light and deep palpation. Normal bowel sounds. No guarding, no ecchymosis.

© 2026 Rao AS et al. *JAMA Network Open*.

Genitourinary: Circumcised phallus with visible swelling of the right hemiscrotum. Discomfort on palpation of the right testis and epididymis; left testis has normal texture and no pain on palpation. No tenderness or fullness over the inguinal canals. Cremasteric reflexes present bilaterally. No obvious urethral discharge.

Musculoskeletal: No costovertebral angle tenderness

Neurologic: Unremarkable

Mental status: Alert and oriented to person, place, time, and event.

Questions

Question 1 | Type: DIFF

At this time, which of the following differential diagnoses CANNOT be excluded based on history and physical examination in this patient? (You may select more than one option.)

Epididymitis or orchitis

Incarcerated inguinal hernia

Musculoskeletal strain or sprain

Nephrolithiasis

Testicular appendage torsion

© 2026 Rao AS et al. *JAMA Network Open*.

Testicular cancer

Testicular fracture

Testicular torsion

Question 2 | Type: **DIAG**

Given the previous findings, which of the following are the most appropriate steps to guide diagnosis and treatment? (You may select more than one option.)

Complete blood count (CBC)

Basic metabolic profile (BMP)

Sexually transmitted disease (STD) panel

Urinalysis (UA) and culture

MRI of pelvis and scrotum

Scrotal ultrasonography

Biopsy of testis

Question 3 | Type: **DX**

© 2026 Rao AS et al. *JAMA Network Open*.

Results of appropriate testing return and show the following results:

Urinalysis: Normal

Urine culture: Pending. Gram stain negative for bacteria.

STD panel: Pending

Testicular Doppler ultrasonography: Normal echogenicity of right testicle and epididymitis; significantly increased vascularity when compared to the left. Small, likely reactive right hydrocele.

Which of the following is the most likely diagnosis in this patient? (Please select only one option.)

Epididymitis or orchitis

Incarcerated inguinal hernia

Testicular appendage torsion

Testicular torsion

Testicular cancer

Question 4 | Type: **MANG**

At this time, which of the following are the most appropriate next steps? (You may select more than one option.)

© 2026 Rao AS et al. *JAMA Network Open*.

Ceftriaxone 250 mg IM

Doxycycline 100 mg po twice/day for 10 days

Await results of STD panel, then treat accordingly

Ibuprofen 400 mg po q 6 h as needed for pain

Acetaminophen 500 mg po q 6 h as needed for pain

Repeat testicular ultrasonography in 1 wk

Recommend condom usage every time he engages in sexual intercourse

Question 5 | Type: **Q**

Which of the following are common organisms associated with epididymitis in men <35 yr? (You may select more than one option.)

Chlamydia trachomatis

Escherichia coli

Klebsiella

Neisseria gonorrhoeae

© 2026 Rao AS et al. *JAMA Network Open*.

Proteus

Question 6 | Type: **Q**

Which of the following causes of epididymitis or orchitis are more common among immunocompromised patients? (You may select more than one option.)

Blastomycosis

Cytomegalovirus

Retrograde flow of urine

Syphilis

Tuberculosis

Question 7 | Type: **Q**

Which of the following commonly develop after mumps orchitis? (You may select more than one option.)

Infertility

Priapism

Testicular atrophy

© 2026 Rao AS et al. *JAMA Network Open*.

Testicular cancer

© 2026 Rao AS et al. *JAMA Network Open*.

Case 25: Shortness of breath in a 68 y/o man

Background

History of Present Illness

A 68-year-old man with a recent diagnosis of mild heart failure comes to the office because of a 1-week history of dyspnea on exertion. He is relatively sedentary but is usually able to climb a flight of stairs without stopping. Now he must stop every few steps to rest because of dyspnea but not chest discomfort. He also complains of lower extremity edema, 2-pillow orthopnea, cough with occasional production of white phlegm, wheezing, and a 3-kg weight gain. He denies chest pain or discomfort, palpitations, syncope or near syncope. He has had no fever or upper respiratory symptoms. He states he has been compliant with his medications and denies any changes to his diet. He was seen by another physician two times in the past few months for similar symptoms; both times he had an ECG and chest x-ray and was told he had "mild heart failure." He was treated with an increased dose of furosemide without any other workup.

Review of Systems

General: Patient feels generally fatigued. No fever, night sweats, or chills.

Skin: No rash, bruising, or other skin lesions.

HEENT: Sensation of fullness in the mouth. No vision changes, sore throat, or nasal congestion.

Pulmonary: See HPI

Cardiovascular: See HPI

Gastrointestinal: Mild decrease in appetite. No abdominal pain or change in bowel habits. No diarrhea. No dysphagia or odynophagia.

Genitourinary: Erectile dysfunction; no increased urinary frequency or urgency.

© 2026 Rao AS et al. *JAMA Network Open*.

Musculoskeletal: Bilateral painless swelling of both lower extremities below the knee for several weeks.

Neurologic: Occasional dizziness upon standing and an episode of syncope a few months ago. No focal weakness, numbness, or tingling in his extremities.

Psychiatric: Noncontributory

Past Medical History

Medical history: Coronary artery disease diagnosed 18 years ago as "small heart attack" that was treated with percutaneous coronary angioplasty (PTCA). Paroxysmal atrial fibrillation and mitral regurgitation diagnosed 1 year ago, dyslipidemia, rheumatoid arthritis (RA), and monoclonal gammopathy of undetermined significance (MGUS).

Surgical history: PTCA of the right coronary artery 18 years ago.

Medications: Aspirin 81 mg once a day, atorvastatin 40 mg once a day, warfarin 5 mg once a day, furosemide 40 mg once a day, nitroglycerin 0.4 mg sublingually as needed.

Allergies: No known drug allergies.

Family history: Father had hypertension and type 2 diabetes mellitus. Mother died of breast cancer. No family history of premature coronary artery disease or sudden cardiac death.

© 2026 Rao AS et al. *JAMA Network Open*.

Social history: Patient is retired and lives with his wife. He has been able to do routine activities of daily living and has not had recent prolonged immobilization. He does not exercise, and he tries to follow a low-sodium diet. He consumes 1 to 2 alcoholic drinks per week. He denies illicit drug or tobacco use. No recent travel outside the United States and no known sick exposures.

Physical Examination

General appearance: Thin male who appears fatigued and older than stated age; in no apparent respiratory distress.

Vital signs:

Temperature: 36.8¬∞ C

Pulse: 77 beats/min, regular

BP: 92/64 mm Hg

Respirations: 16/min

Pulse oximetry: 96% on room air

Skin: Cool, dry, soft. No rashes, cyanosis, petechiae, or lesions.

HEENT: Sclera anicteric. Mild macroglossia without surface discoloration or masses.

Neck: Normal carotid upstrokes. Jugular venous distention 12 cm above sternal notch at 45¬∞.

Pulmonary: Dullness to percussion and decreased breath sounds at the base bilaterally. Bibasilar crackles and faint end-expiratory wheezing.

© 2026 Rao AS et al. *JAMA Network Open*.

Cardiovascular: Regular rate and rhythm. S1 soft, S2 normal, S3 at the apex. 2/6 holosystolic murmur heard best at apex. PMI not displaced.

Gastrointestinal: Soft, nondistended, nontender, bowel sounds in all quadrants; liver nontender, span 15 cm.

Genitourinary: Unremarkable

Musculoskeletal: 2+ pitting lower extremity edema to the thighs bilaterally with no erythema, venous distention, warmth, or tenderness. Full range of motion.

Neurologic: Unremarkable

Mental status: Alert and oriented to person, place, and time.

Questions

Question 1 | Type: **DIFF**

At this time, based on history and physical examination, which of the following CANNOT be excluded as causes of the patient’s exertional dyspnea? (You may select more than one option.)

Acute decompensated heart failure (HF)

Acute myocardial infarction

Atrial fibrillation

© 2026 Rao AS et al. *JAMA Network Open*.

Cardiac tamponade

Constrictive pericarditis

COPD exacerbation

Pneumonia

Pneumothorax

Pulmonary embolism (PE)

Question 2 | Type: **DIAG**

At this time, which of the following are the most appropriate steps to help establish a diagnosis? (You may select more than one option.)

Arterial blood gas

Cardiac MRI

Chest x-ray (CXR)

CT scan of the chest

Electrocardiogram (ECG)

© 2026 Rao AS et al. *JAMA Network Open*.

Pulmonary function testing

Serum brain natriuretic peptide (BNP) level

Serum troponin level

Transthoracic echocardiography with Doppler ultrasonography (TTE)

Question 3 | Type: **DX**

Image: *25_DX_ECG*

Results of appropriate testing return and show the following results:

Test (units)

Result

Serum brain natriuretic peptide (BNP) level

1420 pg/mL

Serum troponin level

0.03 ng/mL

Chest x-ray: Perihilar opacities and small bilateral pleural effusions, no pericardial calcifications.

Transthoracic echocardiography: Normal left ventricular size and function with estimated LVEF of 55%. Severe concentric left ventricular hypertrophy. Normal right ventricular size and function. Restrictive LV diastolic pattern with E/A>2, septal e' of 7 cm/sec and lateral e' of 9 cm/sec. Biatrial enlargement. Mild mitral regurgitation. Inspiratory decrease of 5% in mitral diastolic flow velocity. No pericardial thickening.

ECG is also obtained and is shown below. This tracing is unchanged from prior ECG obtained 4 months ago.

Which of the following is a correct interpretation of this ECG?

Atrial fibrillation

© 2026 Rao AS et al. *JAMA Network Open*.

Sinus bradycardia with left bundle branch block (LBBB)

Sinus rhythm with LBBB and 1st degree AV block

Sinus rhythm with RBBB and 1st degree AV block

Question 4 | Type: **DX**

At this time, which of the following is the most likely diagnosis? (Please select only one option.)

Acute decompensated heart failure

Acute Myocardial infarction (MI)

Cardiac tamponade

Constrictive pericarditis

Question 5 | Type: **DIAG**

A diagnosis of acute decompensated heart failure (ADHF) is confirmed. Given the previous findings, which of the following steps are now most appropriate for guiding diagnosis and treatment? (You may select more than one option.)

Cardiac catheterization

Dobutamine stress echocardiography

© 2026 Rao AS et al. *JAMA Network Open*.

Exercise stress test

Transesophageal echocardiogram (TEE)

Question 6 | Type: **Q**

Results from additional testing return and show:

Cardiac catheterization: Nonobstructive coronary artery disease.

LVEDP 18 mm Hg

RAP 12 mm Hg

RVESP 50 mm Hg

RVEDP 11 mm Hg

PASP 36 mm Hg

PCWP 17 mm Hg

Prominent y descent is present on RA waveform. Concordance in LV and RV pressure tracings with respiration.

At this time which of the following is the most likely etiology of the patient's acute decompensated heart failure (ADHF)? (You may select only one option.)

Dilated cardiomyopathy

Hypertensive cardiomyopathy

Restrictive cardiomyopathy

Valvular cardiomyopathy

Question 7 | Type: **DIAG**

A diagnosis of restrictive cardiomyopathy is established. Given the test findings, which of the following steps are now most appropriate to determine the etiology of this patient's restrictive cardiomyopathy? (You may select more than one option.)

© 2026 Rao AS et al. *JAMA Network Open*.

Complete blood count (CBC) with differential

Endomyocardial biopsy

Fat pad biopsy

Serum ferritin level

Question 8 | Type: **Q**

Results of appropriate testing return and are shown:

Test (units)

Result

CBC with differential

Normal

Serum ferritin

100 ng/mL

Fat pad biopsy: Amorphous extracellular pink deposits with hematoxylin and eosin stain and apple-green birefringence under polarized microscopy on Congo red staining.

At this time, which of the following is the most likely etiology of the patient’s restrictive cardiomyopathy? (You may select only one option.)

Restrictive cardiomyopathy due to cardiac amyloidosis

Restrictive cardiomyopathy due to cardiac sarcoidosis

© 2026 Rao AS et al. *JAMA Network Open*.

Restrictive cardiomyopathy due to hemochromatosis

Restrictive cardiomyopathy due to hypereosinophilic syndrome

Question 9 | Type: **MANG**

At this time, his management should include which of the following? (You may select more than one option.)

Discharge home with instructions to return if symptoms worsen

Admit to a monitored telemetry inpatient unit for further treatment

2-g sodium restriction

1-L fluid restriction

Antiplasma cell therapy

Furosemide 40 mg IV bolus followed by 10 mg/hour furosemide infusion

Furosemide 40 mg orally

Digoxin 125 mcg orally daily

Dobutamine 2 mcg/kg/minute

© 2026 Rao AS et al. *JAMA Network Open*.

Lisinopril 20 mg orally daily

Metoprolol 50 mg orally daily

Amyloid typing

Question 10 | Type: **Q**

Which of the following drugs have been shown to decrease mortality in patients with heart failure with reduced ejection fraction? (You may select more than one option.)

ACE inhibitors

Aldosterone antagonists

Angiotensin II receptor blockers

Beta blockers

Digoxin

Loop diuretics

Nitrates

Question 11 | Type: **Q**

Which of the following factors suggest candidacy for cardiac resynchronization therapy in heart failure? (You may select more than one option.)

© 2026 Rao AS et al. *JAMA Network Open*.

Left ventricular ejection fraction <35%

Left ventricular end-diastolic dimension ≥55 mm

New York Heart Association (NYHA) class I symptoms

Nonspecific intraventricular conduction delay with mildly prolonged QRS

Permanent atrial fibrillation

QRS >130 msec

Question 12 | Type: **Q**

Which of the following are common findings in patients with amyloid cardiomyopathy? (You may select more than one option.)

Aortic stenosis

Arrhythmias

Dilated cardiomyopathy

Diastolic dysfunction

© 2026 Rao AS et al. *JAMA Network Open*.

Heart block

Heart failure

Hypertension

Question 13 | Type: **Q**

Which of the following may be effective in the treatment of light-chain (AL) cardiac amyloidosis? (You may select more than one option.)

ACE inhibitors

Antiplasma cell therapy

Autologous stem cell transplantation

Beta blockers

Calcium channel blockers

Heart transplantation

© 2026 Rao AS et al. *JAMA Network Open*.

Case 26: Syncope in an 80-Yr Old Woman

Background

History of Present Illness

An 80-yr-old woman is brought to the emergency department by emergency medical services (EMS) personnel because of a syncopal episode at home. She had been in the shower, suddenly felt weak, and collapsed to the floor. Her husband heard her fall and found her awake, lying on the bathroom floor. He called EMS who, when they arrived in the patient‚Äôs home, found her to be tachycardic and hypotensive. On arrival in the emergency department, she is confused and has difficulty answering questions. Her husband states she has hypertension, dyslipidemia, and mild dementia and, 1 wk ago, had a transcatheter aortic valve replacement for severe aortic stenosis. He was told the procedure had been uncomplicated and states that his wife had been feeling well during her recovery. She has never had an event like this one before.

Review of Systems

* General: The patient had been feeling fine at home until today. She felt light-headed in the shower before her episode.

*

Skin: She denies any rashes. She has bruising on her abdomen where she received injections for venous thromboembolism prophylaxis during her recent hospitalization. Her right groin incision site has not been painful, red, or purulent.

*

HEENT: Noncontributory

*

Pulmonary: Dyspnea at rest.

*

Cardiovascular: Constant, midsternal chest pain and pressure that does not radiate. No palpitations before or after this morning‚Äôs event.

*

Gastrointestinal: Noncontributory

*

Genitourinary: Noncontributory

*

Musculoskeletal: Denies pain in arms or legs.

*

Neurologic: Feels light-headed with standing. No vision changes, focal weakness, or other neurologic symptoms.

© 2026 Rao AS et al. *JAMA Network Open*.

*

Psychiatric: History of mild dementia with intermittent forgetfulness. Her husband states that her dementia did not seem worse before today,Äôs event but she now seems confused.

Past Medical History

* Medical history: Hypertension, hyperlipidemia, and aortic stenosis (severe).

*

Surgical history: Transcatheter aortic valve replacement 1-wk prior.

*

Medications: Clopidogrel, aspirin, docusate, multivitamins, and estrogen cream.

*

Allergies: No known drug allergies.

*

Family history: Noncontributory

*

Social history: Limited exercise tolerance. No recent travel. No history of drug or alcohol abuse.

Physical Examination

* General appearance: Ill-appearing, confused elderly woman in respiratory distress.

*

Vital signs:

* Temperature: 34.6¬∞ C
* Pulse: 115 beats/min, regular
* BP: 70/21 mmHg
* Respirations: 34/min

*

Skin: Face plethoric, cyanotic, and mottled. Extremities mottled. Multiple small ecchymoses on lower abdomen compatible with history of recent anticoagulant injections. Groin incision healing well, no bleeding.

*

HEENT: Significant bilateral jugular venous distention to angle of jaw.

*

Pulmonary: Shallow, rapid respirations; lung sounds clear and equal bilaterally; normal to percussion bilaterally.

*

© 2026 Rao AS et al. *JAMA Network Open*.

Cardiovascular: Tachycardia, mid-systolic mechanical click consistent with mechanical aortic valve. Heart sounds muffled. Femoral and carotid pulses weak but symmetric. Unable to palpate more distal pulses. Chest nontender, no crepitance with palpation.

*

Gastrointestinal: Unremarkable, including rectal exam showing stool brown and guaiac-negative.

*

Genitourinary: Unremarkable

*

Musculoskeletal: No swelling, erythema, tenderness, or deformities of extremities.

*

Neurologic: Patient is unable to cooperate with detailed examination, but pupils are symmetric and react equally to light, face appears symmetric, gag reflex is normal, and all extremities move equally on command.

*

Mental status: Oriented only to name. Appears confused.

Questions

Question 1 | Type: DIFF

At this time, which of the following differential diagnoses cannot be excluded based on history and physical examination in this patient? (You may select more than one option.)

Acute coronary syndrome/ST segment elevation myocardial infarction (STEMI)

Aortic dissection

Dehydration

Dysrhythmia

Hypoglycemia

© 2026 Rao AS et al. *JAMA Network Open*.

Neurogenic shock

Pericardial effusion with cardiac tamponade

Pulmonary embolism (PE)

Septic shock

Stroke/transient ischemic attack (TIA)

Tension pneumothorax

Vasovagal syncope

Question 2 | Type: **MANG**

Which of the following steps are essential to do immediately? (You may select more than one option.)

Bedside glucose measurement

Toxicology screening

BP measurement every 5 min

© 2026 Rao AS et al. *JAMA Network Open*.

Pulse oximetry

Cardiac monitoring

Oxygen 15 L by nonrebreather mask

0.9% saline solution, 1 L IV bolus

Dextrose 50 g IV

Naloxone 0.4 mg IV

Question 3 | Type: **DIAG**

Results of appropriate testing return and show the following:

Bedside glucose level: 72 mg/dL

Pulse oximetry: 85% on room air; increases to 92% after giving oxygen

BP improves to 80/35 mm Hg with administration of 1 L fluid bolus.

Given the previous findings, which of the following steps are now most appropriate for guiding diagnosis and treatment?

ECG

Echocardiography at bedside

© 2026 Rao AS et al. *JAMA Network Open*.

Echocardiography in a radiology suite

Chest x-ray at bedside

CT scan of the chest

CT scan of the head

Question 4 | Type: **DX**

🖼 **Image:** *26_DX_ECG*

Results of appropriate testing return and show the following:

Chest x-ray: Slight enlargement of the cardiac silhouette, otherwise unremarkable

ECG: Sinus tachycardia, normal voltage, no ST-T wave abnormalities, no electrical alternans

Results of echocardiography are shown below. Which of the following is the correct interpretation of this echocardiogram? (Please select one choice.)

Dilated right ventricle

Normal

Pericardial effusion

Pericardial thickening

Question 5 | Type: **DX**

At this time, what is the most likely diagnosis? (Please select one option.)

© 2026 Rao AS et al. *JAMA Network Open*.

Acute coronary syndrome

Cardiac tamponade

Dysrhythmia

Pulmonary embolism

Question 6 | Type: **MANG**

A diagnosis of cardiac tamponade is confirmed.

At this time, which of the following are the most appropriate next steps? (You may select more than one option.)

Consult cardiothoracic surgeon

0.9% saline solution, 1 L IV bolus

Heparin 5000 U IV

Pericardiocentesis, done at bedside

Pericardiocentesis, done in an operating room

Question 7 | Type: **Q**

Pulsus paradoxus is often associated with which of the following conditions? (You may select more than one option.)

Asthma exacerbation

© 2026 Rao AS et al. *JAMA Network Open*.

Cardiac tamponade

Congestive heart failure

Pulmonary embolism

Question 8 | Type: **Q**

Which of the following findings are commonly associated with cardiac tamponade? (You may select more than one option.)

Electrical alternans

Hypotension

Jugular venous distention

Muffled heart sounds

Tachycardia

Question 9 | Type: **Q**

Which ECG findings are classically associated with pericardial effusion and cardiac tamponade? (You may select more than one option.)

Electrical alternans (beat-to-beat QRS amplitude variability

Left ventricular hypertrophy

S_1, Q3, and T3

Tachycardia

© 2026 Rao AS et al. *JAMA Network Open*.

Case 27: Weight Gain and Muscle Weakness in 54-year old man

Background

History of Present Illness

A 54-year-old man comes to the office because of a 10-kg weight gain in the past 3 months despite no significant change in his diet. He also reports bilateral leg weakness, especially when standing up from sitting or when climbing stairs. On questioning, he complains of lethargy and difficulty in getting up in the morning despite getting 8 hours of sleep. He was recently diagnosed with type 2 diabetes and was started on metformin; however, good glycemic control has not been achieved. At a visit 2 months ago, he also complained of low mood for which citalopram was prescribed but with no significant improvement. He tells you today that his wife has left him because she could not tolerate his lack of motivation and mood swings.

Review of Systems

* General: 10-kg weight gain despite no change in appetite. Complains of increased thirst and urination; no fever.

*

Skin: Recent onset acne on the upper back, new stretch marks on the lower abdomen, mild chronic pruritus.

*

HEENT: Noncontributory

*

Pulmonary: Mild exertional shortness of breath, no cough.

*

Cardiovascular: Occasional left-sided chest pain on exertion that resolves at rest; no palpitations.

*

Gastrointestinal: No nausea or vomiting, bowel movement once daily with normal stool.

*

Genitourinary: Noncontributory

*

Musculoskeletal: Bilateral painless leg swelling

*

Neurologic: Patient complains of worsening short-term memory for the past month or two; he forgot his doctor appointment twice recently. Also notes bilateral leg weakness especially noted

© 2026 Rao AS et al. *JAMA Network Open*.

when standing up from sitting or climbing stairs. No headaches, visual symptoms or sensory changes.

*

Psychiatric: Low mood for the past 2 months with no significant change since starting citalopram. He is asking you to authorize 2-weeks of sick leave as he struggles at work due to poor concentration.

Past Medical History

* Medical history: Ulcerative colitis for 30 years, controlled on medications, primary biliary cirrhosis, depression, type 2 diabetes (recent diagnosis). No history of hypertension or cardiac disease.

*

Surgical history: Pyloric stenosis surgically repaired as a neonate.

*

Medications: Citalopram 20 mg once a day, mesalazine 1 g twice a day, ranitidine 150 mg twice a day, ursodeoxycholic acid 500 mg twice a day, metformin 500 mg three times a day.

*

Allergies: Previous tongue swelling after lymecycline.

*

Family history: Not known (adopted).

*

Social history: Patient works as a community psychiatric nurse, is married but recently separated due to his mood swings. He is a nonsmoker, has no alcohol intake, no recreational drugs use. No recent travel abroad.

Physical Examination

* General appearance: Slightly unkempt. Has truncal adipose tissue distribution with relatively thin legs, a moon-like face, and posterior cervical fat pad.

*

Vital signs:

* Temperature: 36.8¬∞ C
* Pulse: 72 beats/min
* Respirations: 18/min
* BP: 188/92 mm Hg
*

© 2026 Rao AS et al. *JAMA Network Open*.

Skin: Skin is generally thin; there is acne on the upper thorax and wide purple striae on the lower abdomen and around the axillae. There are a few bruises on the legs and forearms. No obvious pallor or jaundice. Hair is thin and sparse with temporal balding.

*

HEENT: On examination of eye movements, left eye is deviated down and laterally with mild ptosis and left pupil is 1 mm bigger than the right and weakly reacting to light. Patient reports double vision on looking to the right.

*

Pulmonary: Bilateral and symmetric air entry, a few bibasal end-inspiratory crackles on auscultation of the lungs.

*

Cardiovascular: Normal heart sounds, no murmurs, rubs, or gallops. Jugular venous pulse visible but not elevated. Bilateral moderate pitting leg edema. The dorsalis pedis pulses are difficult to palpate due to leg edema.

*

Gastrointestinal: There is an old surgical scar in the epigastric area. Abdomen is soft, nontender on palpation and bowel sounds are normal. Rectal normal, stool heme negative.

*

Genitourinary: Unremarkable

*

Musculoskeletal: Slim legs and arms except for visible bilateral lower leg edema that is not red or tender. No muscle tenderness or fasciculations.

*

Neurologic: Alert and oriented. Muscle tone is generally normal, Strength 5/5 and symmetric except for weakness of quadriceps 4+/5 bilaterally; weakness is constant and does not increase following use of the quadriceps during testing. Reflexes are brisk and symmetric in upper and lower limbs; plantar reflexes are down-going.

*

Mental status: Patient reports a decreased mood but no suicidal thoughts.

Questions

Question 1 | Type: Q

This patient's eye examination most likely represents an abnormality of which neurological structure? (You may select more than one option.)

Cranial nerve II

© 2026 Rao AS et al. *JAMA Network Open*.

Cranial nerve III

Cranial nerve IV

Cranial nerve VI

Question 2 | Type: **DIFF**

At this time, which of the following differential diagnoses cannot be excluded based on history and physical examination in this patient? (You may select more than one option.)

Brain tumor

Cavernous sinus thrombosis

Cushing syndrome

Diabetes, uncontrolled

Hypothyroidism

Myasthenia gravis

Muscular dystrophy

Polymyalgia rheumatica

Polymyositis or dermatomyositis

Question 3 | Type: **DIAG**

At this time, which of the following are the most appropriate next steps? (You may select more than one option.)

Blood glucose

Blood ketones

Complete blood count (CBC)

© 2026 Rao AS et al. *JAMA Network Open*.

Midnight serum cortisol

MRI of the cranium

Serum acetylcholine receptor antibody levels

Serum electrolyte concentrations

Serum free T4 (FT4)

Serum lactate dehydrogenase (LDH)

Serum liver function studies (LFT)

Serum parathyroid hormone level (PTH)

Serum TSH

Serum and urine protein electrophoresis

Question 4 | Type: **DX**

🖼 **Image:** *17_DX_MRI1, 17_DX_MRI2*

Results of appropriate testing return and show the following:

Blood:

Test (units)

Result (normal value)

Results (SI Units)

Glucose

306 mg/dL

17.0 mmol/L

Capillary Blood Ketones

Negative

© 2026 Rao AS et al. *JAMA Network Open*.

Serum:

Test (units)

Result (normal value)

Results (SI Units)

Na+

138 mEq/L

138 mmol/L

K+

2.9 mEq/L

2.9 mmol/L

Cl-

95 mEq/L

95 mmol/L

HCO3-

28 mEq/L

28 mmol/L

Creatinine

0.88 mg/dL

78 mcmol/L

Midnight serum cortisol

43.5 mcg/dL

1200 nmol/L

LFT

Normal

TSH

0.04 mIU/L

0.04 mIU/L

FT4

0.65 ng/dL

8.4 pmol/L

Results of MRI of the pituitary gland return and are shown:

© 2026 Rao AS et al. *JAMA Network Open*.

Which of the following is a correct interpretation of the MRI of the pituitary gland? (Please select only one option.)

Central pontine myelinosis

Intraventricular bleed into 3rd and 4th ventricle

Pituitary macroadenoma with left cavernous sinus invasion; central necrotic component

Pituitary microadenoma in the left cavernous sinus

Suprasellar meningioma with left cavernous sinus involvement

Question 5 | Type: **DX**

At this time, which single diagnosis best accounts for the patient's findings? (Please select only one option.)

Cushing syndrome

Diabetes, uncontrolled

Hypokalemia

© 2026 Rao AS et al. *JAMA Network Open*.

Hypothyroidism

Question 6 | Type: **MANG**

A diagnosis of Cushing syndrome is confirmed. At this time, which of the following are the most appropriate next steps? (You may select more than one option.)

Chest x-ray

Inferior petrosal sinus sampling for serum ACTH and cortisol levels

Oral glucose tolerance test

Serum ACTH level

Serum corticotropin-releasing hormone (CRH) test

Serum LH level

Serum prolactin level

Serum testosterone level

Question 7 | Type: **Q**

Results of appropriate studies return and show the following:

Serum:

Test (units)

Result

Results (SI Units)

ACTH

188 pg/mL [41.4 pmol/L]

Normal 10-60 pg/mL [2.2-13.2 pmol/L]

Testosterone

78 ng/dL [2.7 nmol/L]

Normal 300-1200 ng/dL [10-42 nmol/L]

Cortisol

36.6 mcg/dL [1010 nmol/L] to 52.6 mcg/dL [1450 nmol/L]

© 2026 Rao AS et al. *JAMA Network Open*.

ACTH
122 pg/mL [26.8 pmol/L] to 167 pg/mL [36.7 pmol/L]

LH
0.4 mU/mL [0.4 IU/L]
Normal 3-15 mU/mL [3-15 U/L]
FSH
0.9 mU/mL [0.9 IU/L]
Normal 5-15 mU/mL [5-15 IU/L]
Prolactin
5.6 ng/mL [245 pmol/L]
Normal < 15 ng/mL [< 652 pmol/L]

Chest x-ray: Reported as unremarkable

A diagnosis of Cushing syndrome (CS) is confirmed. Which of the following is the most likely cause of CS in this patient? (Please select only one option.)

Corticosteroid-secreting adrenal tumor

Ectopic ACTH secretion

Iatrogenic corticosteroid use

Pituitary ACTH secretion (Cushing's disease)

Question 8 | Type: **MANG**

A diagnosis of pituitary ACTH-dependent Cushing syndrome is confirmed. At this time, which of the following are appropriate management steps for this patient? (You may select more than one option.)

Cabergoline 0.5 mg once a week

© 2026 Rao AS et al. *JAMA Network Open*.

Desmopressin 50 mcg twice a day

Furosemide 80 mg twice a day

Metyrapone 250 orally 4 times a day

Pasireotide 600 mg once a day as monotherapy

Pegvisomant 40 mg once a day

Ramipril 10 mg once a day

Treat acne with lymecycline 408 mg once a day

Bilateral adrenalectomy

Transsphenoidal adenomectomy

Watch and wait, with regular monitoring of serum cortisol levels and repeat MRI of the pituitary yearly

Question 9 | Type: **Q**

Which of the following are the complications of Cushing disease? (You may select more than one option.)

III, IV, or/and VI cranial nerve palsy

IX to XII cranial nerve palsy

Bitemporal hemianopia due to an optic chiasm compression

Colonic polyps

Deep vein thrombosis

Hypoglycemia

© 2026 Rao AS et al. *JAMA Network Open*.

Pituitary tumor apoplexy

Sepsis

Tardive dyskinesia

Question 10 | Type: **Q**

Which of the following are possible causes of an ectopic ACTH-dependent CS? (You may select more than one option.)

Breast cancer

Endometrial carcinoma

Lymphoma

Medullary thyroid carcinoma

Neuroendocrine tumor

Prostate cancer

Small-cell lung carcinoma

Thymoma

Question 11 | Type: **Q**

Which of the following are first-line (screening) tests for confirmation of endogenous hypercortisolism in patients with suspected CS? (You may select more than one option.)

At least two 24-hour collections of urinary free cortisol

Clonidine challenge test

Inferior petrosal sinus sampling

© 2026 Rao AS et al. *JAMA Network Open*.

Midnight sleeping serum or salivary cortisol

Overnight dexamethasone suppression test

Serum CRH test

Question 12 | Type: **MANG**

Which of the following are treatment options for patients with Cushing disease? (You may select more than one option.)

Bilateral adrenalectomy

Metyrapone or ketoconazole

Norditropin

Pegvisomont

Phenoxybenzamine

Pituitary radiotherapy

Transsphenoidal pituitary adenomectomy

© 2026 Rao AS et al. *JAMA Network Open*.

Case 28: Weight Loss and Dizziness in a 29 year old man

Background

History of Present Illness

A 29-year-old man comes to the office because he has had weight loss, lethargy, and postural dizziness for the past 2 months. He says his symptoms began just after surgery to repair a diaphragmatic hernia caused by a stab wound to the chest 6 months ago. He first went to the emergency department 3 weeks after surgery because of sharp right-sided chest pain. At that time, CT scan of the chest was obtained to rule out pulmonary embolism, which was suspected because he was found to have low blood pressure along with his chest pain. No evidence of pulmonary embolism was noted on CT scan. He was discharged home on an antiemetic and acetaminophen. Today he says that, since the operation, he has had gradually worsening lethargy, morning nausea with occasional vomiting, and generalized abdominal pain. He feels very dizzy when he stands up and has lost 12 kg in the last 2 months.

Review of Systems

* General: 12-kg weight loss and slightly decreased appetite. No fever or chills.

*

Skin: Recent onset darkening of his nipples and the knuckles of his hands. No rash or lesions.

*

HEENT: Noncontributory

*

Pulmonary: Mild exertional shortness of breath, which is new. No cough.

*

Cardiovascular: Occasional palpitations and lightheadedness on standing up, which resolves after a few minutes. No chest pain, no peripheral edema.

*

Gastrointestinal: Bowel movement once daily with normal stool, morning nausea with occasional non-bloody vomiting, and generalized mild abdominal pain that is unrelated to meals, position, or activity.

*

Genitourinary: No dysuria or increased urinary frequency or volume.

*

Musculoskeletal: Generalized mild weakness; he struggles to get through a day,Äôs work as a builder. No swelling or pain in legs, joints, or muscles.

© 2026 Rao AS et al. *JAMA Network Open*.

*

Neurologic: Noncontributory

*

Psychiatric: Low mood for the last 2 months due to tiredness.

Past Medical History

* Medical history: Seasonal allergies, recent mild depression.

*

Surgical history: Stab wound to left side of chest 6 months prior and subsequent left diaphragmatic hernia repair 2 months prior.

*

Medications: Cetirizine 10 mg once a day, cyclizine 50 mg 3 times a day, acetaminophen 1 g every 6 hours when required.

*

Allergies: None known

*

Family history: Mother had primary hypothyroidism, father had COPD.

*

Social history: Patient works as a builder, is married with two children age 3 years and 6 years. He smokes 1 pack of cigarettes per day for 10 years, drinks on average 4 to 6 beers per week, and denies use of recreational drugs. There is no history of recent travel abroad or contact with people with illness or infection.

Physical Examination

* General appearance: Slender male who appears comfortable.

*

Vital signs:

* Temperature: 37.8¬∞ C
* Pulse: 112 beats/min
* Respirations: 18/min
* BP: 75/92 mm Hg

*

Skin: Skin is slightly tanned, and his knuckles and nipples appear darkened. There is no obvious jaundice, rash, petechiae, or ecchymoses. Hair is normal.

© 2026 Rao AS et al. *JAMA Network Open*.

*

HEENT: Unremarkable

*

Pulmonary: Bilateral and symmetric air entry, no crackles or wheezes on auscultation of the lungs.

*

Cardiovascular: Normal heart sounds, jugular venous pressure is not seen at 45¬∞, there is no pitting leg edema, and capillary refill is slightly delayed due to cold peripheries.

*

Gastrointestinal: There is an old surgical scar in the epigastric area. Bowel sounds normal. Abdomen soft, with mild generalized tenderness on moderate palpation without rebound tenderness or guarding. No masses. Rectal exam non-tender, heme-negative stool.

*

Genitourinary: Unremarkable

*

Musculoskeletal: No swelling or erythema of joints or extremities. Muscles and joints non-tender, full range of motion. No asterixis.

*

Neurologic: Alert and oriented. Cranial nerves II to XII intact. Muscle tone and power are generally normal and equal bilaterally. Reflexes are +2/4 symmetrically in upper and lower limbs; plantar reflexes are downgoing. Cerebellar tests, including gait and balance, normal.

*

Mental status: Patient reports a recently decreased mood but no suicidal thoughts.

Questions

Question 1 | Type: DIFF

At this time, which of the following possible diagnoses cannot be excluded based on history and physical examination? (You may select more than one option.)

Adrenal insufficiency

Anemia

Cancer

Gastroenteritis

© 2026 Rao AS et al. *JAMA Network Open*.

GI bleeding

Heart failure

Hyperthyroidism

Hypothyroidism

Liver failure

Renal failure

Sepsis

Question 2 | Type: **DIAG**

Which of the following steps are most appropriate for guiding diagnosis and treatment? (You may select more than one option.)

Bedside blood glucose

Complete blood count (CBC)

9 AM serum cortisol

Serum electrolyte concentrations, creatinine, and urea

Serum TSH

Serum free T4 (FT4)

Serum liver function studies (LFTs) and amylase

Serum calcium

Serum troponin

© 2026 Rao AS et al. *JAMA Network Open*.

Serum acetylcholinesterase antibodies

Serum D-dimer level

Plasma metanephrines

Venous blood gas for pH, bicarbonates, and lactate

Urine dipstick

Echocardiography

Chest x-ray

CT scan of the abdomen

Question 3 | Type: **DX**

Results of appropriate testing return and show the following:

Blood:

Test (units)	Result	Results (SI Units)
CBC:		
WCC (cells/mcL)	11,800 cells/mcL	11.8 x 109/L
Hemoglobin (g/L)	14.0 g/dL	140 g/L
Platelets (mcL)	245,000/mcL	245 x 109/L
Glucose (mmol/L)	75.6 mg/dL	4.2mmol/L

Serum:

Na+	130 mEq/L	130 mmol/L
K+	6.2 mEq/L	6.2 mmol/L
Urea nitrogen (BUN)	42 mg/dL	14.9 mmol/L
Creatinine (mg/dL)	2.5 mg/dL	221 micromol/L
9 AM cortisol	1.8 mcg/dL [49 nmol/L]	Normal 6.5-22.5 mcg/dL [180-620 nmol/L]
LFTs and amylase	Normal	

© 2026 Rao AS et al. *JAMA Network Open*.

TSH (mU/L) 0.9 mU/L Normal 0.35-5.5 mU/L
FT4 (ng/dL) 1.6 ng/dL [18.4 pmol/L] Normal 0.9-1.7 ng/dL [10.5-20 pmol/L]

Venous blood gas

Test (units)	Result	Results (SI Units)
pH	7.32	Normal 7.35-7.45
HCO3-	18 mEq/L	18 mmol/L
Lactate (mmol/L)	12.6 mg/dL [1.4 mmol/L]	Normal < 15 mg/dL [< 1.7 mmol/L]

Test (units)	Result
Urine dipstick	Negative
Chest x-ray	Normal

At this time what is the most likely diagnosis? (You may select only one option.)

Anemia

Cortisol insufficiency complicated with an acute kidney injury

Hyperthyroidism

New presentation of type 1 diabetes

Question 4 | Type: **MANG**

At this time, which of the following are the most appropriate next steps to evaluate and manage the patient's cortisol deficiency? (You may select more than one option.)

Serum 17-OH-progesterone

Serum ACTH level

© 2026 Rao AS et al. *JAMA Network Open*.

Serum acetylcholine esterase level

Serum anti-adrenal cortex antibodies

Serum ferritin level

Serum pituitary hormone studies

Plasma renin activity

Overnight dexamethasone suppression test

Short Synacthen test

Hydrocortisone 100 mg IM

0.9% saline 1 L IV over 1 hour

Dopamine 5 mcg/min IV drip

Metoprolol 10 mg IV

© 2026 Rao AS et al. *JAMA Network Open*.

CT/MRI of the adrenal glands

MRI of the pituitary gland

Screening for tuberculosis

Question 5 | Type: **Q**

Results of appropriate studies return and show the following:

Serum:

Test (units)

Result

Results (SI Units)

9AM ACTH (ng/L)

880 pg/mL [194 pmol/L]

Normal 10-60 pg/mL [2.2-13.2 pmol/L]

9 AM testosterone (nmol/L)

320 ng/dL [11.1 nmol/L]

Normal 170-900 ng/dL [5.9-31.2 nmol/L]

Acetylcholine esterase levels

Normal

Anti-adrenal cortex antibodies

Negative

Ferritin level

Normal

LH (U/L)

4.4 mU/mL [4.4U/L]

1.5-9.3 mU/mL [1.5-9.3 U/L]

© 2026 Rao AS et al. *JAMA Network Open*.

FSH 5.9 mU/mL [5.9U/L]

4.4 mU/mL [4.4U/L]

Normal 1.4-18 mU/mL [1.4-18 U/L]

Prolactin (mU/L)

12.3 ng/mL [535 pmol/L

Normal or 3-31 ng/mL [130-1350 pmol/L]

Short Synacthen test (nmol/L)

Flat cortisol response with baseline of 1.4 mcg/dL [39 nmol/L] and 30 minutes level of 1.9 mcg/dL [58 nmol/L]

Normal 30 minutes cortisol above 15.5-18 mcg/dL [430-500 nmol/L] depending on local lab assay

Test (units)

Result

Results (SI Units)

Tuberculosis skin test (PPD)

Negative

Plasma renin activity (upright)

Renin: 600 ng/mL/hour [600 mcg/L/hr]

Normal 2.9-10.8 ng/mL/hour 2.9-10.8 mcg/L/hour

CT scan of the adrenal glands

Reported as bilateral subacute adrenal hemorrhage.

What is the most likely cause of this patient’s adrenal insufficiency? (Please select only one option.)

Autoimmune adrenalitis

© 2026 Rao AS et al. *JAMA Network Open*.

Bilateral adrenal hemorrhage following major surgery

Bilateral granulomatous adrenal lesions

Bilateral metastatic adrenal lesions

Bilateral adrenal hemochromatosis

Hypopituitarism

Question 6 | Type: **MANG**

A diagnosis of Addisonian crisis due to bilateral adrenal hemorrhage is confirmed. At this time, which of the following are appropriate management steps for this patient? (You may select more than one option.)

Watch and wait, with regular monitoring of serum cortisol levels and repeat MRI of the adrenal glands in 6 months

Referral to an endocrine surgeon for bilateral adrenalectomy

0.9% saline IV 250 mL/hr

Desmopressin 50 mcg twice daily

Fludrocortisone 50 mcg once a day orally

© 2026 Rao AS et al. *JAMA Network Open*.

Hydrocortisone 50 mg IV every 6 hours

Oral hydrocortisone (10 mg on waking up, 5 mg at midday and 5 mg around 5 PM) orally

Question 7 | Type: **Q**

Which of the following are causes of primary adrenal insufficiency? (You may select more than one option.)

Adrenal tuberculosis

Adrenoleucodystrophy

Amyloidosis or hemochromatosis

Autoimmune adrenalitis

Bilateral adrenalectomy

Bilateral adrenal hemorrhage

Bilateral metastases to the adrenal glands

Carney complex

© 2026 Rao AS et al. *JAMA Network Open*.

Congenital adrenal hyperplasia

Histoplasmosis

Pendred syndrome

Primary hypothyroidism

Sarcoidosis

Question 8 | Type: **Q**

Which of the following are biochemical abnormalities of adrenal deficiency? (You may select more than one option.)

Hypercalcemia

Hyperglycemia

Hyperkalemia

Hypernatremia

Hypocalcemia

Hypoglycemia

© 2026 Rao AS et al. *JAMA Network Open*.

Hyponatremia

Metabolic acidosis

Metabolic alkalosis

Question 9 | Type: **Q**

Which of the following can provoke an Addisonian crisis? (You may select more than one option.)

Bilateral adrenal hemorrhage

CT scan with contrast

Extensive burn injury

Gastroenteritis

Meningococcal septicemia

Pneumonia

Rapid withdrawal of long-term prednisolone

© 2026 Rao AS et al. *JAMA Network Open*.

Significant weight loss

Strenuous exercise; eg, running a marathon

Surgery with a general anesthetic

Uncomplicated tooth extraction

Question 10 | Type: **Q**

Which of the following are symptoms and signs of primary adrenal insufficiency? (You may select more than one option.)

Abdominal pain

Bone fractures

Hyperpigmentation

Macular rash

Nausea and vomiting

Postural dizziness

Salt craving

© 2026 Rao AS et al. *JAMA Network Open*.

Syncope/cardiovascular collapse

Tiredness

Weight gain

Weight loss

© 2026 Rao AS et al. *JAMA Network Open*.

Case 29: Weight Loss and Palpitations in a 41 Year Old Man

Background

History of Present Illness

A 41-year-old man comes to the office because of a 10-kg weight loss over the past 2 months. He also reports a 1-month history of sweating and palpitations during mild exercise, loose stools, and feeling irritable. Because of these symptoms, he is no longer able to concentrate on his work as an accountant. He also mentions that his eyes recently feel sore, as if somebody has thrown sand in them.

Review of Systems

* General: He reports a 10-kg weight loss over the previous 2 months despite an increase in appetite. No increased thirst. He says he feels hot most of the time but has not had a fever, chills, or night sweats.

*

Skin: No rash or other skin changes.

*

HEENT: His eyes feel gritty and he can spend no more than half an hour working on the computer. No blurry or double vision. No sore throat or neck pain.

*

Pulmonary: He complains of shortness of breath during mild exertion which was never present before. No cough.

*

Cardiovascular: Says he has palpitations whereby he feels as though his heart is racing even during mild exercise, and he has had to stop exercising. No chest pain or discomfort at rest or during exercise.

*

Gastrointestinal: He reports increased stool frequency with semi-formed stools 2 to 3 times per day. Stools are normal color, no blood noted. He denies abdominal pain, nausea, or vomiting.

*

Genitourinary: No difficulty urinating, no increase in frequency or volume or urination, no blood in urine.

*

Musculoskeletal: He reports cramps in his calves at night which he thinks have significantly reduced his exercise performance. No pain or swelling in bones or joints.

© 2026 Rao AS et al. *JAMA Network Open*.

*

Neurologic: He feels his muscles are generally slightly weaker for the last 2 weeks, but weakness is nonfocal.

*

Psychiatric: He complains of being irritable and restless and unable to concentrate at work. He feels depressed although he is concerned about his current symptoms.

Past Medical History

* Medical history: Gilbert syndrome, depression. No known cardiac or pulmonary disease or history of cancer.

*

Surgical history: Appendectomy in childhood.

*

Medications: Sertraline. No dietary supplements or herbal products.

*

Allergies: None.

*

Family history: Father had a myocardial infarction at age 55 years and died of colon cancer at age 68 years. His grandmother had an underactive thyroid.

*

Social history: The patient smokes 10 cigarettes per day, drinks 2 cups of coffee per day, and consumes alcohol only occasionally. He eats a healthy, balanced diet. No illicit drug use. He traveled to Spain more than 6 months ago. No known exposure to people with illness or infection. No sex partners besides his wife for many years. He works as an accountant in a company producing kitchen appliances.

Physical Examination

* General appearance: He is slim and appears somewhat anxious. His hands are sweaty, and hand tremors are noted.

*

Vital signs:

* Temperature: 37.2¬∞ C
* Pulse: 108 beats/min
* Respirations 22/min
* BP 124/80 mm Hg

© 2026 Rao AS et al. *JAMA Network Open*.

*

Skin: Hands and back are sweaty. No rash, erythema, or skin lesions.

*

HEENT: Both eyes are slightly protruding, conjunctivae are red, but there is no eye discharge. Fundi normal bilaterally. He has normal eye movements. There is bilateral, nontender thyroid enlargement without any nodules. No cervical lymphadenopathy. A bruit is noted on auscultation of his thyroid.

*

Pulmonary: Chest is clear on auscultation.

*

Cardiovascular: Heart rate is 108 beats/min and regular. Normal heart sounds.

*

Gastrointestinal: Unremarkable, including normal rectal exam; stool is negative for occult blood.

*

Genitourinary: Unremarkable

*

Musculoskeletal: No muscle wasting or fasciculations. Joints are normal.

*

Neurologic: Alert and oriented to person place and time. There is slightly reduced proximal muscle tone, but quadriceps strength testing is 5/5 bilaterally; testing of other muscles is normal. Reflexes are brisk and symmetric. Sensation to touch is normal. Cranial nerves II to XII intact. Balance and gait normal.

*

Mental status: He appears agitated and restless but responds appropriately to all questions and commands. No flight of ideas, pressured speech, or hallucinations.

Questions

Question 1 | Type: DIFF

At this time, which of the following should be included in the differential diagnoses? (You may select more than one option.)

Bipolar disorder

Diabetes

Drug effects

© 2026 Rao AS et al. *JAMA Network Open*.

Endocarditis

HIV infection

Hyperthyroidism

Malabsorption

Occult malignancy

Tuberculosis

Question 2 | Type: **DIAG**

Which of the following are the most appropriate studies to obtain to guide diagnosis? (You may select more than one option.)

Complete blood count (CBC)

CT scan of the head

Serum ethanol level

Serum glucose level

Chest x-ray

Serum troponin T level

Overnight dexamethasone suppression test

ECG

Serum TSH, free T4, and free T3 levels

© 2026 Rao AS et al. *JAMA Network Open*.

Serum electrolytes, BUN, and creatinine levels

Question 3 | Type: **DX**

Results of appropriate testing return and show the following:

Blood:

Test (units)

Result

Results (SI Units)

CBC:

Hb

14.5 gm/dL

145 g/L

WBC

7,800/mcL

7.8 x 109/L

Platelets

300,000/mcL

300 x 109/L

Random glucose

126 mg/dL

7 mmol/L

Serum:

Test (units)

Result

Results (SI Units)

Na+

138 mEq/L

138 mmol/L

K+

© 2026 Rao AS et al. *JAMA Network Open*.

3.7 mEq/L

3.7 mmol/L

Cl-

101 mEq/L

101 mmol/L

HCO3-

26 mEq/L

26 mmol/L

Creatinine

1.0 mg/dL

89 micromol/L

TSH

<0.01 mU/L

Normal 0.35-5.5 mU/L

FT4

4.2 ng/dL (54 pmol/L)

Normal 0.9-1.7 ng/dL [12-22 pmol/L]

FT3

15.5 pg/mL (24 pmol/L)

Normal 2.3-4.3 pg/mL [3.5-6.5 pmol/L]

ECG: Sinus tachycardia at 110/min, otherwise normal

Chest x-ray: Clear lung fields, normal cardiac silhouette

Given this clinical picture and test results, which of the following is the most likely diagnosis for this patient? (Please select only one option.)

Diabetes

Drug effects

Malabsorption

© 2026 Rao AS et al. *JAMA Network Open*.

Primary hyperthyroidism

Secondary hyperthyroidism

Question 4 | Type: **MANG**

A diagnosis of primary hyperthyroidism is confirmed. Which of the following are the most appropriate next steps to further evaluate the patient's hyperthyroidism? (You may select more than one option.)

Anterior pituitary hormone levels

Serum TSH receptor antibodies

Ophthalmology consultation

CT scan of the neck and thorax

Technetium-99m thyroid uptake scan

Question 5 | Type: **Q**

Image: *29_Q_UT*

Results of appropriate studies return and show the following:

Serum TSH receptor antibodies: Positive (4.1 U/L, normal < 0.4U/L)

Ophthalmology examination: Patient is seen by ophthalmologist and mild thyroid eye disease is confirmed.

Results of technetium-99m thyroid uptake scan are shown:

Which of the following is a correct interpretation of the thyroid scan? (You may select only one option.)

Focal increase in tracer uptake

Generalized increase in tracer uptake

© 2026 Rao AS et al. *JAMA Network Open*.

Normal tracer uptake

Reduced tracer uptake

Question 6 | Type: **DX**

A diagnosis of hyperthyroidism is confirmed. Which of the following is the most likely cause of hyperthyroidism in this patient? (Please select only one option.)

Factitious thyrotoxicosis

Graves disease

Hashimoto's thyroiditis

Iodine-induced thyrotoxicosis

Subacute thyroiditis

Toxic multinodular goiter

TSH producing pituitary adenoma

Question 7 | Type: **MANG**

At this time, which of the following are appropriate management steps for this patient? (You may select more than one option.)

Amiodarone 200 mg daily

Propylthiouracil 150 mg orally 3 times a day

Levothyroxine 100 mcg orally once a day

Methimazole 20 mg orally twice a day

© 2026 Rao AS et al. *JAMA Network Open*.

Potassium iodide solution 0.1 mL orally 3 times a day

Propranolol 40 mg orally 3 times a day

Hypromellose eye drops 4 times a day and Lacrilube eye ointment at night

Radioactive iodine-131 400 MBq

Emergency total thyroidectomy

Smoking cessation program

Question 8 | Type: **Q**

Which of the following are causes of hyperthyroidism? (You may select more than one option.)

Amiodarone-induced thyrotoxicosis

Factitious toxicosis

Graves disease

Hashimoto thyroiditis

Iodine-induced thyrotoxicosis

Non-functioning pituitary adenoma

Partial thyroidectomy

Postpartum thyroiditis

Serotonin reuptake inhibitors

Struma ovari

© 2026 Rao AS et al. *JAMA Network Open*.

Subacute thyroiditis

Thyroid cyst

Toxic multinodular goiter

TSH-producing pituitary adenoma

Question 9 | Type: **Q**

Which of the following are potential complications of Graves disease? (You may select more than one option.)

Atrial fibrillation

Congestive heart failure

Diabetes insipidus

Hypercholesterolemia

Hypocalcemia

Periodic thyrotoxic paralysis

Pretibial myxedema

Psychosis

Secondary hypogonadism

Thyroid eye disease

Thyroid storm

Question 10 | Type: **Q**

Which of the following are presenting features of patients with hyperthyroidism? (You may select more than one option.)

© 2026 Rao AS et al. *JAMA Network Open*.

Bradycardia

Chest pain

Cold intolerance

Constipation

Depression

Diarrhea

Dysmenorrhea

Dyspnea

Heat intolerance

Palpitations

Peripheral edema

Proximal weakness

Psychosis

Sweating

Tiredness

Tremor

Weight loss

© 2026 Rao AS et al. *JAMA Network Open*.

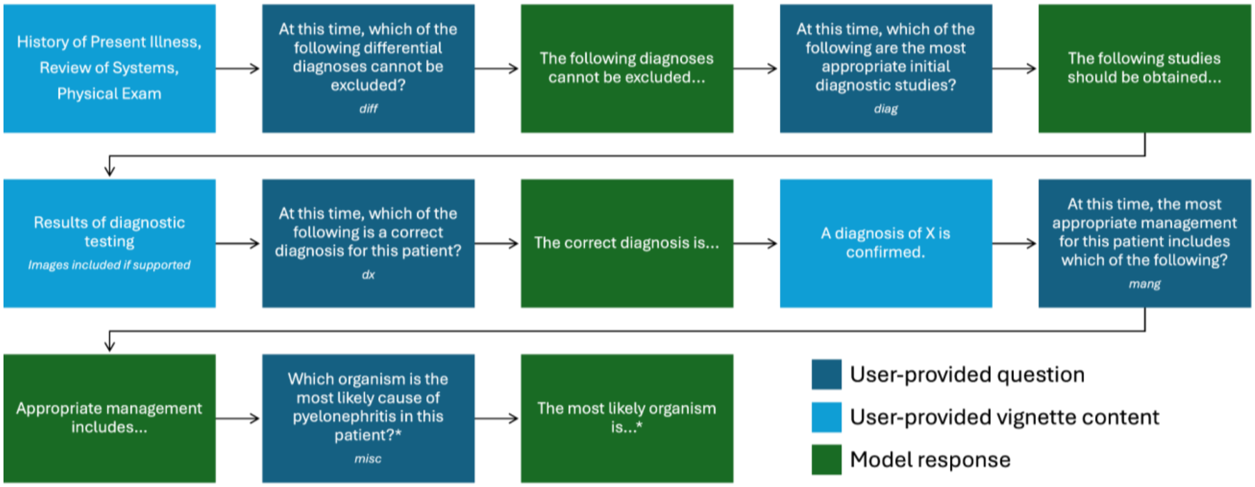

eFigure 1. Stepwise Evaluation Framework for MSD Manual Vignettes. Each MSD Manual vignette was presented stepwise using a direct question-answering methodology to a single model instance, preserving context across the encounter (history of present illness, review of systems, and physical exam). Models were sequentially prompted through five task categories: differential diagnosis (*diff*), diagnostic testing (*diag*), final diagnosis (*dx*), and management (*mang*), and associated miscellaneous clinical reasoning questions (*misc*). Each vignette was tested in triplicate, with independent model instances. Responses were scored by medical student evaluators against MSD Manual answer keys to determine accuracy. *Indicates that *misc* questions vary for each vignette and present general medical knowledge relevant to the vignette.

© 2026 Rao AS et al. *JAMA Network Open*.

MODEL OUTPUT (GPT-5)

- Fondaparinux

Explanation: Fondaparinux is FDA-approved for treatment of acute PE (and DVT) and can be used in the outpatient setting. Enoxaparin, outpatient indication is for DVT without PE; aspirin is not a treatment for PE; dabigatran treats PE but requires initial parenteral anticoagulation and was not specifically approved as initial outpatient monotherapy for PE."

MSD MANUAL INPUT

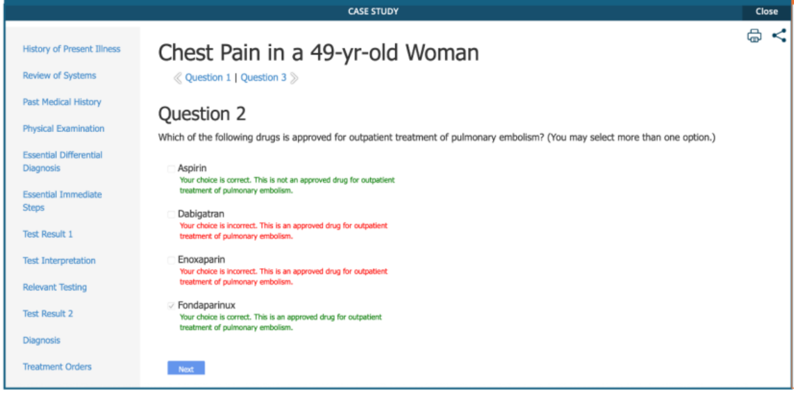

SCORE

1/1: Aspirin is an incorrect answer that the model did not include. The model is awarded the point.

0/1: Dabigatran is a correct answer that the model did not include. The model is *not* awarded the point.

0/1: Enoxaparin is a correct answer that the model did not include. The model is *not* awarded the point.

1/1: Fondaparinux is a correct answer that the model included. The model is awarded the point.

FINAL SCORE: **2/4**

eFigure 2. Model Scoring Schema. Example of model output scoring using the MSD Manual.

© 2026 Rao AS et al. *JAMA Network Open*.

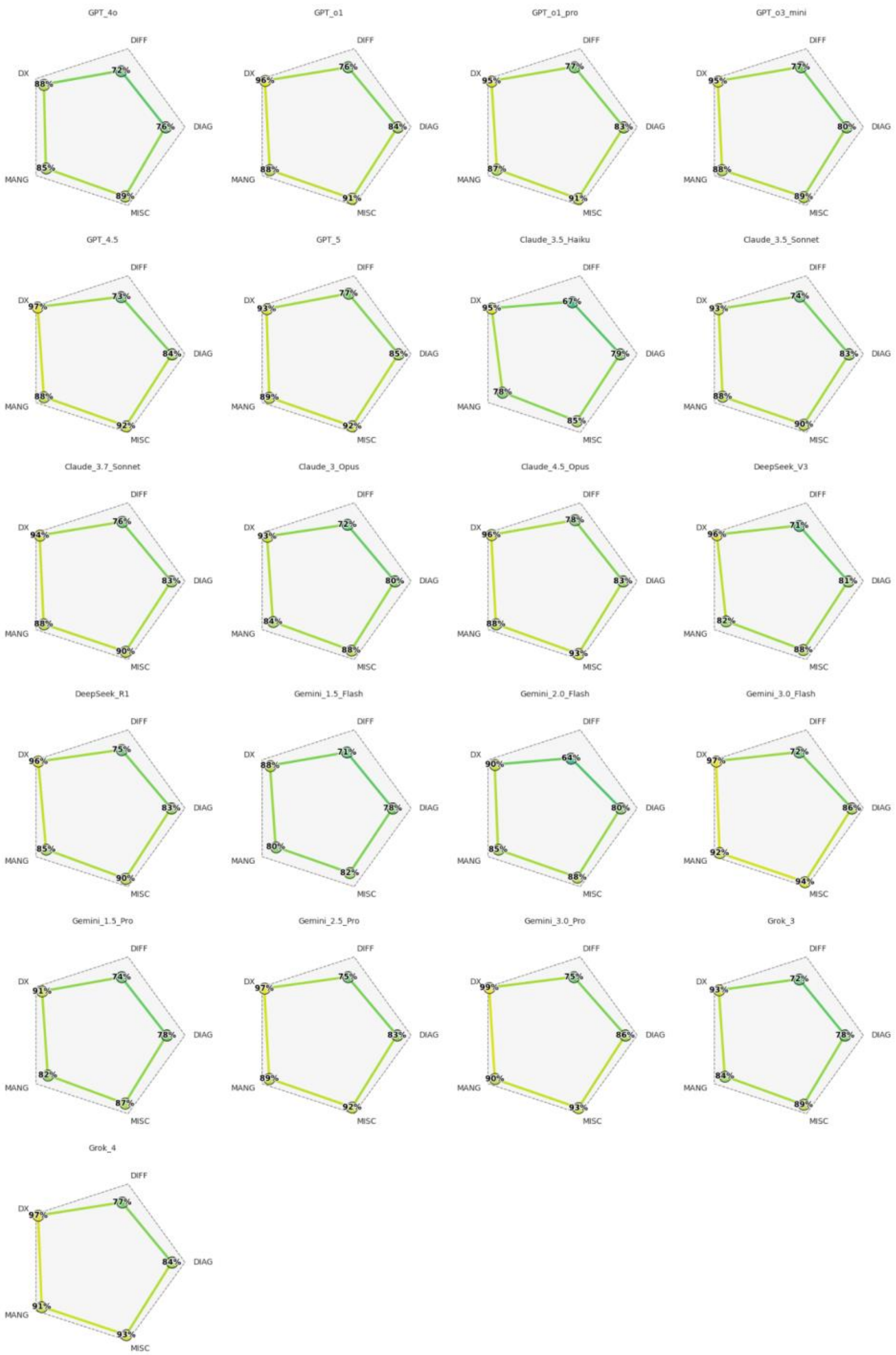

eFigure 3. Radar Plots for All Tested Models. Plots show accuracy across five domains of clinical reasoning: differential diagnosis (diff), diagnostic workup (diag), final diagnosis (dx), management (mang), and miscellaneous reasoning (misc).

© 2026 Rao AS et al. *JAMA Network Open*.

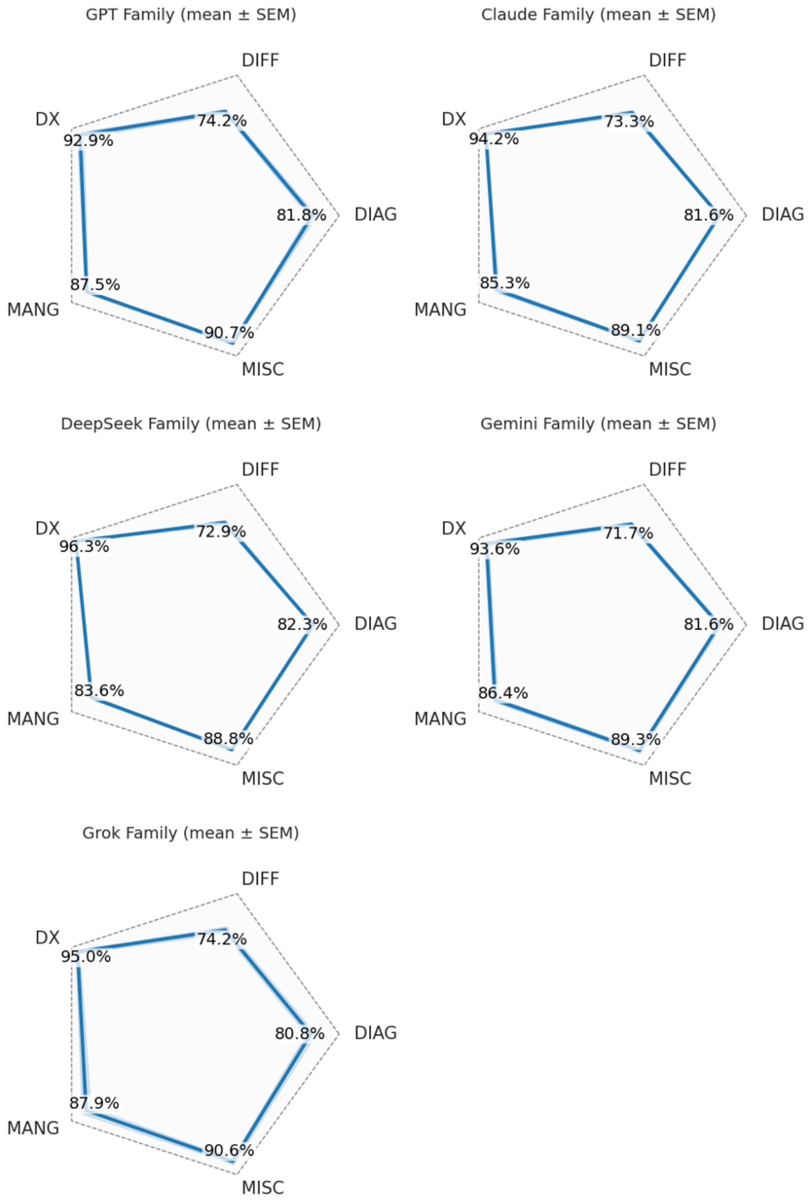

eFigure 4. Radar Plots for LLM Vendors Using Only API-based Models. Plots show accuracy across five domains of clinical reasoning: differential diagnosis (diff), diagnostic workup (diag), final diagnosis (dx), management (mang), and miscellaneous reasoning (misc).

© 2026 Rao AS et al. *JAMA Network Open*.

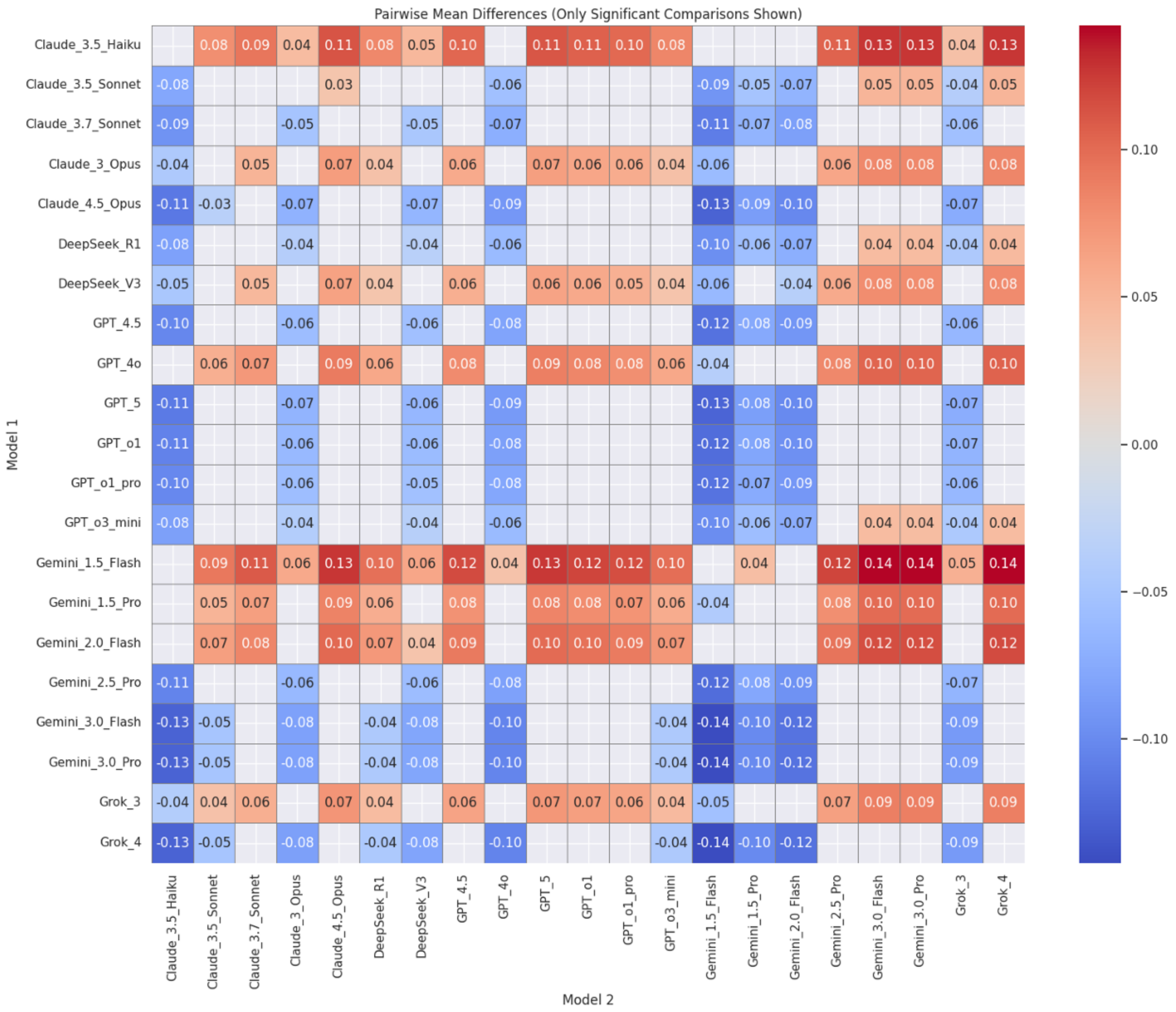

eFigure 5. Heat Map of Pairwise Tukey Comparisons of PrIME-LLM Scores Between Models. Mean differences labeled; only statistically significant comparisons are labeled.

© 2026 Rao AS et al. *JAMA Network Open*.

eTable 2. PrIME-LLM Scores and SEM of Tested Models. The PrIME-LLM Score is calculated as the normalized polygonal area of radar plots showing accuracy across different domains of clinical reasoning, summarizing balanced performance across tasks.

Model Name	Mean ± SEM	95% CI
Claude 3.5 Haiku	0.6507 ± 0.0025	[0.6458, 0.6556]
Claude 3.5 Sonnet	0.7286 ± 0.0034	[0.7219, 0.7353]
Claude 3.7 Sonnet	0.7451 ± 0.0048	[0.7357, 0.7545]
Claude 3 Opus	0.6949 ± 0.0046	[0.6859, 0.7039]
Claude 4.5 Opus	0.7635 ± 0.0017	[0.7602, 0.7668]
DeepSeek R1	0.7335 ± 0.0029	[0.7278, 0.7392]
DeepSeek V3	0.6981 ± 0.0073	[0.6838, 0.7124]
GPT 4.5	0.7534 ± 0.0048	[0.7440, 0.7628]
GPT 4o	0.6734 ± 0.0198	[0.6346, 0.7122]
GPT 5	0.7622 ± 0.0031	[0.7562, 0.7682]
GPT o1	0.7570 ± 0.0032	[0.7508, 0.7632]
GPT o1 pro	0.7521 ± 0.0021	[0.7479, 0.7563]
GPT o3 mini	0.7343 ± 0.0049	[0.7247, 0.7439]
Gemini 1.5 Flash	0.6360 ± 0.0045	[0.6272, 0.6448]
Gemini 1.5 Pro	0.6775 ± 0.0090	[0.6599, 0.6951]
Gemini 2.0 Flash	0.6615 ± 0.0055	[0.6507, 0.6723]
Gemini 2.5 Pro	0.7562 ± 0.0055	[0.7454, 0.7670]
Gemini 3.0 Flash	0.7777 ± 0.0059	[0.7660, 0.7894]
Gemini 3.0 Pro	0.7776 ± 0.0046	[0.7686, 0.7866]
Grok 3	0.6898 ± 0.0019	[0.6861, 0.6935]
Grok 4	0.7783 ± 0.0051	[0.7683, 0.7883]

© 2026 Rao AS et al. *JAMA Network Open*.

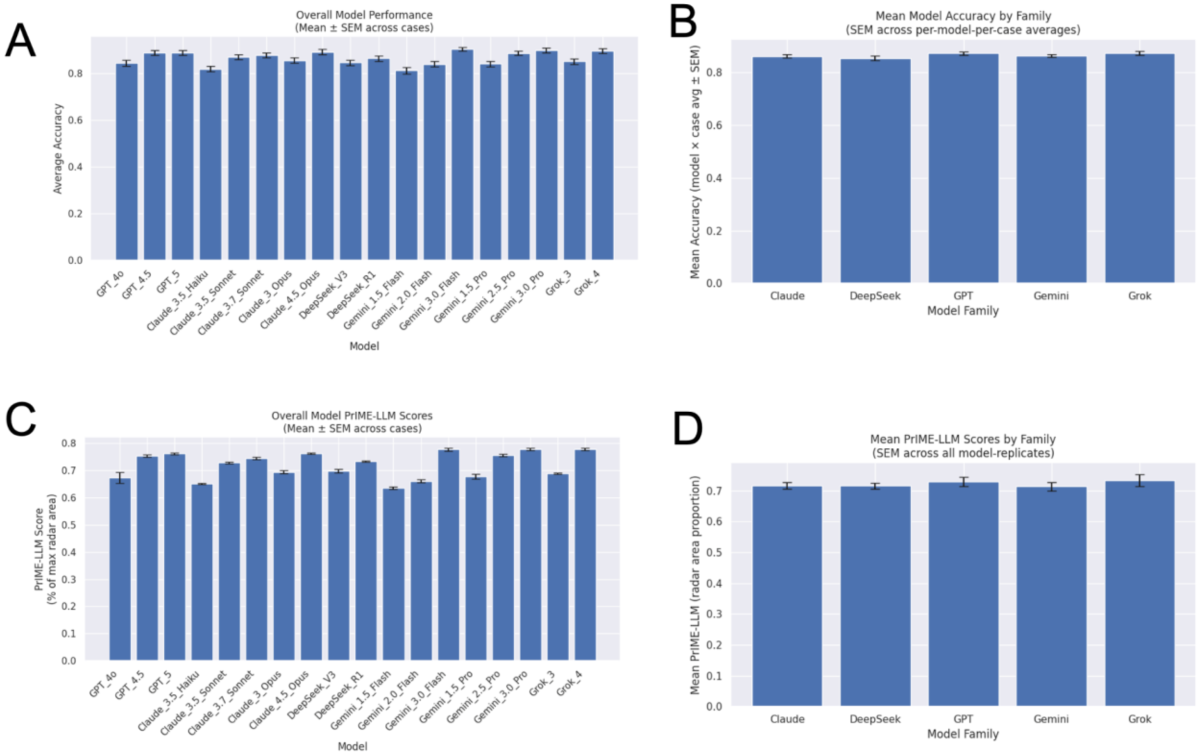

eFigure 6. Average Accuracy and PrIME-LLM Calculations by Model and Family with API-only Models. (A) ANOVA test comparing all models: $F(17, 476) = 19.08$. $p < 0.001$. (B) ANOVA test comparing LLM vendors: $F(4, 517) = 1.09$, $p = 0.36$. (C) ANOVA testing comparing all models using PrIME: $F(17, 36) = 50.85$, $p < 0.001$. (D) ANOVA test comparing LLM vendors using PrIME: $F(4, 49) = 0.30$, $p = 0.88$.

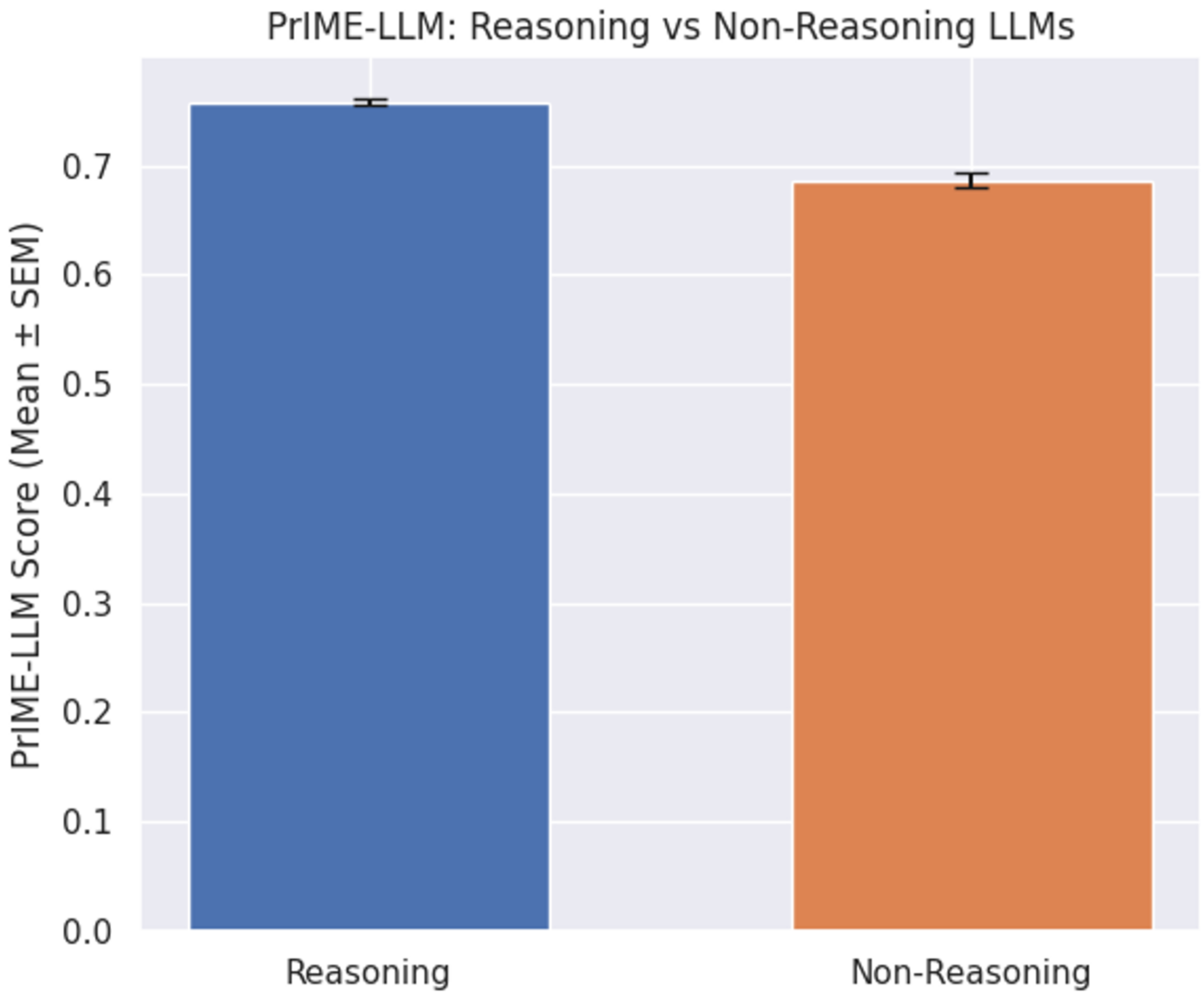

eFigure 7. PrIME-LLM Score Comparison Between Reasoning-Designated Models and Non-Reasoning-Designated Models. T-test: t = 9.980, p = 1.95494e-12. Reasoning Mean = 0.75796 (SEM = 0.00291, N = 33). Non-Reasoning Mean = 0.68639 (SEM = 0.00655, N = 30). Effect sizes: Cohen's d: 2.597. Hedges' g: 2.565 (95% CI: 1.898, 3.233). CLES (P[R>N]): 0.995.

© 2026 Rao AS et al. *JAMA Network Open*.

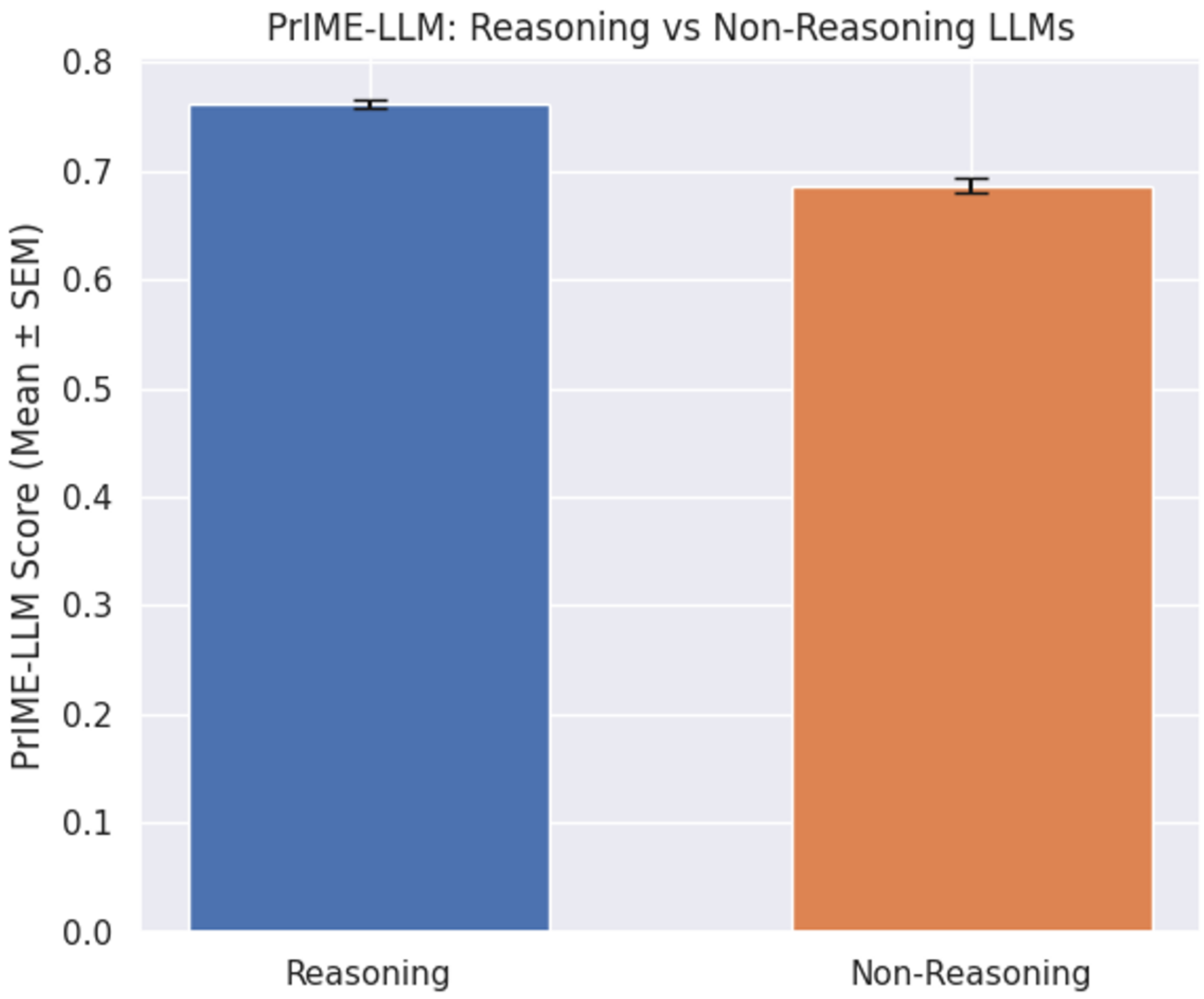

eFigure 8. PrIME-LLM Score Comparison Between Reasoning-Designated Models and Non-Reasoning-Designated Models using Only API-Based Models. T-test: t = 10.176, p = 4.87101e-13. Reasoning Mean = 0.76177 (SEM = 0.00345, N = 24). Non-Reasoning Mean = 0.68639 (SEM = 0.00655, N = 30). Effect sizes: Cohen's d: 2.593. Hedges' g: 2.555 (95% CI: 1.834, 3.277). CLES (P[R>N]): 0.955.

© 2026 Rao AS et al. *JAMA Network Open*.

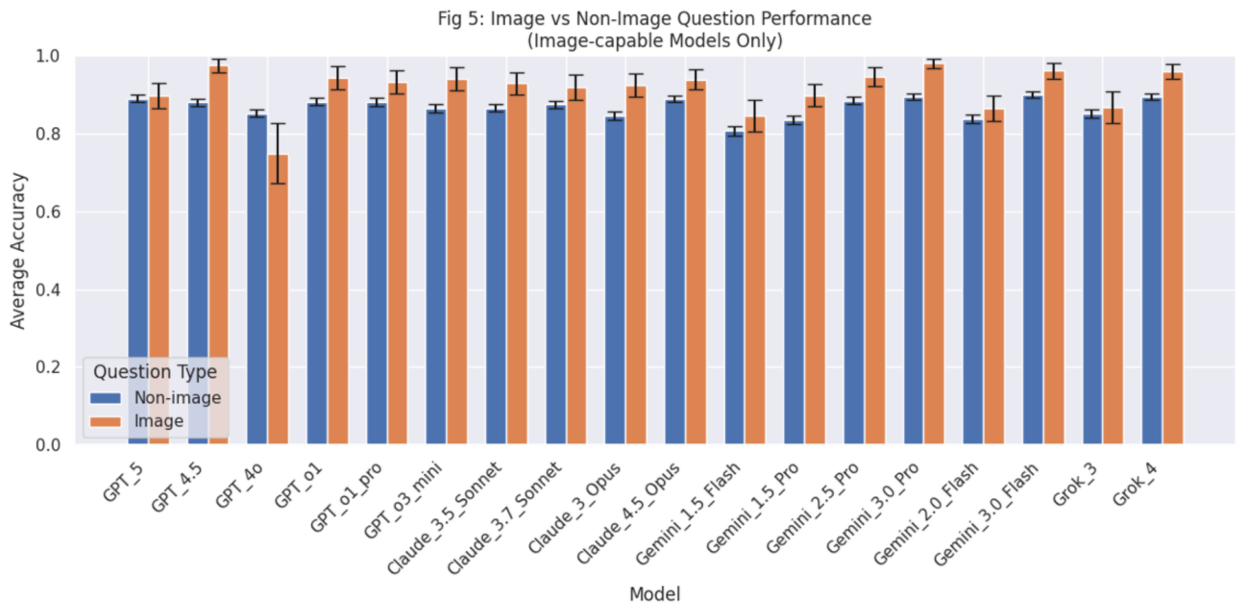

eFigure 9. PrIME-LLM Score Comparison Between Questions Involving Use of an Image and Text-Only Questions

© 2026 Rao AS et al. *JAMA Network Open*.

eTable 3. Multivariable Linear Regression Modeling the Probability of Question-Level Accuracy. Shown are regression coefficients and 95% confidence intervals for model, question type, patient age, patient sex, and LLM “reasoning” capability. The regression was performed on per-question accuracy (proportion-correct) for each (case, question, model), including fixed effects for LLM reasoning group, question type, and patient demographics. Positive coefficients indicate features associated with higher per-question accuracy. Model families not supporting certain input types were excluded for those items. *N = 5344 (29 groups; min group size = 126, max = 270, M = 184.3); dependent variable = PropCorrect; method = REML; scale = 0.0185; log-likelihood = 2989.1518; model converged.* * $p < 0.01$, ** $p < 0.001$.

**Predictor**	**Coefficient β [95% CI]**	***z*-value**
**Intercept**	0.795** [0.732, 0.858]	24.694
**Sex: Male**	0.019 [-0.026, 0.065]	0.832
**Type: Diff**	–0.077** [–0.092, –0.063]	–10.366
**Type: Dx**	0.131** [0.118, 0.145]	19.134
**Type: Mang**	0.053** [0.039, 0.066]	7.542
**Type: Misc**	0.083** [0.071, 0.094]	14.199
**Model: Reasoning**	0.041** [0.034, 0.048]	11.013
**Age**	–0.000 [–0.001, 0.001]	–0.650

© 2026 Rao AS et al. *JAMA Network Open*.

eTable 4. Multivariable Linear Regression of PrIME-LLM Scores by Model Features.

Shown are regression coefficients and 95% confidence intervals from a linear model predicting PrIME-LLM scores (the normalized area under the radar plot summarizing model performance across question types) for each model and replicate. Predictors included LLM "reasoning" group and model family. Positive coefficients indicate model features associated with higher overall multidimensional clinical performance. Note that PrIME-LLM scores are aggregated across all cases and question types, so patient-specific covariates (age, sex) are not included in this analysis. n = 63, adjusted R^2 = 0.629, $F(1, 61)$ = 5.78e-15, * p < 0.01, ** p < 0.001.

**Predictor**	**Coefficient β [95% CI]**	***t*-value**
**Intercept**	0.6864** [0.676, 0.696]	136.426
**Model: Reasoning**	0.0716** [0.058, 0.085]	10.296

© 2026 Rao AS et al. *JAMA Network Open*.

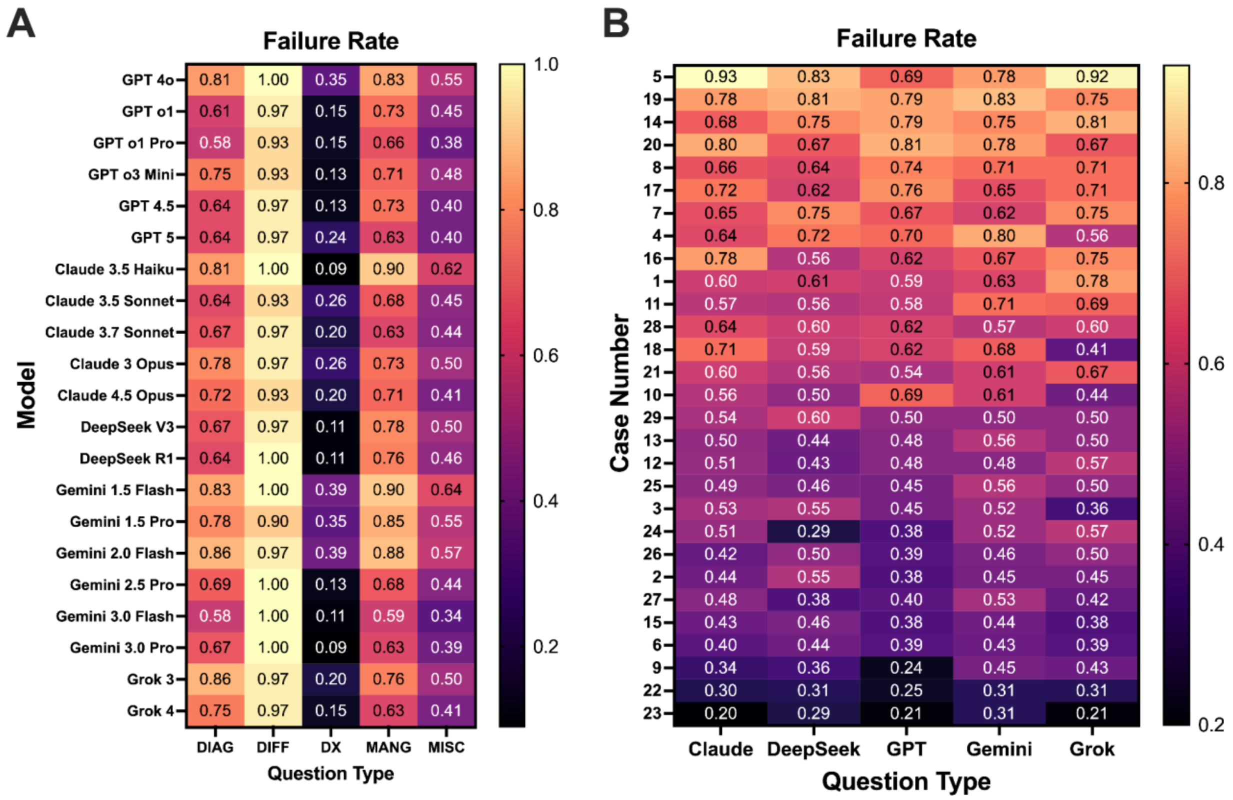

eFigure 10. Failure Rates of Tested Models by Question Type and Case Number. (A) Failure rate by question type, defined as the proportion of questions not answered fully correctly. (B) Failure rate by case across all 29 tested vignettes.

© 2026 Rao AS et al. *JAMA Network Open*.

eTable 5. Failure Rates by Age Group and Question Type. All tested LLM vendors are shown.

Model	Family Type	AgeBin	FailRate
Claude	DIAG	0-17	0.200000
Claude	DIAG	18-39	0.745455
Claude	DIAG	40-64	0.833333
Claude	DIAG	65+	0.672727
Claude	DIFF	0-17	1.000000
Claude	DIFF	18-39	0.960000
Claude	DIFF	40-64	0.963636
Claude	DIFF	65+	0.942857
Claude	DX	0-17	0.100000
Claude	DX	18-39	0.246154
Claude	DX	40-64	0.133333
Claude	DX	65+	0.261538
Claude	MANG	0-17	0.266667

© 2026 Rao AS et al. *JAMA Network Open*.

Claude	MANG	18-39	0.836364
Claude	MANG	40-64	0.755556
Claude	MANG	65+	0.711111
Claude	MISC	0-17	0.228571
Claude	MISC	18-39	0.441176
Claude	MISC	40-64	0.556098
Claude	MISC	65+	0.495652
DeepSeek	DIAG	0-17	0.000000
DeepSeek	DIAG	18-39	0.545455
DeepSeek	DIAG	40-64	0.791667
DeepSeek	DIAG	65+	0.727273
DeepSeek	DIFF	0-17	1.000000
DeepSeek	DIFF	18-39	1.000000
DeepSeek	DIFF	40-64	1.000000
DeepSeek	DIFF	65+	0.928571

© 2026 Rao AS et al. *JAMA Network Open*.

DeepSeek	DX	0-17	0.250000
DeepSeek	DX	18-39	0.115385
DeepSeek	DX	40-64	0.111111
DeepSeek	DX	65+	0.076923
DeepSeek	MANG	0-17	0.500000
DeepSeek	MANG	18-39	0.772727
DeepSeek	MANG	40-64	0.777778
DeepSeek	MANG	65+	0.833333
DeepSeek	MISC	0-17	0.285714
DeepSeek	MISC	18-39	0.455882
DeepSeek	MISC	40-64	0.536585
DeepSeek	MISC	65+	0.478261
GPT	DIAG	0-17	0.166667
GPT	DIAG	18-39	0.651515
GPT	DIAG	40-64	0.791667

© 2026 Rao AS et al. *JAMA Network Open*.

GPT	DIAG	65+	0.651515
GPT	DIFF	0-17	0.916667
GPT	DIFF	18-39	0.950000
GPT	DIFF	40-64	1.000000
GPT	DIFF	65+	0.928571
GPT	DX	0-17	0.166667
GPT	DX	18-39	0.153846
GPT	DX	40-64	0.212963
GPT	DX	65+	0.205128
GPT	MANG	0-17	0.277778
GPT	MANG	18-39	0.848485
GPT	MANG	40-64	0.750000
GPT	MANG	65+	0.629630
GPT	MISC	0-17	0.238095
GPT	MISC	18-39	0.406863

© 2026 Rao AS et al. *JAMA Network Open*.

GPT	MISC	40-64	0.508130
GPT	MISC	65+	0.442029
Gemini	DIAG	0-17	0.416667
Gemini	DIAG	18-39	0.757576
Gemini	DIAG	40-64	0.805556
Gemini	DIAG	65+	0.696970
Gemini	DIFF	0-17	0.916667
Gemini	DIFF	18-39	0.966667
Gemini	DIFF	40-64	0.984848
Gemini	DIFF	65+	1.000000
Gemini	DX	0-17	0.250000
Gemini	DX	18-39	0.243590
Gemini	DX	40-64	0.203704
Gemini	DX	65+	0.294872
Gemini	MANG	0-17	0.500000

© 2026 Rao AS et al. *JAMA Network Open*.

Gemini	MANG	18-39	0.833333
Gemini	MANG	40-64	0.777778
Gemini	MANG	65+	0.703704
Gemini	MISC	0-17	0.190476
Gemini	MISC	18-39	0.436275
Gemini	MISC	40-64	0.548780
Gemini	MISC	65+	0.550725
Grok	DIAG	0-17	0.750000
Grok	DIAG	18-39	0.863636
Grok	DIAG	40-64	0.875000
Grok	DIAG	65+	0.681818
Grok	DIFF	0-17	1.000000
Grok	DIFF	18-39	0.900000
Grok	DIFF	40-64	1.000000
Grok	DIFF	65+	1.000000

© 2026 Rao AS et al. *JAMA Network Open*.

Grok	DX	0-17	0.000000
Grok	DX	18-39	0.115385
Grok	DX	40-64	0.222222
Grok	DX	65+	0.192308
Grok	MANG	0-17	0.166667
Grok	MANG	18-39	0.863636
Grok	MANG	40-64	0.722222
Grok	MANG	65+	0.611111
Grok	MISC	0-17	0.142857
Grok	MISC	18-39	0.382353
Grok	MISC	40-64	0.560976
Grok	MISC	65+	0.456522

© 2026 Rao AS et al. *JAMA Network Open*.

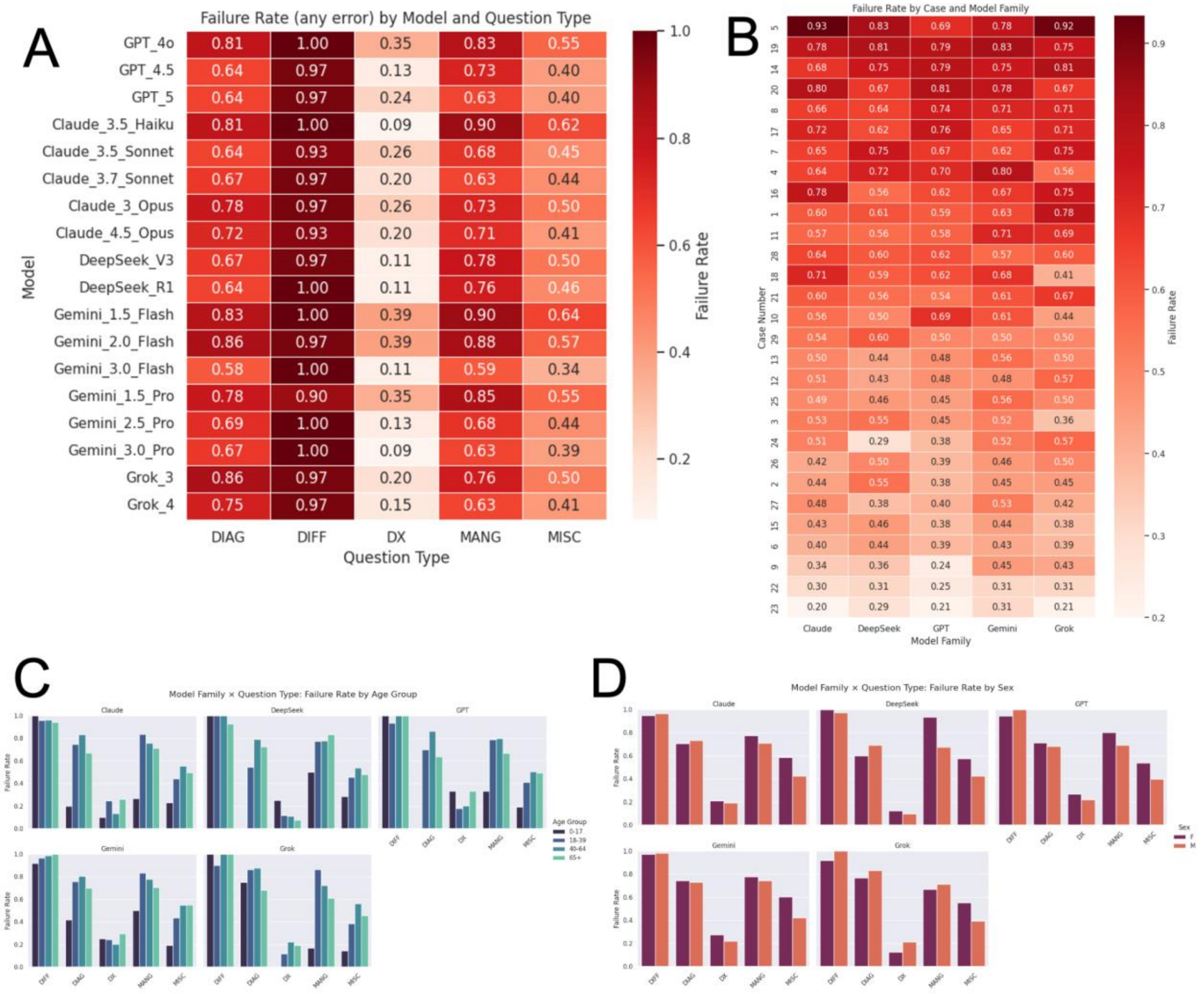

eFigure 11. Failure Rates of Tested Models by Question Type and Case Number Including Only API-Based Models. (A) Failure rate by question type, defined as the proportion of questions not answered fully correctly. (B) Failure rate by case across all 29 tested vignettes. (C) Failure rate by age group, defined as the proportion of questions not answered fully correctly. (D) Failure rate by patient sex.

© 2026 Rao AS et al. *JAMA Network Open*.

eTable 6. Table of Failure Rates by Patient Sex and Question Type. All tested LLM vendors are shown.

Model	Family Type	Sex	FailRate
Claude	DIAG	F	0.706667
Claude	DIAG	M	0.733333
Claude	DIFF	F	0.950000
Claude	DIFF	M	0.966667
Claude	DX	F	0.210000
Claude	DX	M	0.192308
Claude	MANG	F	0.773333
Claude	MANG	M	0.707692
Claude	MISC	F	0.584615
Claude	MISC	M	0.424242
DeepSeek	DIAG	F	0.600000
DeepSeek	DIAG	M	0.690476
DeepSeek	DIFF	F	1.000000

© 2026 Rao AS et al. *JAMA Network Open*.

DeepSeek	DIFF	M	0.972222
DeepSeek	DX	F	0.125000
DeepSeek	DX	M	0.096154
DeepSeek	MANG	F	0.933333
DeepSeek	MANG	M	0.673077
DeepSeek	MISC	F	0.576923
DeepSeek	MISC	M	0.424242
GPT	DIAG	F	0.677778
GPT	DIAG	M	0.666667
GPT	DIFF	F	0.958333
GPT	DIFF	M	0.962963
GPT	DX	F	0.200000
GPT	DX	M	0.185897
GPT	MANG	F	0.800000
GPT	MANG	M	0.666667

© 2026 Rao AS et al. *JAMA Network Open*.

GPT	MISC	F	0.512821
GPT	MISC	M	0.401515
Gemini	DIAG	F	0.744444
Gemini	DIAG	M	0.730159
Gemini	DIFF	F	0.972222
Gemini	DIFF	M	0.981481
Gemini	DX	F	0.275000
Gemini	DX	M	0.217949
Gemini	MANG	F	0.777778
Gemini	MANG	M	0.743590
Gemini	MISC	F	0.602564
Gemini	MISC	M	0.421717
Grok	DIAG	F	0.766667
Grok	DIAG	M	0.833333
Grok	DIFF	F	0.916667

© 2026 Rao AS et al. *JAMA Network Open*.

Grok	DIFF	M	1.000000
Grok	DX	F	0.125000
Grok	DX	M	0.211538
Grok	MANG	F	0.666667
Grok	MANG	M	0.711538
Grok	MISC	F	0.551282
Grok	MISC	M	0.393939

© 2026 Rao AS et al. *JAMA Network Open*.

eReferences

1. GPT-4o. OpenAI Platform. Accessed July 31, 2025. https://platform.openai.com/docs/models/gpt-4o
2. o1. OpenAI Platform. Accessed July 31, 2025. https://platform.openai.com/docs/models/o1
3. o1-pro. OpenAI Platform. Accessed July 31, 2025. https://platform.openai.com/docs/models/o1-pro
4. o3-mini. OpenAI Platform. Accessed July 31, 2025. https://platform.openai.com/docs/models/o3-mini
5. Introducing GPT-4.5. OpenAI. February 27, 2025. Accessed July 31, 2025. https://openai.com/index/introducing-gpt-4-5/
6. Introducing GPT-5. OpenAI. August 7, 2025. Accessed August 9, 2025. https://openai.com/index/introducing-gpt-5/
7. Models Overview. Anthropic. Accessed July 31, 2025. https://docs.anthropic.com/en/docs/about-claude/models/overview
8. Models & Pricing. DeepSeek API Docs. Accessed July 31, 2025. https://api-docs.deepseek.com/quick_start/pricing
9. Gemini Models. Google AI for Developers. Accessed July 31, 2025. https://ai.google.dev/gemini-api/docs/models
10. Grok 3 Beta — The Age of Reasoning Agents. X AI. February 19, 2025. Accessed July 31, 2025. https://x.ai/news/grok-3
11. Grok 4. xAI. July 9, 2025. Accessed January 17, 2026. https://x.ai/news/grok-4

© 2026 Rao AS et al. *JAMA Network Open*.
