# Supplementary material for: Large Language Model Performance and Clinical Reasoning Tasks
Source: JAMA Netw Open. 2026 Apr 13;9(4):e264003. doi: 10.1001/jamanetworkopen.2026.4003 (PMC13077515; doi:10.1001/jamanetworkopen.2026.4003)
Supplement: Supplement 2. — Data Sharing Statement [file jamanetwopen-e264003-s002.pdf]

Data Sharing Statement

Rao. Large Language Model Performance and Clinical Reasoning Tasks. *JAMA Netw Open*. Published March 30, 2026. doi:10.1001/jamanetworkopen.2026.4003

Data
Data available: Yes
Data types: Data (not involving human participants)
How to access data: Data can be made available by request to kesmail@hms.harvard.edu.
When available: With publication

Supporting Documents
Document types: None

Additional Information
Who can access the data: Data will be made available to all those who request it.
Types of analyses: Data will be made available for all analyses.
Mechanisms of data availability: Data will be made available without investigator support.
Any additional restrictions: There are no restrictions on the use of this data.
